# Structure-guided discovery of a small molecule inhibitor of SARS-CoV-2 main protease with potent *in vitro* and *in vivo* antiviral activities

**DOI:** 10.1128/jvi.01001-25

**Published:** 2025-11-14

**Authors:** Shuofeng Yuan, Juan Wang, Xiaohong Sang, Yubin Xie, Yong Feng, Vincent Kwok-Man Poon, Chris Chung-Sing Chan, Jessica Oi-Ling Tsang, Kenn Ka-Heng Chik, Jiao Zhou, Yan Xu, Pu Han, Wenyan Zheng, Lifeng Fu, Lina Sirui Mihara Huang, Meixian Wu, Jing An, Kwok-Yung Yuen, Jianxun Qi, Ziwei Huang, Jasper Fuk-Woo Chan

**Affiliations:** 1 State Key Laboratory of Emerging Infectious Diseases, Carol Yu Centre for Infection, Department of Microbiology, School of Clinical Medicine, Li Ka Shing Faculty of Medicine, The University of Hong Kong, Pokfulam, Hong Kong Special Administrative Region, China; 2 InnoHK (Centre for Virology, Vaccinology and Therapeutics), Hong Kong Science and Technology Park, Hong Kong Special Administrative Region, China; 3 Department of Infectious Diseases and Microbiology, The University of Hong Kong-Shenzhen Hospital 444333, Shenzhen, China; 4 Guangzhou Laboratory 612039, Guangzhou, China; 5 Pandemic Research Alliance at The University of Hong Kong, Hong Kong Special Administrative Region, China; 6 School of Life Sciences, Tsinghua University 98441, Beijing, China; 7 Ciechanover Institute of Precision and Regenerative Medicine, Shenzhen Futian Biomedical Innovation R&D Center, School of Medicine, The Chinese University of Hong Kong 407605, Shenzhen, China; 8 CAS Key Laboratory of Pathogen Microbiology and Immunology, Institute of Microbiology, Chinese Academy of Sciences 85387, Beijing, China; 9 Division of Infectious Diseases and Global Public Health, Department of Medicine, School of Medicine, University of California at San Diego, La Jolla, California, USA; 10 State Key Laboratory for Conservation and Utilization of Subtropical Agro-Bioresources, Guangxi University 12664, Nanning, China; 11 Academician Workstation of Hainan Province, Hainan Medical University-The University of Hong Kong Joint Laboratory of Tropical Infectious Diseases, Hainan Medical University 12455, Haikou, China; 12 Department of Microbiology, Queen Mary Hospital, Pokfulam, Hong Kong Special Administrative Region, China; St Jude Children's Research Hospital, Memphis, Tennessee, USA

**Keywords:** protease, antiviral, SARS-CoV-2

## Abstract

The severe acute respiratory syndrome coronavirus 2 (SARS-CoV-2) main protease (M^pro^) is a clinically validated target for the treatment of coronavirus disease 2019 (COVID-19). Here, we report the hit-to-lead discovery of a new nitrile-based inhibitor, **H135**, of SARS-CoV-2 M^pro^ from a series of chemical modifications of a nitrile-warhead-containing compound guided by its 1.8 Å high-resolution crystal structure in complex with SARS-CoV-2 M^pro^. **H135** exhibited potent and selective inhibition of SARS-CoV-2 M^pro^ with IC_50_ of 12.7 nM and exerted broad anti-SARS-CoV-2 activity in VeroE6-TMPRSS2 cells. Furthermore, **H135**-treated golden Syrian hamsters demonstrated significantly lower respiratory tract viral burden compared with solvent-treated control hamsters. **H135** may serve as a new lead for developing antiviral therapeutics for SARS-CoV-2 infection.

**IMPORTANCE:**

In this study, a structure-guided hit-to-lead strategy was employed to develop a nanomolar potent small molecule inhibitor H135 of SARS-CoV-2 M^pro^ with strong anti-SARS-CoV-2 infection activity in cell cultures and animals. H135 may serve as a new lead for developing antiviral agents targeting the virus’s main protease M^pro^.

## INTRODUCTION

The main protease (M^pro^) of SARS-CoV-2, also known as 3-chymotrypsin-like (3Cl) protease, is a clinically validated antiviral target for treating SARS-CoV-2 infection (1). It is a cysteine protease with Cys145-His41 catalytic dyad in the substrate-binding site, which cleaves viral polyproteins (pp1a and pp1ab) into 16 non-structural proteins (NSP1-NSP16) essential for viral replication (2). SARS-CoV-2 M^pro^ is a homodimer with each monomer formed by domain I (residues 10–99), domain II (residues 100–182), and domain III (residues 198–303) (3, 4). The amino acids of peptide substrate are numbered as P3, P2, P1, P1′, P2′, and P3′. M^pro^ cleaves the amide bond between P1 and P1′. The peptide substrate binding site containing Cys145-His41 catalytic dyad is located in domains I and II, with subsites numbered S3, S2, S1, S1′, S2′, and S3′, respectively (5, 6).

**Fig 1:**
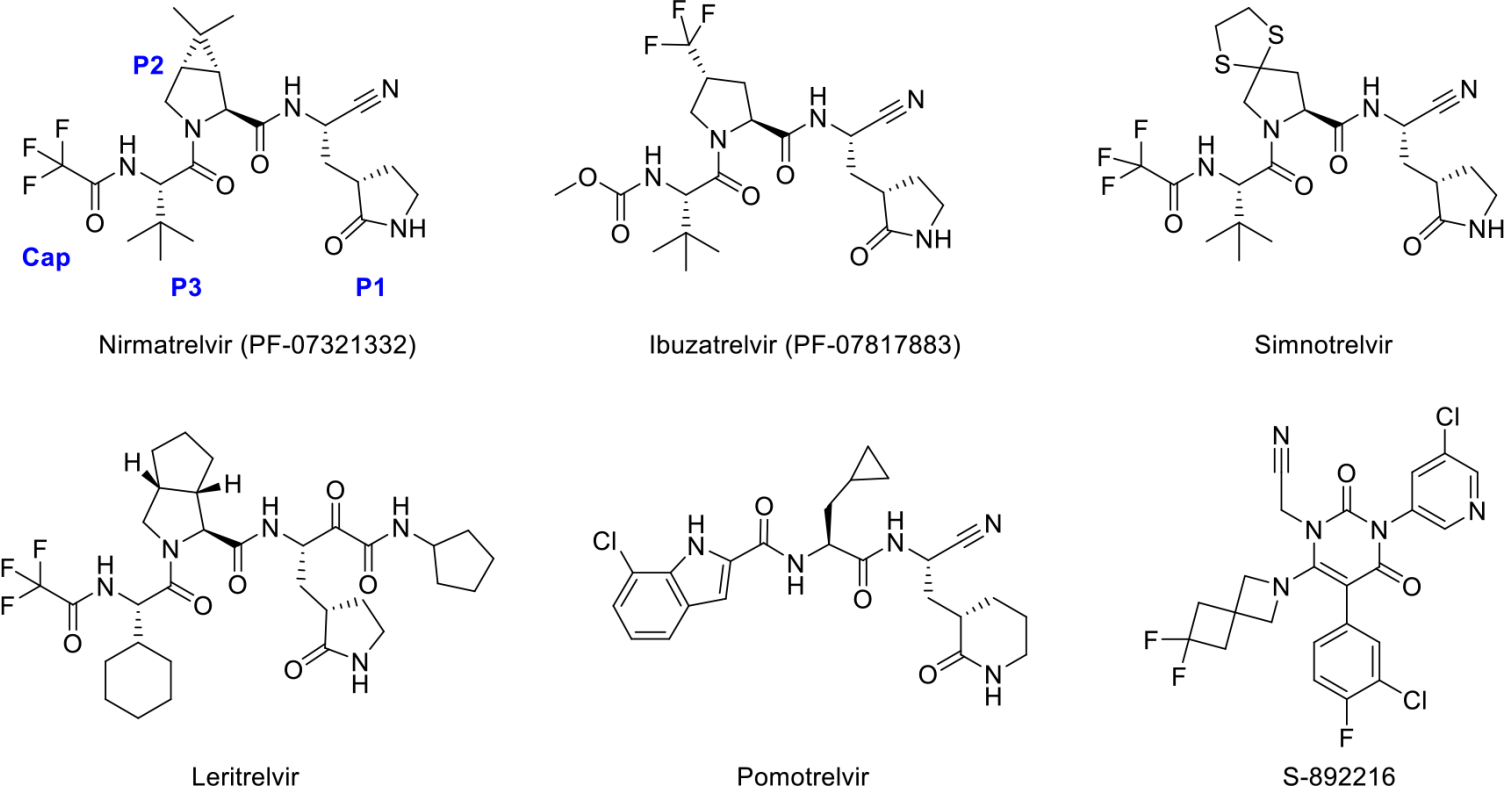
Chemical structures of nirmatrelvir (PF-07321332), ibuzatrelvir (PF-07817883), simnotrelvir, leritrelvir, pomotrelvir, and S-892216.

Many covalent and noncovalent M^pro^ inhibitors have been developed, mostly targeting the substrate binding site or active site of M^pro^ (1, 6, 7). Covalent M^pro^ inhibitors bind to the active site, with their warheads reacting with the sulfur atom of Cys145 of the enzyme’s catalytic dyad. Nirmatrelvir (PF-07321332), a nitrile-based covalent SARS-CoV-2 M^pro^ inhibitor, has been approved clinically to treat COVID-19 in combination with ritonavir (8, 9), whileensitrelvir (S-217622) is a noncovalent M^pro^ inhibitor approved in Japan and Singapore (10). Recently, Pfizer reported the discovery of a second generation of SARS-CoV-2 M^pro^ inhibitor, ibuzatrelvir (PF-07817883), with improved oral pharmacokinetics based on modifications to the P2 and P4 groups of PF-07321332 (Fig. 1) (11). S-892216, another second-generation SARS-CoV-2 M^pro^ inhibitor, exhibits higher activity and superiority to S-217622, designed by introducing nitrile warhead and modifications on substituents targeting S2 and S1′ pocket of S-217622 analogs (12). Other peptidomimetic SARS-CoV-2 M^pro^ inhibitors, such as simnotrelvir, leritrelvir, and pomotrelvir, are in clinical trials or are clinically used in other countries (Fig. 1) (1, 13).

Our laboratories have been interested in developing covalent SARS-CoV-2 M^pro^ inhibitors. Previously, we reported a ketoamide-based SARS-CoV-2 M^pro^ inhibitor, named compound 17, which displayed potent anti-SARS-CoV-2 activity in plaque reduction assay with the EC_50_ of 1.28 µM (14). Subsequently, we have developed more potent ketoamide- and aldehyde-based analogs using compound 17 as the starting template that have more potent SARS-CoV-2 M^pro^ inhibition and anti-SARS-CoV-2 activity (submitted for publication). In this study, we investigated a class of analogs using nitrile as the cysteine-reactive warhead instead of the ketoamide and aldehyde mentioned above. As an electrophilic covalent reactive warhead, the nitrile group has been successfully utilized in serine protease DPP4 (15), cysteine protease Cathepsin K inhibitors (16), and the clinically approved PF-07321332 for SARS-CoV-2 M^pro8^. The nitrile-based inhibitors are noted for enhanced solubility and ease of synthetic scale-up (8). In the present study, we describe our efforts to conduct a structure-guided hit-to-lead optimization and identify a nanomolar nitrile-based M^pro^ inhibitor **H135** with potent antiviral activity both *in vitro* and *in vivo*. We started with using the chemical structure of our previously developed ketoamide- and aldehyde-based analogs as the design template and incorporating a different warhead of nitrile. The resulting hit compound was subjected to structure determination of its co-crystal structure with the enzyme. Based on the structural information, a series of synthetic modifications were made at different positions of the hit compound’s structure to derive the final lead compound, **H135**.

## RESULTS AND DISCUSSION

### Synthesis and biological activity of compound H109 containing a nitrile warhead and its co-crystal structure in complex with SARS-CoV-2 M^pro^

The synthetic route and experimental details for the preparations of **H109** and other target compounds shown in Tables 1 and 2 are provided in supplementary information.

**TABLE 1:** M^pro^ inhibition of P2 modified compounds

		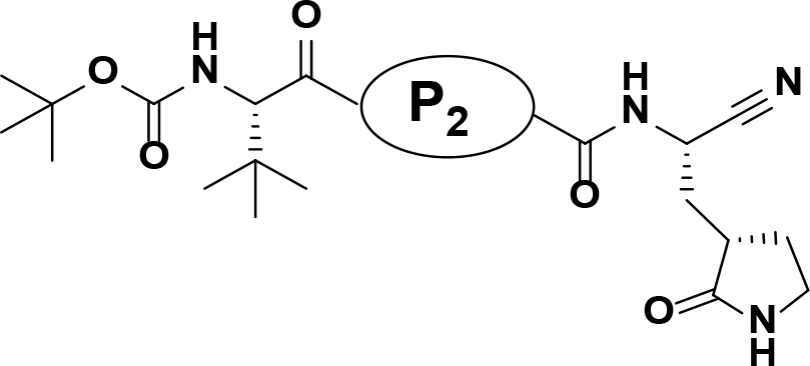			
Code	P_2_	M^pro^ IC_50_	Code	P_2_	M^pro^ IC_50_
**H109**	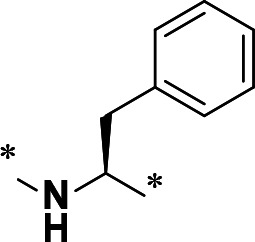	407.0 ± 5.6 nM	**H116**	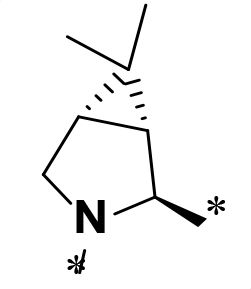	161.8 ± 7.3 nM
**H119**	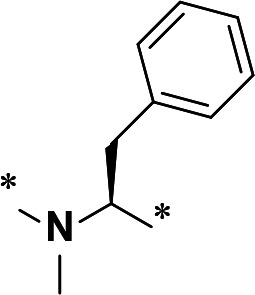	625.1 ± 10.5 nM	**H117**	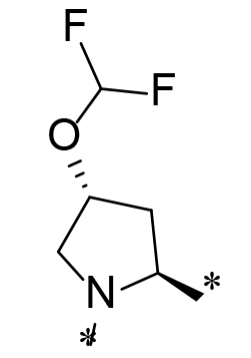	151.3 ± 5.2 nM
**H139**	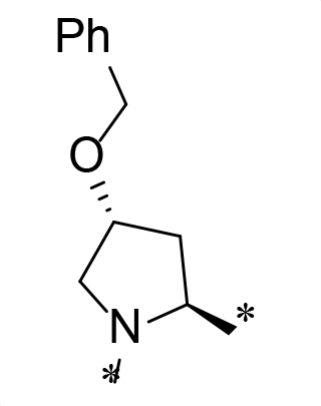	3.41 µM	**H118**	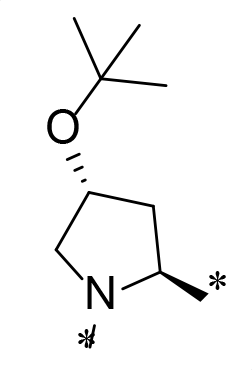	310.3 ± 18.9 nM
**H140**	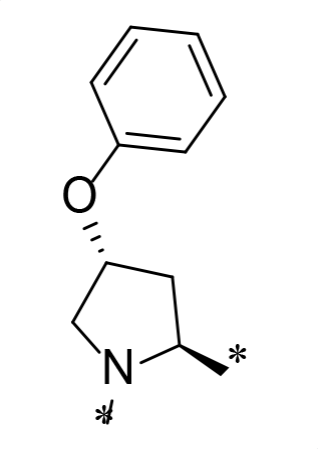	6.77 µM	**H121**	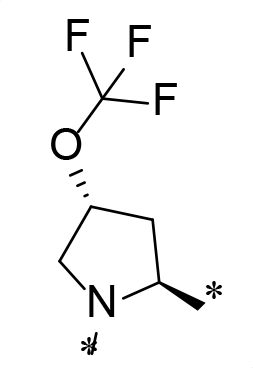	68.6 ± 2.0 nM
**H138**	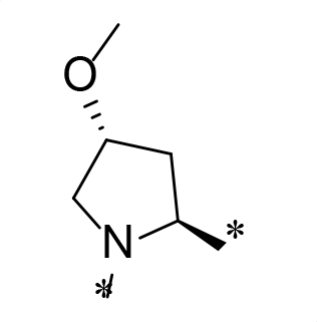	2.96 µM			

**TABLE 2:** M^pro^ inhibition of P3, Cap modified compounds

	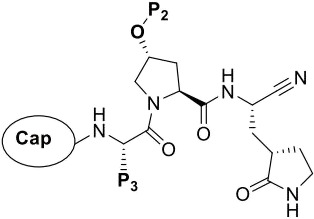			
Compound	Cap	P_3_	P_2_	M^pro^ IC_50_
**H130**	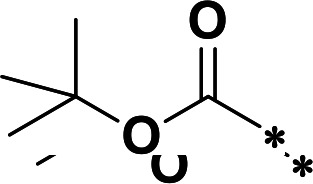	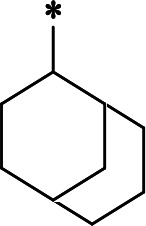	CHF_2_	70.1 ± 2.8 nM
**H131**	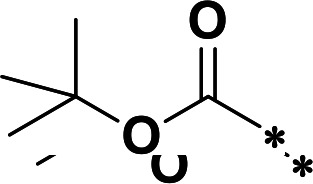	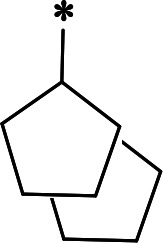	CHF_2_	79.2 ± 2.1 nM
**H141**	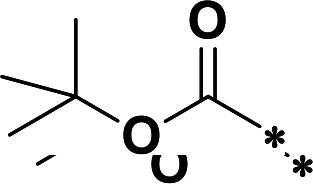	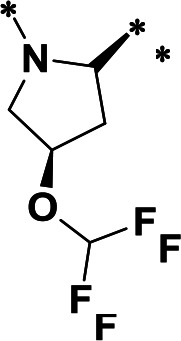	CHF_2_	>25 µM
**H122**	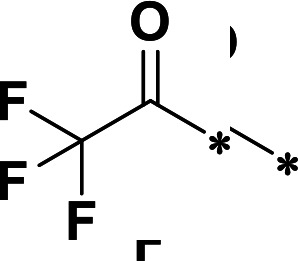	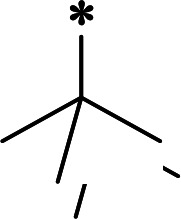	CHF_2_	105.7 ± 2.8 nM
**H123**	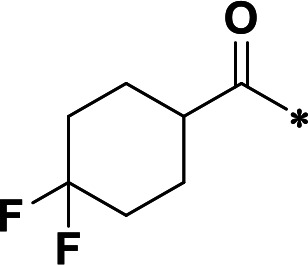	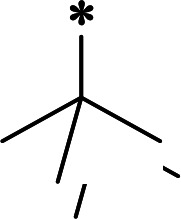	CHF_2_	860.9 ± 5.1 nM
**H124**	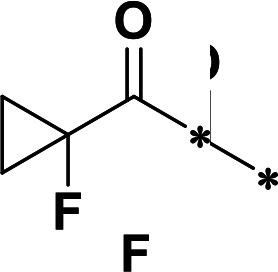	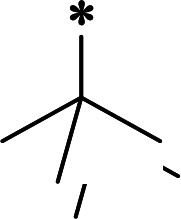	CHF_2_	101.7 ± 2.0 nM
**H129**	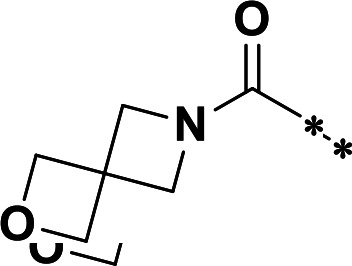	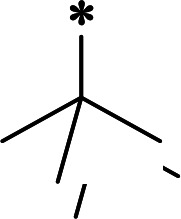	CHF_2_	924.1 nM
**H136**	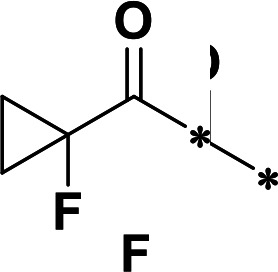	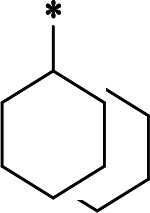	CHF_2_	43.4 ± 2.0 nM
**H135**	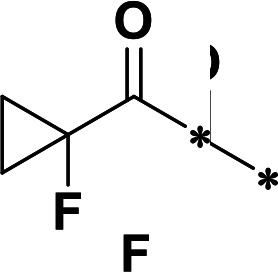	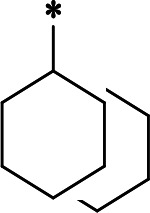	CF_3_	12.7 ± 0.1 nM
**PF-07321332**				22.2 ± 0.9 nM

In a previous study, we designed and synthesized two SARS-CoV-2 M^pro^ inhibitors, **H100** and **H102,** which share the same peptide backbone but have different cysteine-reactive warheads, ketoamide and aldehyde, respectively (17). **H100** and **H102** displayed potent M^pro^ inhibitory activities, with the IC_50_ values of 476.0 ± 11.6 nM and 8.8 ± 0.1 nM, respectively. In this present study, instead of the above-said warheads, we utilized a nitrile as a cysteine-reactive warhead and synthesized **H109** using the peptide backbone of **H100** and **H102** (Table 1). Even though **H109** exhibited M^pro^ inhibitory activity (IC_50_ = 407.0 ± 5.6 nM, Table 1) that was similar to **H100** and less than **H102**, **H109** exhibited more potent antiviral activity than both **H100** and **H102** (results not shown).

**Fig 2:**
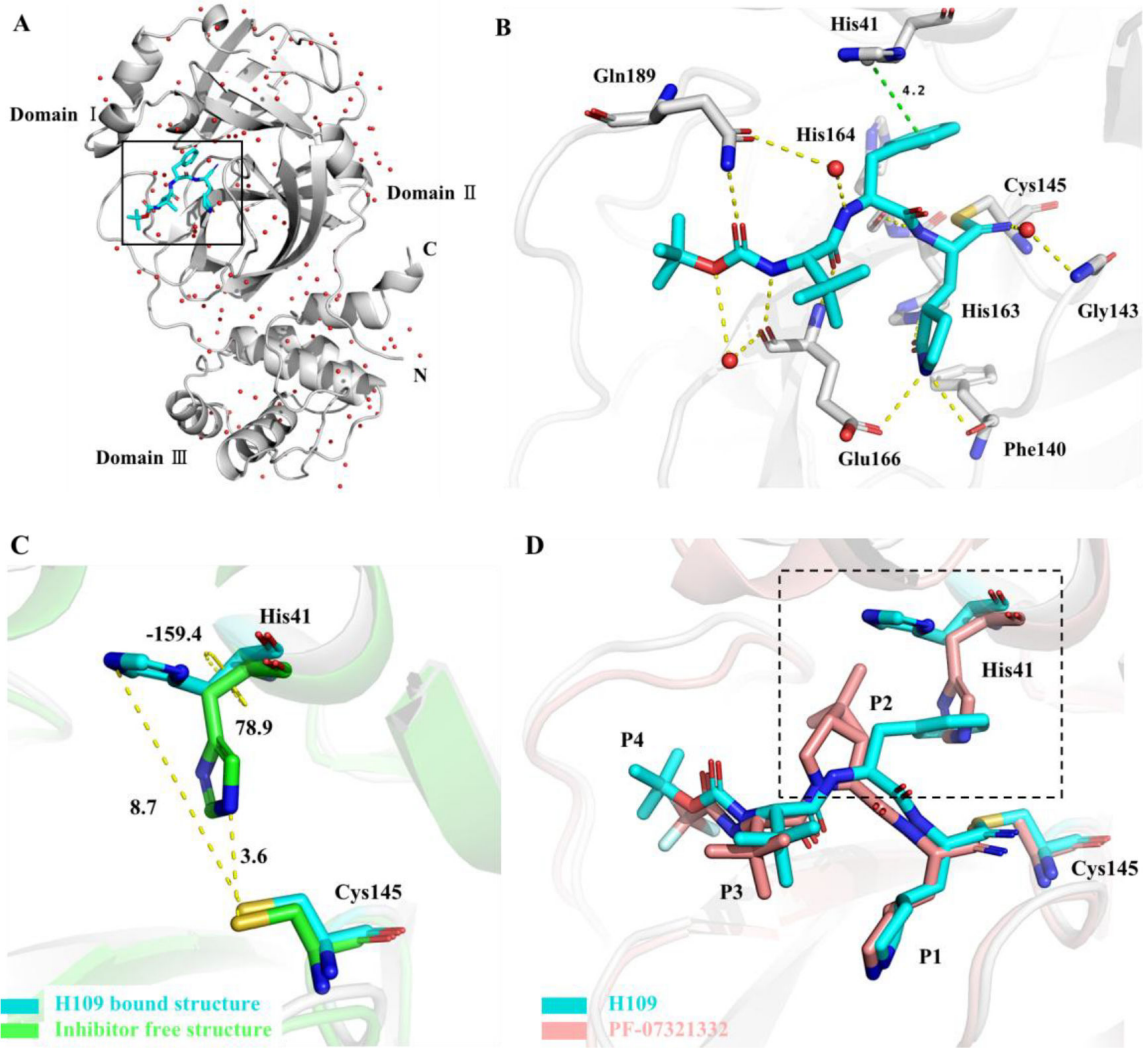
The co-crystal structure of SARS-CoV-2 M^pro^ in complex with compound **H109.** (**A**) Cartoon representation of the crystal structure of SARS-CoV-2 M^pro^ in complex with **H109. H109** is shown as cyan sticks; water molecules are shown as red spheres. (**B**) Close-up view of the **H109** binding pocket. (**C**) The distances between NE2-His41 and Sγ-Cys145 and the torsion angles NCαCβCγ(χ_1_) of His41 are shown before **H109** binding (green, PDB: 6Y2E) and after **H109** binding (cyan), highlighting the dramatic reorientation of His41 side chain due to **H109** binding. (**D**) Comparison of the binding modes of **H109** and PF-07321332 (PDB code: 7VH8) with SARS-CoV-2 M^pro^. The dashed square highlights the major differences at the P2 groups of the compounds and the imidazole group of His41 of M^pro^ between the **H109** and PF-07321332 bound structures.

To elucidate the structural mechanism of **H109** action, the co-crystal structure of **H109** bound to SARS-CoV-2 M^pro^ was determined at the resolution of 1.8 Å (Fig. 2A). As revealed in this co-crystal structure, the nitrile group of **H109** forms a C-S covalent bond with the Cys145 of SARS-CoV-2 M^pro^ (Fig. 2B). The nitrogen atom of the nitrile group also participates in stabilizing the conformation of the inhibitor by forming a hydrogen bond with oxyanion hole of residue Gly143 in the S1’ site through a water molecule. The *γ*-lactam group of **H109** forms three hydrogen bonds with the residues in the S1 pocket including Phe140, His163, and Glu166. The benzene ring of **H109** at its P2 position forms π-π interaction with the side chain of His41 of M^pro^ and causes His41 side chain to undergo a dramatic conformational change (Fig. 2C). The amide bonds of **H109** form multiple hydrogen bonds with the main chains of His164 and Glu166 and the side chain of Gln189 of M^pro^. In addition, two water molecules play an important role in **H109** binding to M^pro^. One water molecule helps form hydrogen bonds involving the P2 position’s NH group of **H109** and the side chain of Gln189 of M^pro^, while another water molecule contributes to hydrogen bond formation between the cap moiety of **H109** and the main chain of Glu166 of M^pro^.

In the inhibitor-free structure of SARS-CoV-2 M^pro^, His41 and Cys145 constitute the catalytic dyad with the imidazole group of His41 accepting a proton from Cys145 that activates the nucleophilic attack reaction (18). The distance between the NE2 atom of His41 and the Sγ atom of Cys145 is 3.6 Å in the inhibitor-free structure, whereas this distance changes to 8.7 Å upon **H109** binding (Fig. 2C). The torsion angle NCαCβCγ(χ_1_) of His41 is 78.9° (*gauche^-^*, g^-^) in the inhibitor-free structure, but changes to −159.4° (*trans*, t) upon **H109** binding. This suggests that the side chain of His41 in the inhibitor-free structure is in the least occupied conformational state, as the *g^-^* side chain conformation has a lower propensity (12.2%) compared to *g^+^* (54.6%) and *t* (33.2%) for the side chain conformation occurrence of His residues in proteins (19). The benzyl ring at the P2 position of **H109** is sandwiched between His41 and Cys145, which completely blocks the catalytic dyad interactions and provides a structural basis for the highly potent inhibitory activity of **H109**. While the overall interactions of **H109** with SARS-CoV-2 M^pro^ are similar to PF-07321332 (8), a major difference is shown in the orientation of the above-discussed benzyl ring at the P2 position of **H109** (Fig. 2D).

### Chemical modifications of H109 leading to a highly potent analog H135

With the activity and structural mechanism of **H109** characterized as described above, we continued to explore chemical modifications of this compound for further activity optimization. Our initial attention was on the P2 position of this compound, whose enzyme-bound structure was very different from that of other reported inhibitors, such as PF-07321332, as described above. To explore the effect on activity of restraining the conformation of the P2 position’s Phe residue seen in the **H109** co-crystal structure, we introduced *N*-methylation at P2 to yield analog **H119** (Table 1). **H119** displayed a 1.5-fold decrease in enzyme inhibitory activity.

Considering the hydrophobic and flexible character of the S2 pocket, we continued to explore the P2 position by introducing different substituents to proline analog *trans*-4-hydroxy-*L*-proline. Hydroxyproline is the most common proline analog found in clinical drugs and natural biochemical environment (20, 21). Proline, served as *N*-alkyl amino acid analogs, could restrict the conformation of the compound. The eliminated hydrogen bond donors improve the molecule’s metabolic stability and permeability. It is also a good chemical building block that enables versatile modification on its hydroxyl group. Introducing aromatic (phenyl and benzyl) substituents to a *trans*-4-hydroxy-*L*-proline to yield analogs **H139** and **H140**, both **H139** and **H140** showed drastically decreased activities (Table 1). In view of these results, instead of aromatic substituents, we then attempted other hydrophobic and varied size substituents on *trans*-4-hydroxy-*L*-proline (**H138, H116–118, H121**). Among these, the CF_3_-containing compound **H121** showed the best M^pro^ inhibition activity (68.6 ± 2.0 nM).

We next investigated modifications at the P3 and Cap positions of an M^pro^ inhibitor selected from the above-described P2 position-modified analogs. While **H121** showed the best enzyme inhibitory activity, we chose **H117** as the template for studying modifications of the P3 and Cap positions because **H117**’s chemical structure was more amenable to synthesis than **H121**. The cyclic alkyl substitutions were attempted at the P3 position of **H117**, yielding three analogs, such as **H130**, **H131**, and **H141** (Table 2). Among these, **H130** displayed the best activity. On the other hand, modifications on the Cap position of **H117** led to four different analogs, **H122–124** and **H129,** with **H124** showing the best activity. Combining the modifications of P3 and Cap positions from the best analogs **H130** and **H124**, respectively, we synthesized **H136** with combined activity improvement in M^pro^ inhibition (43.4 ± 2.0 nM).

**Fig 3:**
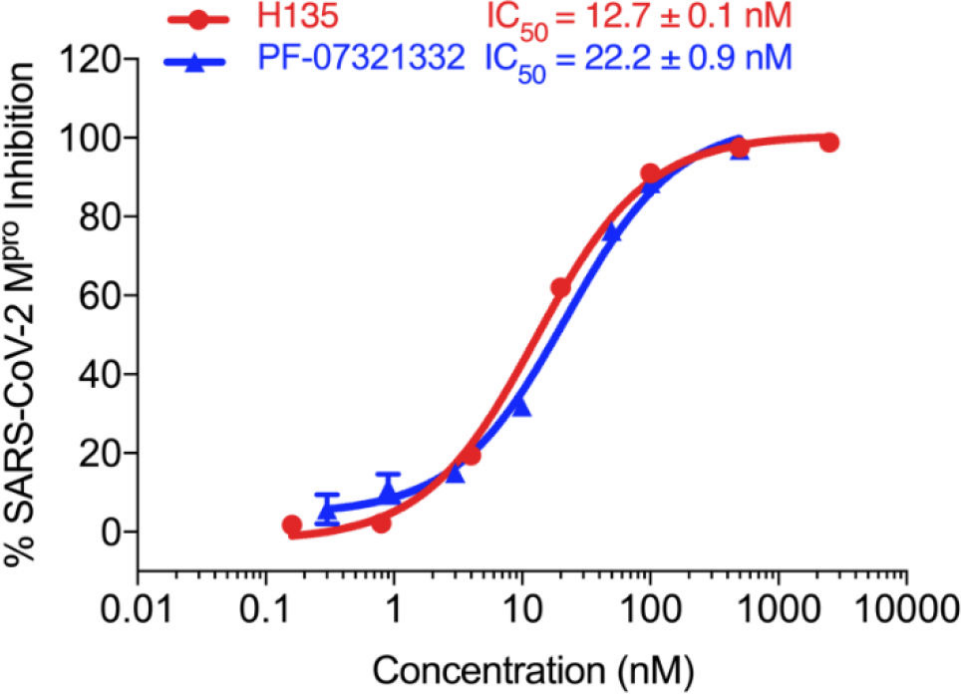
The SARS-CoV-2 M^pro^ enzyme inhibitory activity of **H135** and the control PF-07321332. All experiments were performed in triplicate. The data are shown as Mean ± SEM.

Finally, we incorporated the P2 modification in **H121**, which had the best activity of the P2 series into **H136** to obtain **H135**. Thus, **H135** contained the most effective modifications found for P2, P3, and Cap positions. The SARS-CoV-2 M^pro^ inhibition assay showed that **H135** displayed the most potent activity among all the analogs described here in this study. In comparative experiments with the positive control PF-07321332 (nirmatrelvir), **H135** exhibited higher potency (12.7 vs. 22.2 nM) and inhibitory constant (Ki, 156.2 nM vs. 191.2 nM) (Fig. 3). In further specificity studies, **H135** showed no inhibitory effect against several common human host cysteine proteases, including cathepsin B, cathepsin L, and caspase 2 (IC_50_ >100 µM, Table 3). These data suggest that **H135** is a selective M^pro^ inhibitor.

**TABLE 3:** Selectivity of H135 against several common human host cysteine proteases

Protease	IC_50_ (μM)
Cathepsin B	>100
Cathepsin L	>100
Caspase 2	>100

### *In vitro* antiviral activity of H135

**Fig 4:**
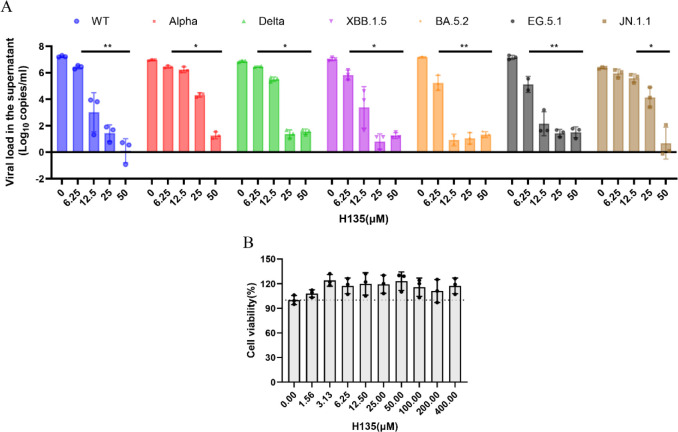
*In vitro* antiviral activity of **H135**. (**A**) VeroE6-TMPRSS2 cells were infected with different SARS-CoV-2 strains at 0.01 MOI and treated with indicated concentrations of **H135**. Viral yield in the cell supernatant was quantified at 48 hpi by qRT-PCR methods. Statistical analysis was performed using two-way ANOVA. *P < 0.05, **P < 0.01 when compared with the 0 µM groups. (**B**) Cytotoxicity of **H135** in VeroE6-TMPRSS2 cells was measured by CellTiter-Glo assay for 48 h.

Next, we prioritized **H135** for downstream antiviral characterization and employed the viral load reduction assay to evaluate its protection against a panel of SARS-CoV-2 variants. As shown in Fig. 4, **H135** exerts broad anti-SARS-CoV-2 activity in VeroE6-TMPRSS2 cells. Utilizing 50 µM **H135**, we demonstrated that viral yields in the cell culture supernatants were decreased by ~7-log_10_ in wild-type–infected cells, and by more than 5-log_10_ in Alpha, Delta, XBB.1.5, BA.5.2, EG.5.1, or JN.1.1 infected cell cultures (Fig. 4A). Importantly, no cytotoxicity was found at doses up to 400 µM in **H135**-treated VeroE6-TMPRSS2 cells for 48 h (Fig. 4B). Moreover, H135 exhibited an antiviral EC_50_ of 122 ± 13 nM in human lung Calu-3 cells. This result warrants further evaluation of **H135** in animal models of COVID-19.

### *In vivo* antiviral activity of H135 in animal models

**Fig 5:**
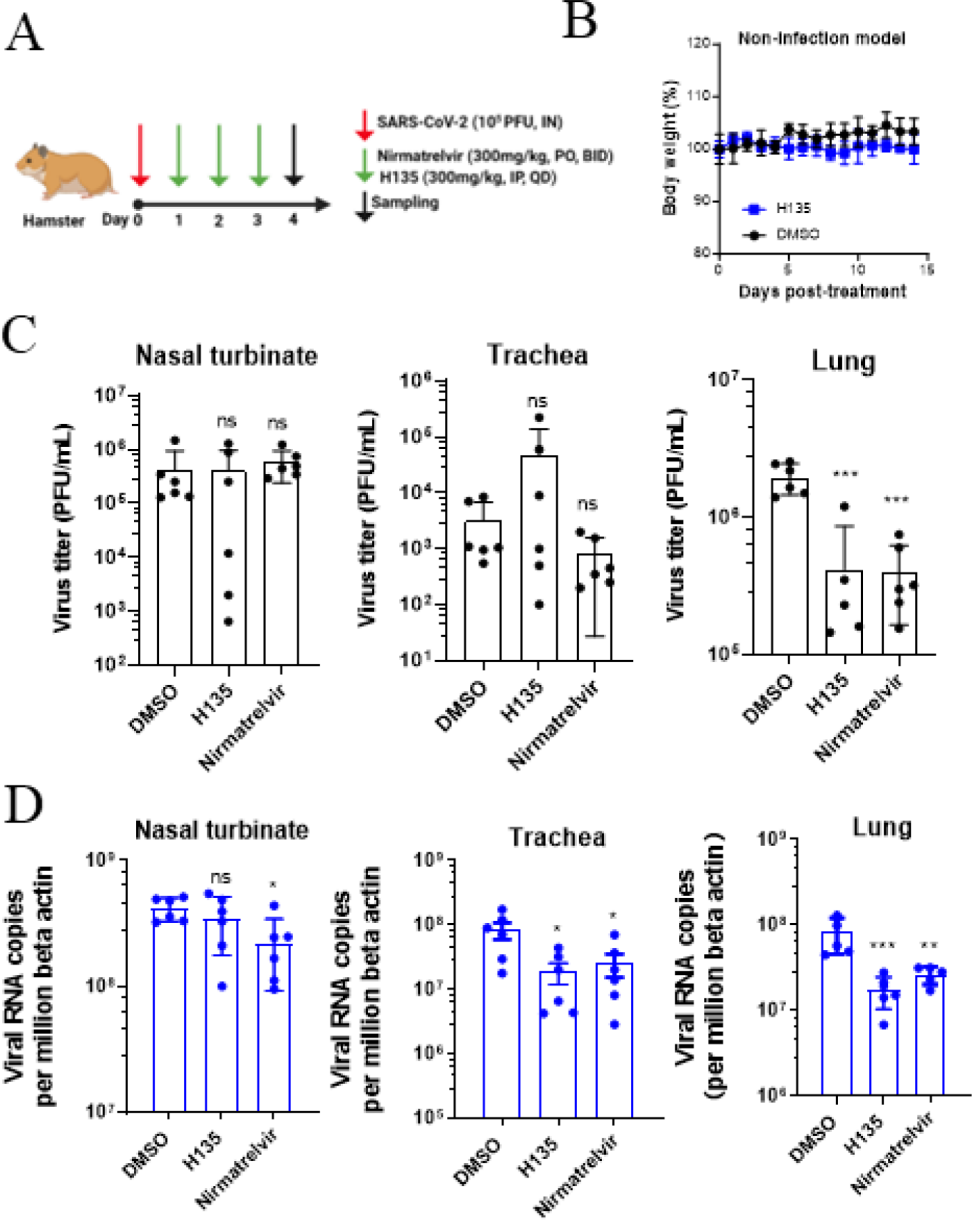
*In vivo* antiviral activity of **H135** in the golden Syrian hamster model. (**A**) Hamsters (*n* = 6) were intranasally inoculated with 10^5^ PFU of SARS-CoV-2 and given either DMSO (vehicle control, intraperitoneal), **H135** (intraperitoneal), and nirmatrelvir (oral) at the indicated dosage for three consecutive days. (**B**) To evaluate the toxicity of **H135** treatment, body weight of hamsters was recorded for 14 days in the absence of virus infection. At 4 dpi, respiratory tissue viral yields in the nasal turbinate, trachea, and lungs of the hamsters were determined by plaque assay (**C**) and RT-qPCR assay (**D**), respectively. All data are shown as Mean ± SD. Statistical significance was assessed by one-way ANOVA compared with DMSO group. *P < 0.05, **P < 0.01, and ***P < 0.001; ns, not significant.

We evaluated the *in vivo* antiviral effect of **H135** in the established golden Syrian hamster and K18-hACES mouse models (22–25). In the non-lethal Syrian hamster model, we examined whether **H135** conferred protection against SARS-CoV-2 infection by reducing the viral loads in the lower (lung, trachea) and upper (nasal turbinate) respiratory tract tissues (Fig. 5A). Negligible toxicity of **H135** (300 mg/kg/day for three days) was recorded as reflected by the animal body weight change (Fig. 5B). Apparently, both **H135** and the positive control inhibitor nirmatrelvir significantly reduced live virus titer in hamster lungs (*P* < 0.001, Fig. 5C). Decreased viral RNA copies in both the lung (*P* < 0.001) and trachea (*P* < 0.05) by ~0.7 log and ~0.65 log were also recorded when compared with that of the DMSO-treated hamsters (Fig. 5D). In general, the *in vivo* efficacy between 300 mg/kg nirmatrelvir (300 mg/kg, PO and BID) was comparable to that of **H135** (300 mg/kg, IP and QD).

**Fig 6:**
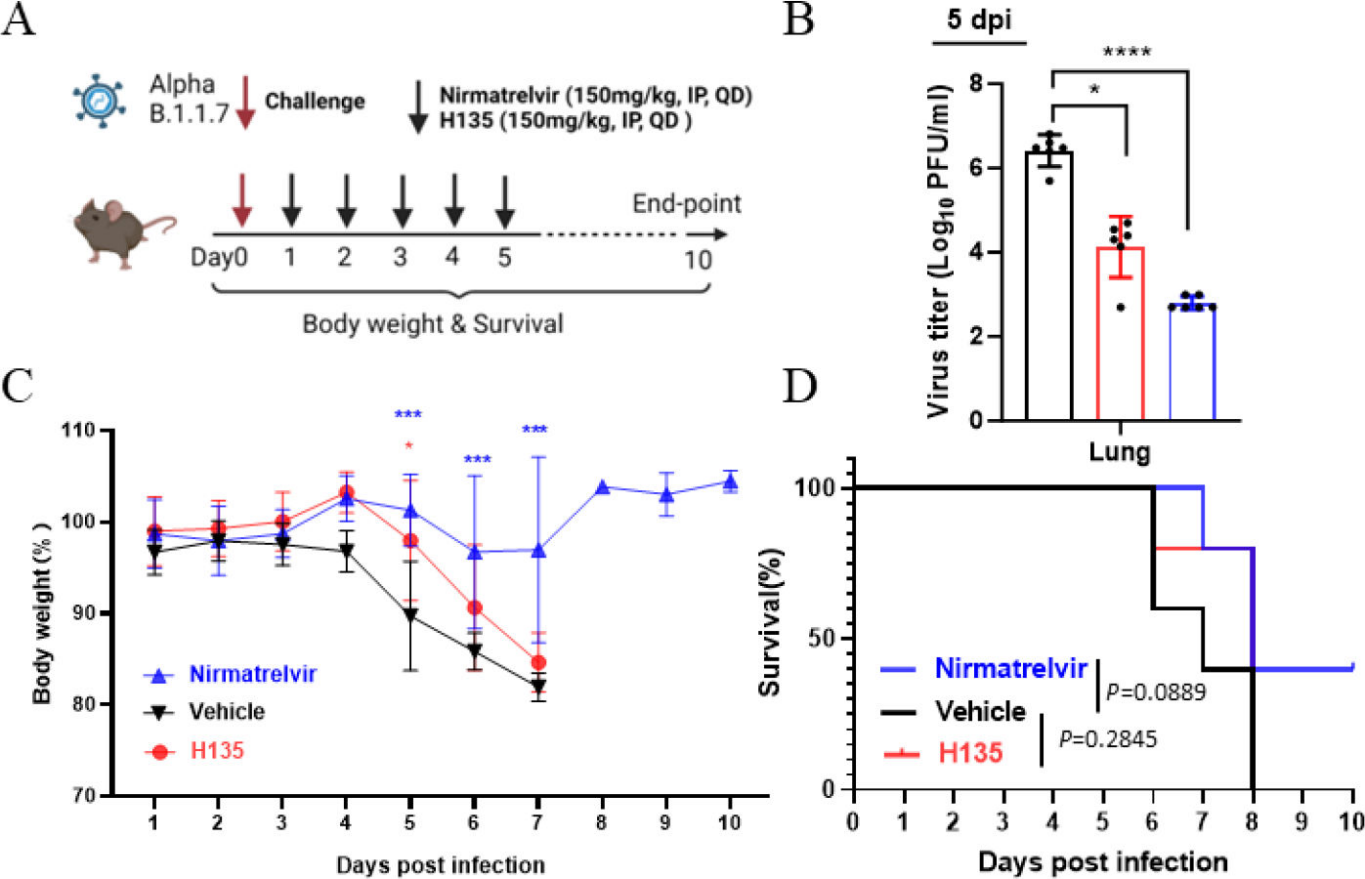
*In vivo* antiviral activity of **H135** in a K18-hACE2 mouse model. (**A**) Mice (*n* = 10) were intranasally inoculated with lethal dose of SARS-CoV-2 Alpha strain (B.1.1.7, 200 PFU) and given either DMSO, **H135,** and nirmatrelvir at the indicated dosage via intraperitoneal route. (**B**) Five mice in each group were sacrificed for virus titration in the lungs. Their body weight (**C**) and (**D**) survival rate were monitored daily for 10 days or until death. *P < 0.05, ***P < 0.001, ****P < 0.0001.

To further investigate the efficacy of **H135** *in vivo* in a lethal animal model, we monitored the body weight and survival rate of SARS-CoV-2-infected K18-hACE2 mice (Fig. 6A). As shown in Fig. 6B, intraperitoneal injection of **H135** reduced SARS-CoV-2 live virus titers by more than 2 logs_10_ in the mouse lung and at 5 dpi. Nirmatrelvir protected 40% mice from death, with body weight benefits observed at 5, 6, and 7 dpi (Fig. 6C and D). The fatality rate was still 100% after **H135** treatment, whereas delayed death by 0.6 day/mouse and less body weight loss at 5 dpi (*P* < 0.05) were documented in the **H135** group (Fig. 6). These results indicate further profiling of **H135**’s ADME in the future.

### Conclusion

In this study, a structure-guided hit-to-lead strategy was employed to develop a nanomolar potent small molecule inhibitor **H135** of SARS-CoV-2 M^pro^ with strong anti-SARS-CoV-2 infection activity in cells and animals. Starting from the high-resolution co-crystal structure of SARS-CoV-2 M^pro^ bound by a hit compound **H109** containing a nitrile warhead, chemical modifications were made to its P2, P3, and Cap position to derive a final lead compound **H135** with SARS-CoV-2 M^pro^ inhibitory potency enhanced by 32 folds. **H135** is a selective M^pro^ inhibitor and exhibited higher potency (12.7 nM) than the clinically approved SARS-CoV-2 M^pro^ drug PF-07321332 (22.2 nM). **H135** exerted broad anti-SARS-CoV-2 activity in VeroE6-TMPRSS2 cells. Most importantly, in a golden Syrian hamster model, **H135** conferred protection against SARS-CoV-2 infection by reducing the viral loads in the lower (lung and trachea) respiratory tract tissues. **H135** may serve as a new lead for developing antiviral agents targeting the virus’s main protease M^pro^.

## MATERIALS AND METHODS

### Compound synthesis and characterization

The details of synthetic methods for preparing target compounds, as well as ^1^H NMR and HRMS spectra of intermediates and target compounds, are provided in Supplementary information.

### Viruses and safety

WT SARS-CoV-2 HKU-001a (GenBank: MT230904), B.1.1.7/Alpha (GenBank: OM212469), B.1.351/Beta (GenBank: OM212470), B.1.617.2/Delta (GenBank: OM212471), Omicron BA.5.2 (GISAID: EPI_ISL_13777659), JN.1.1 (GISAID: EPL_ISL_18841631), XBB.1.5 (GISAID: EPI_ISL_15602393), and EG.5.1 (GISAID: EPI_ISL_18461518) were archived in the Department of Microbiology, the University of Hong Kong (HKU). All variants of SARS-CoV-2 were cultured and titrated by plaque assays using VeroE6-TMPRSS2 cells. After obtaining the virus culture, the sequences of all variants used in this study were confirmed with nanopore sequencing. *In vivo* and *in vitro* experiments with infectious SARS-CoV-2 were performed according to the approved standard operating procedures of the Biosafety Level 3 facility at the Department of Microbiology, HKU (26).

### Protein expression and purification of SARS-CoV-2 M^pro^

The pET-28b-SARS-CoV-2 M^pro^ plasmid was transformed into *E. coli* strain BL21(DE3) cells and then cultured in LB medium containing 50 µg/mL kanamycin in a shaking incubator at 37°C. When the cells were grown to an OD_600_ of 0.6–0.8, 0.6 mM IPTG was added to the cell culture to induce the protein expression at 16°C. After 18 h, the cells were harvested by centrifugation at 4,000 rpm for 20 min at 4°C. The cell pellets were washed twice by PBS, resuspended in lysis buffer (50 mM HEPES, 300 mM NaCl, 10 mM imidazole, pH 7.5), and lysed by sonication on ice for 3 seconds ON time 5 seconds OFF time for 30 min of total time and then clarified by ultracentrifugation at 18,000 rpm at 4°C for 40 min to remove debris. The supernatants were then purified by TALON metal affinity resin (TaKaRa, 635501) and washed with washing buffer (25 mM HEPES, 500 mM NaCl, pH 7.5) to remove unspecific binding proteins. The SUMO-His-tagged SARS-CoV-2 M^pro^ was eluted by elution buffer (25 mM HEPES, 500 mM NaCl, 300 mM imidazole, pH7.5). The SUMO-His-tagged SARS-CoV-2 M^pro^ was then treated overnight at 4℃ with His-tagged SUMO protease (home-made) to remove the SUMO-His-tag. The SARS-CoV-2 M^pro^ was further purified by His60 Ni superflow resin (TaKaRa, 635659). The quality of SARS-CoV-2 M^pro^ was checked by Coomassie-stained SDS-PAGE gel. The concentration was determined via BCA Protein Assay Kit. The purified SARS-CoV-2 M^pro^ was stored in a buffer containing 25 mM HEPES, 1 mM DTT, 1 mM EDTA, and 10% glycerol (pH 7.5) (27, 28).

### Crystallization of SARS-CoV-2 M^pro^ in complex with H109

Concentrations of 5 mg/mL and 10 mg/mL M^pro^ (in a solution containing 20 mM Tris, 150 mM NaCl, 1 mM EDTA, and 1 mM TCEP [pH 7.8]) were incubated with 10 mM H109 at 1:10 vol ratio at room temperature for 2 h. The crystals were obtained by using the sitting-drop vapor diffusion method by mixing 1 µL of protein with 1 µL of reservoir solution, then equilibrating the mixture against 100 µL of the reservoir solution at 18°C. The initial crystallization screenings were carried out using commercially available kits. The complexes were crystallized in a solution containing 0.1 M MES monohydrate (pH 6.0) and 20% (wt/vol) polyethylene glycol monomethyl ether 2000.

### Data collection and structure determination

Diffraction data were collected at the Shanghai Synchrotron Radiation Facility (SSRF) BL17U (wavelength, 0.97918 Å). For data collection, the crystals were cryo-protected by briefly soaking in reservoir solution supplemented with 20% (vol/vol) glycerol before flash-cooling in liquid nitrogen. The data set was processed with HKL2000 software. The structure was determined by the molecular replacement method using Phaser with the previously reported structure (PDB: 7C6S). The atomic models were completed with Coot and refined with phenix.refine in Phenix, and the stereochemical qualities of the final models were assessed using MolProbity. Data collection, processing, and refinement statistics are summarized in Table S1 (see Supplementary Information).

### SARS-CoV-2 M^pro^ enzyme inhibition assay

The enzyme inhibition assay was carried out in assay reaction buffer (25 mM HEPES, 1 mM DTT, 1 mM EDTA, 0.01% Triton X-100, pH 7.5) by pre-incubating SARS-CoV-2 M^pro^ at a final concentration of 150 nM with compounds at various concentrations at 37°C with gentle shaking for 30 min in blank 96-well plates. Thereafter, the reaction was initiated by adding 25 µM M^pro^ fluorogenic substrate (Dabcyl-KTSAVLQSGFRKME-Edans) for 1 h. The relative fluorescence units (RFU) were measured by a PerkinElmer EnVision multimode plate reader with an excitation wavelength of 340 nm and an emission wavelength of 490 nm. Percent inhibition was calculated based on control wells containing no compound (100% activity) and a blank control. The IC_50_ values were calculated using GraphPad Prism software. All experiments were performed in triplicate, and the values are presented as mean ± SEM.

### SARS-CoV-2 viral load reduction assay

Viral load reduction assay was performed for the evaluation of antiviral activity as we described previously (24, 29). Briefly, SARS-CoV-2-infected VeroE6 cells were treated with different concentrations of compounds or dimethyl sulfoxide (DMSO) control. Then, cell culture supernatant samples were collected at 48 h post-inoculation (hpi) for qRT-PCR analysis of viral RNA load. Culture supernatant was lysed with buffer AVL and then extracted for total RNA with the QIAamp viral RNA mini kit (Qiagen). qRT-PCR was used for quantitation of SARS-CoV-2 viral load using the QuantiNova Probe RT-PCR kit (Qiagen) with a LightCycler 480 Real-Time PCR System (Roche). Each 20 µL reaction mixture contained 10 µL of 2 × QuantiNova Probe RT-PCR Master Mix, 1.2 µL of RNase-free water, 0.2 µL of QuantiNova Probe RT-Mix, 1.6 µL each of 10 µM forward and reverse primers, 0.4 µL of 10 µM probe, and 5 µL of extracted RNA as the template. Reactions were incubated at 45°C for 10 min for reverse transcription, 95°C for 5 min for denaturation, followed by 45 cycles of 95°C for 5 s and 55°C for 30 s. Signal detection and measurement were taken in each cycle after the annealing step. The cycling profile ended with a cooling step at 40°C for 30 s. The primers and probe sequences were against the RNA-dependent RNA polymerase/Helicase (RdRP/Hel) gene region of SARS-CoV-2: forward primer: 5′-CGCATACAGTCTTRCAGGCT-3′; reverse primer: 5′-GTGTGATGTTGAWATGACATGGTC-3′; specific probe: 5′-FAMTTAAGATGTGGTGCTTGCATACGTAGAC-IABkFQ-3′. The viral load reduction assay experiments were performed in triplicate.

### Selectivity assay against human host cysteine proteases

The biochemical activity against several common human cysteine proteases was assessed using Cathepsin B inhibitor screening kit (BioVision, K147-100), Cathepsin L inhibitor screening kit (BioVision, K161-100), and Caspase 2 inhibitor drug screening kit (BioVision, K152-100) following the manufacturer’s instructions.

### *In vivo* antiviral evaluation in the golden Syrian hamster model

All experimental protocols involving animal experiments in this study were approved by the Committee on the Use of Live Animals in Teaching and Research (CULATR) and were performed according to the standard operating procedures of the Biosafety Level 3 laboratory at the University of Hong Kong (HKU). Male Syrian hamsters, aged 6–10 weeks, were kept given access to standard pellet feed and water *ad libitum* until virus challenge as previously described (24). The hamsters were randomly allocated to different groups (*n* = 6) for antiviral evaluation. All experimental protocols were approved by the Committee on the Use of Live Animals in Teaching and Research (CULATR) and were performed according to the standard operating procedures of the Biosafety Level 3 laboratory at the University of Hong Kong (HKU). The experiments were not blinded. Each hamster was intranasally inoculated with 10^5^ PFU of SARS-CoV-2 in 100 µL PBS under intraperitoneal ketamine (200 mg per kg body weight) and xylazine (10 mg per kg body weight) anesthesia. At one day post-virus challenge, each hamster was intraperitoneally given **H135** (300 mg per kg body weight per day), orally given nirmatrelvir (300 mg per kg body weight with twice per day), or DMSO (vehicle controls) for three consecutive days. The animals were monitored twice daily for clinical signs of disease. Animals in each group were euthanized at 4 dpi for virological analyses. Lung, trachea, and nasal turbinate specimens were collected at sacrifice. Viral titer and viral load in the tissue homogenates were detected by plaque assay and qRT–PCR methods, respectively.

### *In vivo* antiviral evaluation in the K18-hACE2 mouse model

The K18-hACE2 mice, 6 to 8 weeks old, were obtained from the HKU Center for Comparative Medicine Research, as previously described (25). Anesthetized K18-hACE2 mice (intraperitoneal ketamine and xylazine) were infected at 200 PFU of SARS-CoV-2 diluted in PBS by intranasal injection. Each mouse was dosed with **H135** (150 mg/kg, dissolved in ddH2O with 3% DMSO, 13.5% Cremopher EL, and 11.5% PEG400), nirmatrelvir (150 mg/kg/day, dissolved in ddH2O with 0.5% methylcellulose and 2% Tween 80), or vehicle (3% DMSO, 13.5% Cremopher EL, and 11.5% PEG400 in ddH_2_O) daily by intraperitoneal injection.

## Data Availability

The coordinates and structure factors have been deposited in the PDB with accession code 9IK2. Additional data are provided in supplemental material. Source data are provided with this paper.

## SUPPLEMENTAL MATERIAL

Supplemental material jvi.01001-25-s0001.pdf Supplemental methods and characterization of the target compound.

ASM does not own the copyrights to Supplemental Material that may be linked to, or accessed through, an article. The authors have granted ASM a non-exclusive, world-wide license to publish the Supplemental Material files. Please contact the corresponding author directly for reuse.
