# Supplementary material for: Structure-guided discovery of a small molecule inhibitor of SARS-CoV-2 main protease with potent in vitro and in vivo antiviral activities
Source: J Virol. 2025 Nov 14;99(12):e01001-25. doi: 10.1128/jvi.01001-25 (PMC12724327; doi:10.1128/jvi.01001-25)
Supplement: Supplemental material — Supplemental methods and characterization of the target compound. [file jvi.01001-25-s0001.pdf]

Supplementary Information For

Structure-guided discovery of a small molecule inhibitor of SARS-CoV-2 main protease with potent *in vitro* and *in vivo* antiviral activities

Table of Contents

I. Supplementary Methods..2
II. ^1H NMR, ^{13}C NMR, HRMS, HPLC of target compound.....................27
III. Supplementary References...45

Supplementary Methods

Synthesis of M^{pro} Inhibitors

The synthetic routes for nitrile-based target compounds outlined in supplementary Scheme 1-4. The intermediate **3** was prepared according to literature[1]. Amidation of **1** provided compound **2**. The Boc group of compound **2** was removed using TFA to give intermediate **3**.

The preparation of target compounds **H109**, **H119**, **H116** was depicted in supplementary Scheme 1. Boc-Tle-OH **4** coupled with corresponding hydrochloride salt of amino acid to give **5a-c**, which were subsequently hydrolyzed to give **6a-c**. The obtained **6a-c** coupled with compound **3** yielded **7a-c**, followed by treatment with Burgess reagent, yielded **H109**, **H119**, **H116**.

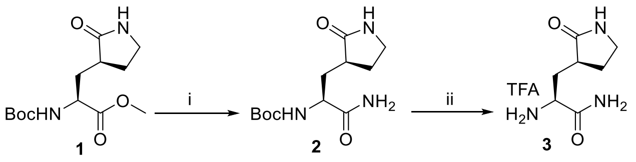

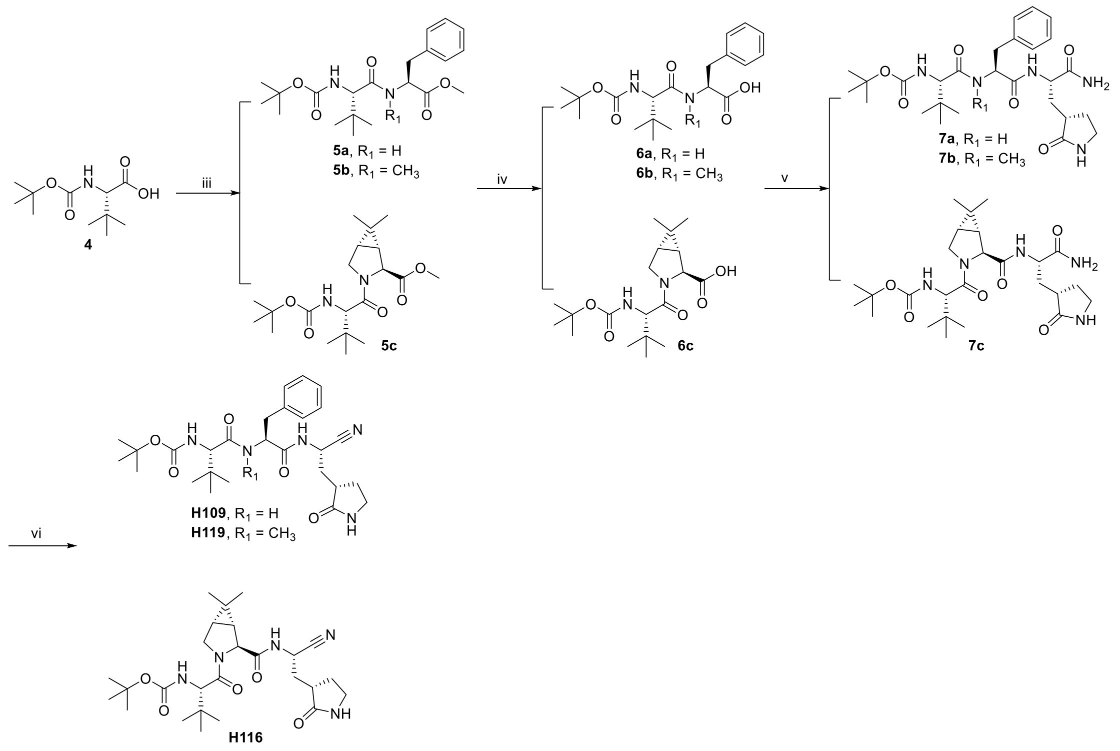

Supplementary Scheme 1. Synthesis of **H109**, **H116** and **H119**[a]

[a]Reagents and conditions: (i) 7M NH_3 in CH_3OH, rt, 2 days; (ii) TFA, CH_2Cl_2, 0 °C→ rt, 3 h; (iii) HATU, DIEA, DMF, ACN, 0 °C → rt, 4 h; (iv) $LiOH.H_2O$, THF, H_2O, 0 °C → rt, 3 h; (v) EDCI·HCl, HOPO, DIEA, MEK, 0 °C→ rt, 12h; (vi) Burgess reagent, CH_2Cl_2, rt, 1 h.

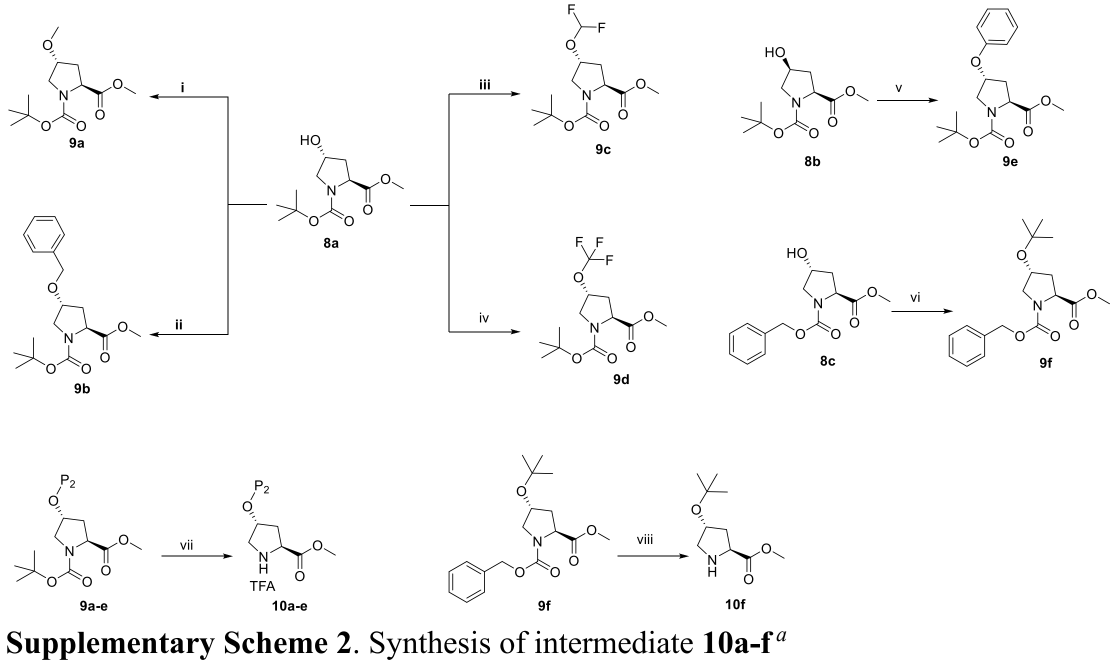

Supplementary Scheme 2. Synthesis of intermediate **10a-f** [a]

[a]Reagents and conditions: (i) NaH, CH_3I, DMF, 0°C, 1h; (ii) $PhCH_2Br$, NaH, THF, 0°C→rt, 5h; (iii) $FSO_2CF_2CO_2H$, CuI, CH_3CN, 50 °C, 1h; (iv) $TMSCF_3$, KF, AgOTf,

2-fluoropyridine, Selectfluor, EtOAc, rt, 14h. (v) PhOH, PPh_3, DEAD, THF, 0 °C→ rt, 12h; (vi) 2,6-lutidine, TBTA, PhF, bis(trifluoromethane)sulfonamide, rt, 14h; (vii) TFA, CH_2Cl_2, 0 °C→rt, 3 h; (viii) Pd/C, H_2, CH_3OH, rt, overhight.

9a-f were synthesized as reported in the literature. Starting material *N*-Boc-*trans*-4-hydroxy-*L*-proline methyl ester **8a** underwent substitution reaction with the iodomethane and benzyl bromide to give **9a** and **9b** at 90% and 89% yield respectively[2]. O-difluoromethylation of **8a** under CuI catalysis to afford **9c** (72% yield)[3]. AgOTf-mediated oxidative O-trifluoromethylation of **8a** with $TMSCF_3$ to give **9d** (27% yield)[4]. **8b** underwent mitsunobu reaction with PhOH led to **9e** (63% yield)[5]. Tert-butylation of **8c** using 2,6-lutidine and bis(trifluoromethane)sulfonamide to give **9f** (93% yield) [6]**.** Boc and Cbz deprotection to give **10a-f**.

With unnatural amino acid **10a-f** in hand, **H117-H118**, **H121-H124**, **H138-H140** were prepared. The synthetic procedures for compounds were similar with compound **H119** (supplementary Scheme 3).

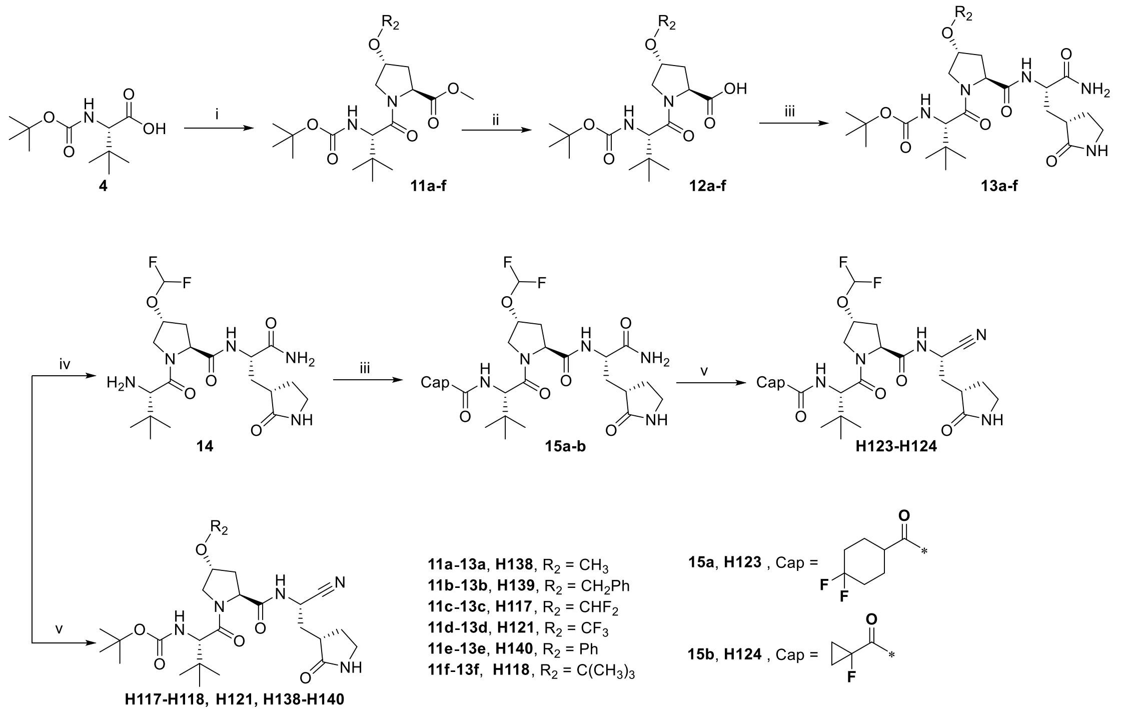

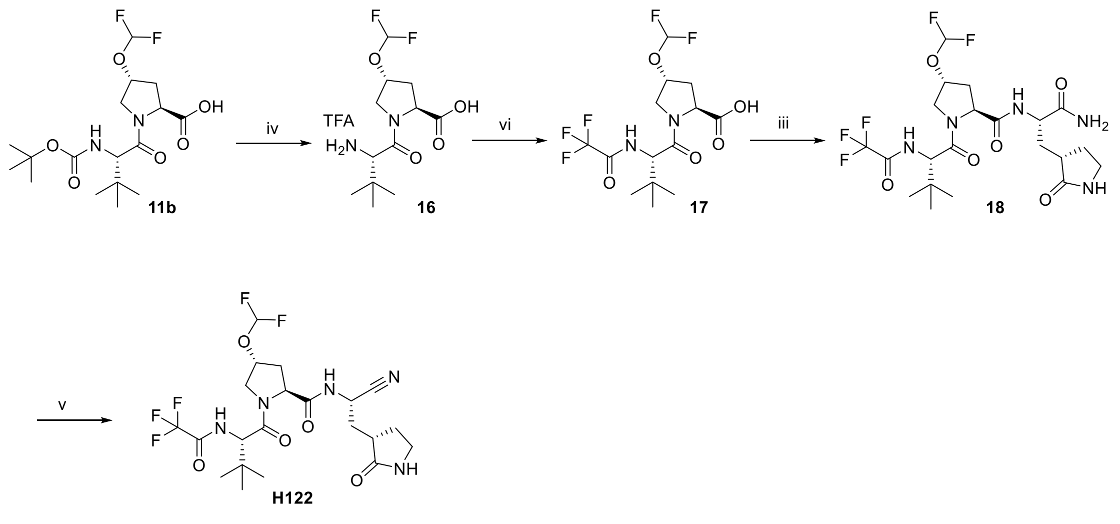

Supplementary Scheme 3. Synthesis of **H117-H118**, **H121-H124**, **H138-H140** [a]
[a]Reagents and conditions: (i) **10a-f**, HATU, DIEA, DMF, ACN, 0 °C→ rt, 4 h; (ii) LiOH·H_2O, THF, H_2O, 0 °C→ rt, 3 h; (iii) EDCI·HCl, HOPO, DIEA, MEK, 0 °C→ rt, 12h; (iv) TFA, CH_2Cl_2, 0 °C→rt, 3 h; (v) Burgess reagent, CH_2Cl_2, rt, 1 h; (vi) trifluoroacetate, TEA, CH_3OH, 0 °C→rt

L-*tert*-Leucine methyl ester **23** reacted with 2-oxa-6-azaspiro[3.3]heptane in the presence of CDI to give **24**. *L*-cyclohexylglycine methyl ester **26** coupled with 1-fluoro-cyclopropanecarboxylic acid to give **27**. **24** and **27** hydrolyzed to give **26** and **28.** The synthesis of **H129-H131**, **H135**, **H136, H141** followed a similar route with **H119** (supplementary Scheme 4).

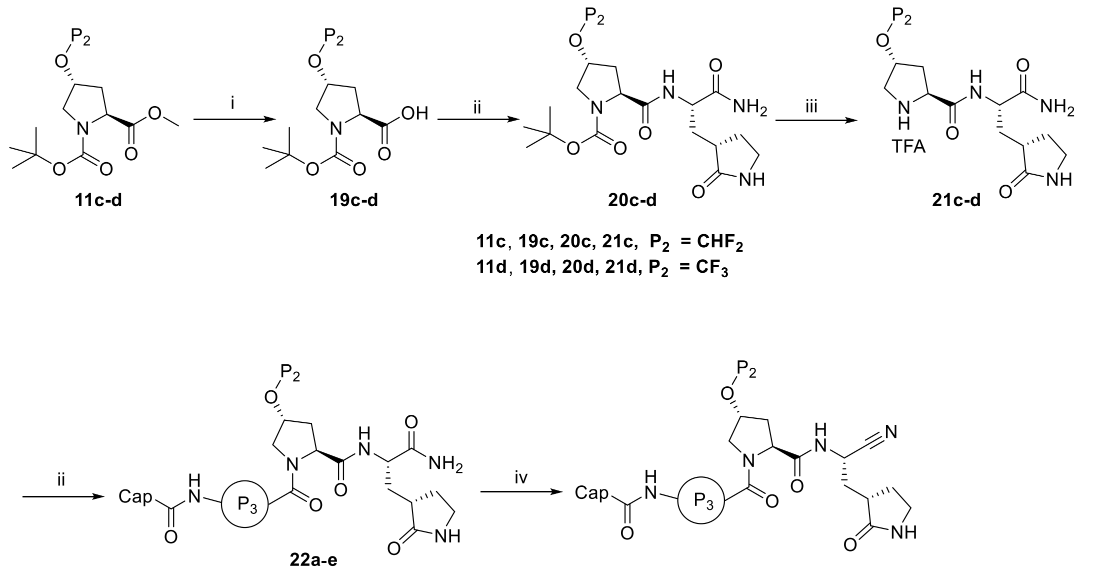

22a, H129, Cap = 2-oxa-6-azaspiro[3.3]heptan-6-yl (structure) P_3 = tBu, P_2 = CHF_2
22b, H130, Cap = $(CH_3)_3O$, P_3 = cyclohexanyl, P_2 = CHF_2
22c, H131, Cap = $(CH_3)_3O$, P_3 = cyclopentyl, P_2 = CHF_2
22d, H135, Cap = 1-fluoro-cyclopropanyl, P_3 = cyclohexanyl, P_2 = CF_3
22e, H136, Cap = 1-fluoro-cyclopropanyl, P_3 = cyclohexanyl, P_2 = CHF_2
22f, H141, Cap = $(CH_3)_3O$, P_3 = *trans*-4-difluoromethoxy-*L*-proline, P_2 = CHF_2

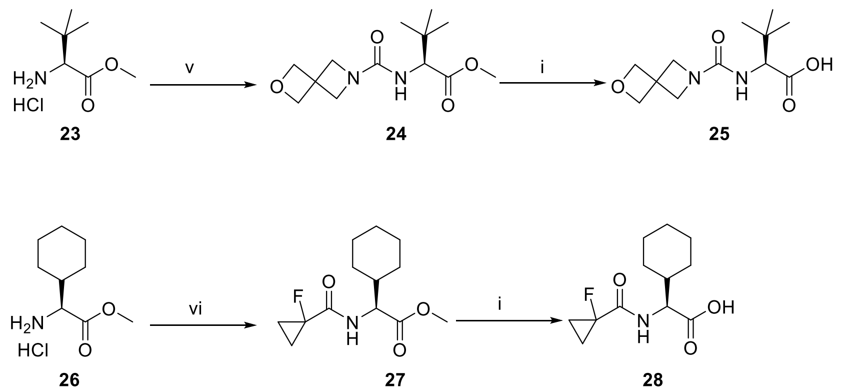

Supplementary Scheme 4. Synthesis of **H129-H131**, **H135**, **H136, H141**[a]
[a]Reagents and conditions: (i) LiOH.H_2O, THF, H_2O, 0 °C→ rt, 3.5 h; (ii) EDCI·HCl, HOPO, DIEA, MEK, 0 °C→ rt, 12h; (iii) TFA, CH_2Cl_2, 0 °C→rt, 3 h; (iv) Burgess reagent, CH_2Cl_2, rt, 1 h; (v) CDI, TEA, ACN, 0 °C→ rt, 5 h; (vi) HATU, DIEA, DMF, ACN, 0 °C→ rt, 4 h.

Experimental Details

Commercially available reagents were used directly without further purification. The high-resolution MS (HRMS) of target compounds were analyzed by using Waters Xevo G2 QTof in positive ion mode. ^{1}H NMR spectra were obtained on the Bruker Ascend™ 400. The chemical shifts (δ) are reported in parts per million (ppm) using suitable deuterated NMR solvents relative to tetramethyl silane (TMS) at 0 ppm. Multiplicities are defined as follows: s (singlet), brs (broad singlet), d (doublet), t (triplet), q (quartet), dd (doublet of doublets), and m (multiplets). The reaction was monitored by TLC (silica gel GF254). The compounds were purified by silica chromatography (silica gel 100-200 mesh or 200-300 mesh) or C18 reverse-phase preparative HPLC.

***Tert*-butyl ((*S*)-1-amino-1-oxo-3-((*S*)-2-oxopyrrolidin-3-yl)propan-2-yl)carbamate (2)** The solution of methyl (*S*)-2-((*tert*-butoxycarbonyl)amino)-3-((*S*)-2-oxopyrrolidin-3-yl)propanoate **1** (5.1 g, 17.8 mmol) in 7M NH_3 in methanol (50 mL) was stirred at room temperature for 48 h. TLC showed the reaction was completed. The reaction solution was concentrated in vacuum to afford compound **2** as light yellow oil (4.9 g, 100% yield). ^{1}H NMR (400 MHz, CD_3OD) δ 4.16-3.98 (m, 1H), 3.34-3.27 (m, 1H), 2.55 -2.42 (m, 1H), 2.40-2.28 (m, 1H), 2.09-1.96 (m, 1H), 1.93-1.81(m, 1H), 1.79-1.68 (m, 1H), 1.45 (s, 9H).

(*S*)-2-amino-3-((*S*)-2-oxopyrrolidin-3-yl)propanamide (3) To a solution of **2** (4.9 g, 18 mmol, 1 equiv) in CH_2Cl_2 (10 mL) was added TFA (10 mL) at 0 °C. The solution was stirred for 4 h at room temperature. Then the solution was concentrated. Ethyl ether was added and removed by means of dropper carefully so as not to disturb the residue. The residue was concentrated to give TFA salt of compound **3** as light yellow syrup (6.4 g, 100% yield). ^{1}H NMR (400 MHz, CD_3OD) δ 4.03 (dd, *J* = 8.9, 4.9 Hz, 1H), 3.39 (dd, *J* = 9.0, 4.6 Hz, 2H), 2.80-2.69 (m, 1H), 2.48-2.35 (m, 1H), 2.12-1.96 (m, 2H), 1.93-1.82 (m, 1H).

General procedure A for the synthesis of 5a-c

Boc-Tle-OH (1.05 equiv) and HCl salt of amino acid methyl ester (1.0 equiv) was dissolved in DMF and acetonitrile (v/v = 1/8). The resulting solution was cooled to 0 °C. Then HATU (1.05 equiv) and DIEA (4.0 equiv) was added. The resulting solution was stirred for 3.5 h at room temperature. A mixture of brine and water (1:1) was added, extracted with ethyl acetate (2×). The combined organic phase was washed with brine, dried over anhydrous Na_2SO_4, filtered, concentrated under vacuum. The crude product was purified by silica gel column chromatography to give **5a**-**c**.

5a, colorless oil, 97% yield.^{1}H NMR (400 MHz, $CDCl_3$) δ 7.32-7.27 (m, 2H), 7.26-7.20 (m, 1H), 7.12-7.06 (m, 2H), 6.08 (d, *J* = 7.3 Hz, 1H), 5.22 (d, *J* = 9.2 Hz, 1H), 4.91-4.80(m, 1H), 3.81 (d, *J* = 9.3 Hz, 1H), 3.72 (s, 3H), 3.19-3.03 (m, 2H), 1.45 (s, 9H), 0.95 (s, 9H).

5b, white solid, 86% yield. ^{1}H NMR (400 MHz, $CDCl_3$) δ 7.38-7.10 (m, 5H), 5.49 (dd, *J* = 9.9, 5.8 Hz, 1H), 5.08 (d, *J* = 9.9 Hz, 1H), 4.41 (d, *J* = 9.9 Hz, 1H), 3.72 (s, 1H), 3.40 (dd, *J* = 14.6, 5.8 Hz, 1H), 3.02 (s, 1H), 3.00- 2.72 (m, 1H), 1.41 (s, 9H), 0.96 (s, 9H).

5c, white solid, 92% yield. ^{1}H NMR (400 MHz, DMSO-d_6) δ 6.71 (d, *J* = 9.3 Hz, 1H), 4.21 (s, 1H), 4.06 (d, *J* = 9.4 Hz, 1H), 3.93 (d, *J* = 10.4 Hz, 1H), 3.85-3.76 (m, 1H), 3.65 (s, 3H), 1.56-1.49 (m, 1H), 1.44-1.39 (m, 1H), 1.36 (d, *J* = 6.0 Hz, 9H), 1.01 (s, 3H), 0.94 (s, 8H), 0.85 (s, 3H).

General procedure B for the synthesis of 6a-c

Compound **5a**-**c** (1.0 equiv) was dissolved in THF and H_2O (v/v = 3.5/1).

$LiOH.H_2O$ (2.0 equiv) was added at 0 → 5 °C. The reaction solution was stirred for 3 h at room temperature. TLC showed the reaction was completed. Water was added and cooled to 0 °C. The pH value of solution was adjusted to 3 with 1M HCl (aq). The resulting mixture was extracted with ethyl acetate. The organic layers were combined, washed with brine, dried and concentrated under vacuum to give compound **6a**-**c**. The product was used in the next step without further purification.

General procedure C for the synthesis of 7a-c

Compound **6a-c** (1.0 equiv) was dissolved in butan-2-one. The resulting solution was cooled to 0 °C. Followed by the addition of HOPO (0.25 equiv), compound **3** TFA salt (1.0 equiv). Then EDCI (1.1 equiv) and DIEA (3.5 equiv) was added. The reaction mixture was stirred at room temperature overnight. Ethyl acetate and a mixture of water and brine (1:1) was added. The organic layer was separated and washed with 0.2M HCl (1×), brine (2×), dried over anhydrous Na_2SO_4, filtered, concentrated under vacuum. The crude product was purified by silica gel column chromatography (PE→CH_2Cl_2: methanol = 15:1) to afford compound **7a-c**.

7a, off-white solid, 71% yield. ^{1}H NMR (400 MHz, DMSO-d_6) δ 8.15 (d, J = 8.5 Hz, 1H), 8.05 (d, J = 7.6 Hz, 1H), 7.57 (s, 1H), 7.30-7.11 (m, 5H), 6.99 (s, 1H), 6.91 (s, 1H), 6.40 (d, J = 9.7 Hz, 1H), 4.64-4.52 (m, 1H), 4.26-4.14 (m, 1H), 3.86 (d, J = 9.7 Hz, 1H), 3.18-2.94 (m, 3H), 2.88-2.75 (m, 1H), 2.27-2.17 (m, 1H), 2.15-2.04 (m, 1H), 2.00-1.90 (m, 1H), 1.66-1.55 (m, 1H), 1.54-1.43 (m, 1H), 1.37 (s, 9H), 0.79 (s, 9H).

7b, off-white solid, 84 % yield. ^{1}H NMR (400 MHz, DMSO-d_6) δ 8.23-7.92 (m, 1H), 7.76 -7.61 (m, 1H), 7.34-6.97 (m, 7H), 6.57-6.16 (m, 1H), 5.42-5.23(m, 1H), 4.36-4.08 (m, 2H), 3.26-3.00 (m, 4H), 2.99-2.82 (m, 2H), 2.21-2.05 (m, 2H), 1.98-1.90 (dm, 1H), 1.74 -1.59 (m, 1H), 1.54-1.46 (m, 1H), 1.40-1.29 (m, 9H), 0.95-0.48 (m, 9H).

7c, off-white solid, 89 % yield. ^{1}H NMR (400 MHz, DMSO-d_6) δ 8.24 (d, J = 8.7 Hz, 1H), 7.54 (s, 1H), 7.28 (s, 1H), 7.02 (s, 1H), 6.61 (d, J = 9.4 Hz, 1H), 4.31-4.22 (m, 2H), 4.13-3.97 (m, 1H), 3.90-3.78 (m, 2H), 3.12 (t, J = 9.0 Hz, 1H), 3.07-2.99 (m, 1H), 2.46-2.31(m, 1H), 2.17 -2.08 (m, 1H), 1.99-1.87 (m, 1H), 1.69-1.55 (m, 1H), 1.53-1.44 (m, 1H), 1.35 (s, 9H), 1.01 (s, 3H), 0.92 (s, 9H), 0.86 (s, 3H).

General procedure D for the synthesis of H109, H116, H119

To a solution of **7a-c** (1.0 equiv) in CH_2Cl_2 was added Burgess reagent (2.5 equiv). The resulting solution was stirred at room temperature for 2 h. Ethyl acetate and water was added. The organic layer was separated and washed with brine (3×), concentrated under vacuum. The crude product was purified by silica gel column chromatography. The product was further precipitated from ethyl ether. The solid was collected, dissolved in CH_3CN and H_2O, lyophilized to afford **H109**, **H116**, **H119**.

H109, white solid, 54 % yield. ^{1}H NMR (400 MHz, $CDCl_3$) δ 7.85-7.83 (m, 1H), 7.32-7.12 (m, 6H), 7.00-6.87 (m, 1H), 6.53 (s, 1H), 5.16 (d, *J* = 8.4 Hz, 1H), 4.89-4.70 (m, 2H), 3.81 (d, *J* = 8.3 Hz, 1H), 3.42-3.25 (m, 2H), 3.18-2.99 (m, 2H), 2.41-2.29 (m, 1H), 2.28-2.15 (m, 2H), 1.92-1.72 (m, 2H), 1.44 (s, 9H), 0.95 (s, 9H). ^{13}C NMR (101 MHz, DMSO-*d6*) δ 177.25, 170.90, 170.04, 155.04, 136.97, 128.95, 127.98, 126.29, 119.07, 77.95, 61.49, 53.81, 37.85, 37.10, 36.73, 34.03, 33.38, 30.86, 28.05, 26.77, 26.38, 21.96, 13.86. HRMS (ESI) calcd for $C_{27}H_{40}N_5O_5$[M + H]$^+$ 514.3029, found 514.3032. HPLC purity: 94.66%.

H116, white solid, 41% yield. ^{1}H NMR (400 MHz, $CDCl_3$) δ 8.18-8.00 (m, 1H), 6.47-6.16 (m, 1H), 5.32-5.07 (m, 1H), 5.02-4.90(m, 1H), 4.30 (s, 1H), 4.25-4.10(m, 1H), 4.04-3.84 (m, 2H), 3.45-3.28 (m, 2H), 2.58 (s, 2H), 2.48-2.24 (m, 2H), 2.02-1.75 (m, 2H), 1.60-1.46 (m, 3H), 1.43-1.23 (m, 9H), 1.06 (s, 3H), 1.02-0.89 (m, 10H). ^{13}C NMR (101 MHz, $CDCl_3$) δ 179.01, 171.66, 171.22, 155.91, 118.38, 79.72, 60.36, 58.68, 48.31, 40.46, 39.15, 37.63, 34.91, 34.05, 29.95, 28.30, 28.23, 27.84, 26.37, 26.22, 19.24, 12.56. HRMS (ESI) calcd for $C_{25}H_{39}N_5O_6$[M + H]$^+$ 504.3186, found 504.3184. HPLC purity: 97.76%.

H119, white solid, 45% yield. ^{1}H NMR (400 MHz, DMSO-*d6*) δ 9.00-8.37 (m, 1H), 7.80-7.64 (m, 1H), 7.37 -7.10 (m, 5H), 6.78-6.30 (m, 1H), 5.34-5.01 (m, 1H), 4.95-4.72 (m, 1H), 4.39-4.37 (m, 1H), 3.22-3.01 (m, 5H), 2.99-2.80 (m, 2H), 2.36-2.02 (m, 3H), 1.88 -1.62 (m, 2H), 1.45-1.23 (m, 9H), 0.95-0.61 (m, 9H).^{13}C NMR (101 MHz, DMSO-*d6*) δ 177.18, 171.70, 170.01, 155.25, 137.18, 128.49, 128.03, 126.18, 119.21, 77.94, 56.62, 55.93, 38.66, 36.83, 34.73, 33.79, 33.11, 32.22, 28.02, 27.83, 26.75, 26.09. MS (ESI) calcd for $C_{28}H_{42}N_5O_5$ [M + H]$^+$ 528.3186, found 528.3195. HPLC purity: 99.36%.

Synthesis of 9a

1-(*tert*-butyl) 2-methyl (2*S*,4*R*)-4-hydroxypyrrolidine-1,2-dicarboxylate **8a** (3.1 g, 12.6 mmol, 1.0 equiv) was dissolved in DMF (35 mL) and cooled to 0°C. Followed by the addition of NaH (1.0 g, 25.3 mmol, 2.0 equiv). The resulting solution was stirred at 0°C for 20 min. Then CH_3I (4.3 g, 30 mmol, 2.4 equiv) was added. The resulting reaction solution was stirred at 0°C for 1h. Then the solution was cooled to 0°C, quenched with NH_4Cl solution, extracted with ethyl acetate. The organic layer was separated and washed with brine (3×), concentrated under vacuum. The crude product was purified by silica gel column chromatography eluted with 40% EtOAc/petroleum ether → 45% EtOAc/ petroleum ether to afford compound **9a** as light yellow oil (2.9 g, 90% yield). ^{1}H NMR (400 MHz, $CDCl_3$) δ 4.45-4.26 (m, 1H), 4.02-3.90 (m, 1H), 3.80-3.71 (m, 3H), 3.68-3.44 (m, 2H), 3.30 (d, *J* = 18.7 Hz, 3H), 2.40-2.18 (m, 1H), 2.16-1.92 (m, 1H), 1.52-1.34 (m, 9H).

Synthesis of 9b

1-(*tert*-butyl) 2-methyl (2*S*,4*R*)-4-hydroxypyrrolidine-1,2-dicarboxylate **8a** (3.0 g, 12.2 mmol, 1.0 equiv) was dissolved in DMF (40 mL) and cooled to 0°C. Followed by the addition of NaH (1.0 g, 24.4 mmol, 2.0 equiv). The resulting solution was stirred at 0°C for 20 min. Then benzyl bromide (4.1 g, 24.4 mmol, 2.2 equiv) was added. The resulting reaction solution was stirred at room temperature for 5h. Then the solution was cooled to 0°C, quenched with NH_4Cl solution, extracted with ethyl acetate. The organic layer was separated and washed with brine (3×), concentrated under vacuum. The crude product was purified by silica gel column chromatography eluted with 35% EtOAc/petroleum ether to afford compound **9b** as colorless oil (3.6 g, 89 % yield). ^{1}H NMR (400 MHz, DMSO-d_6) δ 7.39-7.23 (m, 5H), 4.55-4.47 (m, 1H), 4.46-4.40 (m, 1H), 4.36-4.10 (m, 1H), 3.70-3.56 (m, 1H), 3.55-3.40 (m, 1H), 2.44-2.30 (m, 1H), 2.20-2.01 (m, 1H), 1.44-1.28(, 9H).

Synthesis of (2*S*,4*R*)-1-*tert*-butyl 2-methyl 4-(difluoromethoxy)-pyrrolidine-1,2-dicarboxylate (9c)

To a solution of 1-(*tert*-butyl) 2-methyl (2*S*,4*R*)-4-hydroxypyrrolidine-1,2-dicarboxylate **8a** (4.0 g, 16 mmol, 1 equiv) in acetonitrile (40 mL) was added CuI (621

mg, 32.6 mmol, 0.2 equiv). 2-fluorosulfonyl-2,2-difluoroacetic acid (3.5 g, 19.6 mmol, 1.2 equiv) in 30 mL of acetonitrile was added dropwise over a period of 20 min. The reaction solution was stirred at 50 °C for 1 h. Then acetonitrile was evaporated, saturated NH_4Cl solution was added, the resulting mixture was extracted with ethyl acetate. The combined organic phase was washed with brine, dried over anhydrous Na_2SO_4, filtered, concentrated under vacuum. The crude product was purified by silica gel column chromatography (40% EtOAc/petroleum ether → 45% EtOAc/ petroleum ether) to afford compound **9c** as light yellow oil (3.47 g, 72% yield). ^{1}H NMR (400 MHz, DMSO-*d*6) δ 6.75 (t, J = 75.4 Hz, 1H), 4.85-4.76 (m, 1H), 4.28-4.17 (m, 1H), 3.71-3.64 (m, 3H), 3.60-3.44 (m, 2H), 2.45-2.31 (m, 1H), 2.21-2.08 (m, 1H), 1.40 (s, 3H), 1.34 (s, 6H). ^{19}F NMR (376 MHz, DMSO-*d*6) δ -81.32 to -81.08 (m, 2F).

Synthesis of 9d

To round bottom flask was added 1-(*tert*-butyl) 2-methyl (2*S*,4*R*)-4-hydroxypyrrolidine-1,2-dicarboxylate **8a** (8 g, 32.4 mmol, 1 equiv), AgOTf (25 g, 97.2 mmol, 3.0 equiv), Selectfluor (17.2 g, 21.6 mmol, 1.5 equiv), KF (7.5 g, 129.6 mmol, 4.0 equiv) successively in a nitrogen-filled glovebox. Then ethyl acetate (130 mL), 2-fluoropyridine (9.4 g, 97.2 mmol, 3.0 equiv) and CF_3TMS (13.8 g, 97.2 mmol, 3.0 equiv) were added. The reaction mixture was stirred at room temperature for 12 h in a nitrogen-filled glovebox. The reaction mixture was filtered (eluted with ethyl acetate). The filtrate was washed with 0.1N HCl, brine, dried and concentrated under vacuum. The crude product was purified by silica gel column chromatography eluted with 40% EtOAc/petroleum ether ether to afford compound **9d** as colorless oil (2.7g, 27% yield). ^{1}H NMR (400 MHz, DMSO-*d*6) δ 5.14- 5.02 (m, 1H), 4.35-4.23 (m, 1H), 3.76-3.53 (m, 5H), 2.34-2.20 (m, 1H), 1.45-1.29 (m, 9H). ^{19}F NMR (376 MHz, DMSO-*d*6) δ -57.09 (s, 3F).

Synthesis of 9e

The solution of 1-(*tert*-butyl) 2-methyl (2*S*,4*S*)-4-hydroxypyrrolidine-1,2-dicarboxylate **8b** (3.5 g, 14.3 mmol, 1 equiv) and PhOH(1.8g, 18.6 mmol, 1.3 equiv) was cooled to cooled to 0°C. Then triphenylphosphine (4.9 g, 18.6 mmol, 1.3 equiv) and azo DEAD (3.8 g, 18.6 mmol, 1.3 equiv) was added successively under N_2

atmosphere overnight. The reaction solution was concentrated. The crude product was purified by silica gel column chromatography eluted with 25% EtOAc/petroleum ether ether to afford compound **9e** as white solid (2.9 g, 63% yield). ^{1}H NMR (400 MHz, DMSO-*d6*) δ 7.33-7.26 (m, 2H), 7.04-6.92 (m, 3H), 5.07-4.99 (m, 1H), 4.36-4.24 (m, 1H), 3.72-3.62 (m, 3H), 3.61-3.52 (m, 2H), 2.47-2.39 (m, 1H), 2.27-2.15 (m, 1H), 1.44 (d, *J* = 10.9 Hz, 9H).

Synthesis of 9f

2,6-lutidine (1.3 g, 12 mmol, 0.5 equiv) and bis(trifluoromethane)sulfonimide (169 mg, 0.6 mmol, 0.025 equiv) was added to a round bottom flask in a nitrogen-filled glovebox to prepared catalyst. The resulting mixture was added to the1-(*tert*-butyl) 2-methyl (2*S*,4*R*)-4-hydroxypyrrolidine-1,2-dicarboxylate **8** (6.7 g, 24 mmol, 1.0 equiv) in fluorobenzene (40 mL). Followed by the addition of tert-butyl-2,2,2-trichloroacetimidate (TBTA) (26.22 g, 1.03 mL, 120 mmol, 5.0 equiv) dropwise over the course of 20 min. The reaction mixture was stirred at room temperature overnight. Then EtOAc and NH_4Cl was added. the organic layer was separated and washed with 0.1N HCl, brine, dried and concentrated under vacuum. The crude product was purified by eluted with 40% EtOAc/petroleum ether ether to afford compound **9f** (7.5 g, 93% yield).

Synthesis of 10a-e

To a solution of compound **9a-e** in CH_2Cl_2 (5 mL) was added TFA (5 mL) at 0 °C. The solution was stirred for 3 h at room temperature. Then the solution was concentrated to give TFA salt of compound **9a-e**. The product was used directly for the next step without further purification.

Synthesis of 10f

Compound **9f** (7.5 g) is dissolved in anhydrous methanol (90 mL) in round bottom flask. The atmosphere of flask was degassed and purged with nitrogen. 10% Pd/C (1.3 g, 15 wt%) was added. The flask is then degassed and purged with hydrogen. The mixture is filtered through celite, and the filtrate was concentrated to give **10f** as light syrup (4.3 g, 95% yield).

Synthesis of 11a-f

Compound **11a-f** was synthesized following general procedure A described above using Boc-Tle-OH **4** reacted with **10a-f**. The crude product was purified by silica gel column chromatography.

11a, colorless oil, 64% yield. ^{1}H NMR (400 MHz, DMSO-*d6*) δ 6.67 (d, *J* = 9.3 Hz, 1H), 4.26 (t, *J* = 8.6 Hz, 1H), 4.22-4.15 (m, 1H), 4.06-3.93 (m, 1H), 3.66-3.58 (m, 2H), 3.23 (s, 1H), 2.36-2.25 (m, 1H), 1.96-1.85 (m, 1H), 1.37 (s, 9H), 1.10-0.79 (m, 9H).

11b, white solid, 44% yield. ^{1}H NMR (400 MHz, DMSO-*d6*) δ 7.49-7.15 (m, 5H), 6.66 (d, *J* = 9.2 Hz, 1H), 4.56-4.42 (m, 2H), 4.30-4.18 (m, 3H), 4.11 (d, *J* = 11.2 Hz, 1H), 3.66-3.57 (m, 2H), 2.41-2.25 (m, 1H), 2.10-1.90 (m, 1H), 1.37 (s, 9H), 0.91 (s, 9H).

11c, white solid, 70% yield. ^{1}H NMR (400 MHz, DMSO-*d6*) δ 7.02-6.52 (m, 2H), 4.94-4.78 (m, 1H), 4.35 (t, *J* = 8.4 Hz, 1H), 4.20-4.11 (m, 1H), 4.06 -3.93 (m, 1H), 3.86-3.77 (d, *J* = 7.9 Hz, 1H), 3.62 (d, *J* = 2.7 Hz, 3H), 2.44-2.33 (m, 1H), 2.19-2.07 (m, 1H), 1.37 (s, 9H), 0.94 (s, 9H). ^{19}F NMR (376 MHz, DMSO-*d6*) δ -81.96 to -80.88 (m, 2F).

11d, white solid, 63% yield. ^{1}H NMR (400 MHz, $CDCl_3$) δ 5.28-5.15 (m, 1H), 4.96-4.85 (m, 1H), 4.75-4.61 (m, 1H), 4.30-4.09 (m, 2H), 3.96-3.87 (m, 1H), 3.81-3.73 (m, 3H), 2.62-2.48 (m, 1H), 2.31-2.12 (m, 1H), 1.43 (s, 9H), 1.05-0.95 (m, 9H). ^{19}F NMR (376 MHz, DMSO-*d6*) δ -58.90 to -59.05 (m, 3F).

11e, white solid, 66% yield. ^{1}H NMR (400 MHz, $CDCl_3$) δ 7.35-7.25 (m, 2H), 7.01 (t, *J* = 7.4 Hz, 1H), 6.90-6.80 (m, 2H), 5.31-5.23 (m, 1H), 5.04-4.92 (m, 1H), 4.70 (t, *J* = 8.4 Hz, 1H), 4.29 (d, *J* = 9.8 Hz, 1H), 4.21-4.12 (m, 1H), 3.96-3.87 (m, 1H), 3.78-3.73 (m, 1H), 2.75-2.55 (m, 1H), 2.27-2.15 (m, 1H), 1.50-1.34 (m, 9H), 1.12 -1.02 (m, 9H).

11f, light yellow oil, 65% yield. ^{1}H NMR (400 MHz, DMSO-*d6*) δ 6.67 (d, *J* = 9.1 Hz, 1H), 4.40-4.27 (m, 2H), 4.16 (d, *J* = 9.4 Hz, 1H), 3.75-3.56 (m, 4H), 2.11-1.87 (m, 2H), 1.37 (d, *J* = 7.3 Hz, 9H), 1.13 (d, *J* = 8.7 Hz, 9H), 0.93 (s, 9H).

Synthesis of 12a-f

Compound **12a-f** was synthesized following general procedure B described above

using **11a-f** as start material. The crude product was used directly for the next step.

Synthesis of 13a-f

Compound **13a-f** was synthesized following general procedure C described above using **12a-f** reacted with TFA salt of compound **3**. The crude product was purified by silica gel column chromatography.

13a, white solid, 44 % yield.

13b, white solid, 72 % yield.^{1}H NMR (400 MHz, DMSO-*d6*) δ 8.21 (d, *J* = 8.9 Hz, 1H), 7.52 (s, 1H), 7.38-7.20 (m, 5H), 7.01 (s, 1H), 6.67 (d, *J* = 9.1 Hz, 1H), 4.54-4.34 (m, 3H), 4.31-4.06 (m, 4H), 3.65-3.56 (m, 1H), 3.17 -2.97 (m, 2H), 2.30-2.09 (m, 2H), 1.95 -1.83 (m, 1H), 1.65-1.41 (m, 2H), 1.34 (s, 8H), 0.93 (s, 9H).

13c, white solid, 62 % yield. ^{1}H NMR (400 MHz, DMSO-*d6*) δ 8.30 (d, *J* = 8.9 Hz, 1H), 7.54 (s, 1H), 7.29 (s, 1H), 7.02 (s, 1H), 6.98-6.55 (m, 2H), 4.96-4.74 (m, 1H), 4.51-4.38 (m, 1H), 4.33-4.22 (m, 1H), 4.15-4.05 (m, 1H), 3.99-3.87 (m, 1H), 3.86 -3.73 (m, 1H), 3.30 (s, 1H), 3.19-2.97 (m, 2H), 2.35-2.23 (m, 1H), 2.22- 2.11 (m, 1H), 2.09 -1.90 (m, 2H), 1.70-1.56 (m, 1H), 1.54-1.43 (m, 1H), 1.37 (s, 9H), 0.92 (s, 9H).

13d, white solid, 62 % yield. ^{1}H NMR (400 MHz, DMSO-*d6*) δ 8.32 (d, *J* = 8.9 Hz, 1H), 7.54 (s, 1H), 7.31 (s, 1H), 7.03 (s, 1H), 6.78 (d, *J* = 8.9 Hz, 1H), 5.22-5.12 (m, 1H), 4.55-4.39(m, 1H), 4.34-4.22 (m, 1H), 4.18-4.02 (m, 2H), 3.88-3.78 (m, 1H), 3.17-2.96 (m, 2H), 2.45-2.35 (m, 1H), 2.23-2.05 (m, 2H), 2.01-1.89 (m, 1H), 1.70-1.56 (m, 1H), 1.54-1.44 (m, 1H), 1.36 (s, 7H), 0.93 (s, 9H). ^{19}F NMR (376 MHz, DMSO-*d6*) δ -56.94 (s).

13e, off white solid, 73 % yield. ^{1}H NMR (400 MHz, DMSO-*d6*) δ 8.27 (d, *J* = 8.8 Hz, 1H), 7.54 (s, 1H), 7.30 (t, *J* = 7.9 Hz, 3H), 7.06-6.90 (m, 4H), 6.65 (d, *J* = 8.8 Hz, 1H), 5.15-5.04 (m, 1H), 4.52-4.40 (m, 1H), 4.33-4.24 (m, 1H), 4.18-4.08 (m, 1H), 4.04-3.95 (m, 1H), 3.94-3.82 (m, 1H), 3.19-2.99 (m, 2H), 2.43-2.32 (m, 1H), 2.24- 2.14 (m, 1H), 2.12-2.02 (m, 1H), 1.98-1.91 (m, 1H), 1.70-1.42 (m, 2H), 1.40-1.22 (m, 9H), 0.94 (s, 9H).

13f, off white solid, 49 % yield. ^{1}H NMR (400 MHz, DMSO-*d6*) δ 8.19 (d, *J* = 8.8 Hz, 1H), 7.55 (s, 1H), 7.21 (s, 1H), 7.02 (s, 1H), 6.67 (d, *J* = 9.6 Hz, 1H), 4.43-4.31 (m, 2H), 4.27-4.19 (m, 1H), 4.13 (d, *J* = 9.3 Hz, 1H), 3.74-3.68 (m, 1H), 3.63-3.56 (m, 1H),

3.21-2.98 (m, 2H), 2.21-2.08 (m, 1H), 2.03-1.87 (m, 3H), 1.67-1.55 (m, 1H), 1.54-1.45 (m, 1H), 1.36 (s, 9H), 1.15 (s, 9H), 0.90 (s, 9H).

General procedure E for the synthesis of 14

To a solution of **13c** (2.38 g, 4.35 mmol, 1 equiv) in CH_2Cl_2 (8 mL) was added TFA (8 mL) at 0 °C. The solution was stirred for 4 h at room temperature. Then the solution was concentrated. Ethyl ether was added and removed by means of dropper carefully so as not to disturb the precipitate. The resulting residue was concentrated to give TFA salt of compound **14** as light yellow solid (3.5 g, 100% yield). ^{1}H NMR (400 MHz, DMSO-*d6*) δ 8.42 (d, *J* = 9.1 Hz, 1H), 8.11-7.99 (m, 3H), 7.55 (s, 1H), 7.40 (s, 1H), 7.04 (s, 1H), 6.81 (t, *J* = 75.5 Hz, 1H), 4.93-4.88 (m, 1H), 4.62-4.53 (m, 1H), 4.40-4.29 (m, 1H), 4.08-3.96 (m, 2H), 3.74-3.67 (m, 2H), 3.20-2.99 (m, 3H), 2.49-2.41 (m, 1H), 2.39-2.31(m, 1H), 2.24-2.13 (m, 1H), 2.07-1.91 (m, 2H), 1.76-1.61 (m, 1H), 1.53-1.42 (m, 1H), 1.02 (s, 9H).

Synthesis of 15a-b

Compound **15a-b** was synthesized following general procedure C described above using TFA salt of **14** reacted with 4,4-difluorocyclohexanecarboxylic acid or 1-fluoro-cyclopropanecarboxylic acid. The crude product was purified by silica gel column chromatography.

15a, off white solid, 80 % yield. ^{1}H NMR (400 MHz, DMSO-*d6*) δ 8.29 (d, *J* = 9.0 Hz, 1H), 7.98 (d, *J* = 9.2 Hz, 1H), 7.54 (s, 1H), 7.32 (s, 1H), 7.04 (s, 1H), 6.76 (t, *J* = 75.5 Hz, 1H), 4.94-4.76 (m, 1H), 4.54 - 4.37 (m, 2H), 4.36-4.25 (m, 1H), 3.93-3.77 (m, 2H), 3.21- 2.96 (m, 2H), 2.32- 2.13 (m, 2H), 2.10-1.89 (m, 4H), 1.85-1.40 (m, 8H), 0.93 (s, 9H). ^{19}F NMR (376 MHz, DMSO-*d6*) δ -82.02 to -80.41 (m), -90.00 (d, *J* = 232.2 Hz), -99.05 (d, *J* = 234.4 Hz).

15b, off white solid, 61 % yield. ^{1}H NMR (400 MHz, DMSO-*d6*) δ 8.32 (d, *J* = 8.9 Hz, 1H), 7.55 (s, 1H), 7.43-7.28 (m, 2H), 7.03 (s, 1H), 6.76 (t, *J* = 75.3 Hz, 1H), 4.94-4.86 (m, 1H), 4.57- 4.44 (m, 2H), 4.37-4.23 (m, 1H), 3.91-3.78 (m, 2H), 3.20 - 2.97 (m, 2H), 2.34-2.26 (m, 1H), 2.24-2.13 (m, 1H), 2.10-1.85 (m, 2H), 1.73-1.59 (m, 1H), 1.56-1.43 (m, 1H), 1.41-1.12 (m, 4H), 0.95(s, 9H). ^{19}F NMR (376 MHz, DMSO-*d6*) δ -84.70 to -77.87 (m), -196.04 (s).

Synthesis of H117-H118, H121, H123-124, H138-H140

Target nitrile compound **H117**, **H118**, **H121**, **H123**, **H124**, **H138**, **H139** and **H140** were synthesized following general procedure D using corresponding amide **13a**-**f** or **15a**-**b** as start material.

H138, white solid, 55 % yield. ^{1}H NMR (400 MHz, DMSO-*d6*) δ 8.94 (d, *J* = 8.6 Hz, 1H), 7.65 (s, 1H), 6.70 (d, *J* = 9.2 Hz, 1H), 5.00-4.90 (m, 1H), 4.21 (t, *J* = 8.4 Hz, 1H), 4.14 (d, *J* = 9.2 Hz, 1H), 4.07-3.92 (m, 2H), 3.63-3.51 (m, 1H), 3.23 (s, 3H), 3.16-2.99 (m, 2H), 2.58-2.52 (m, 1H), 2.26-2.04 (m, 3H), 1.92-1.81 (m, 1H), 1.75-1.63 (m, 2H), 1.37(s, 9H), 0.92 (s, 9H). ^{13}C NMR (101 MHz, DMSO-*d6*) δ 178.1, 171.9, 170.5, 156.1, 120.2, 79.1, 78.6, 58.8, 58.7, 55.9, 52.7, 38.0, 37.1, 35.2, 35.1, 34.7, 31.4, 28.6, 27.4, 26.7, 22.5, 14.4. HRMS (ESI) calcd for $C_{36}H_{48}N_5O_8$ [M + H]$^+$ 494.2979, found 494.2985. HPLC purity: 98.51%.

H139, white solid, 49 % yield. H NMR (400 MHz, DMSO-*d6*) δ 8.96 (d, *J* = 8.6 Hz, 1H), 7.67 (s, 1H), 7.39 -7.24 (m, 5H), 6.76 (d, *J* = 8.9 Hz, 1H), 5.02-4.91(m, 1H), 4.59-4.40 (m, 2H), 4.35-4.22 (m, 2H), 4.16 (d, *J* = 9.5 Hz, 2H), 3.62 (d, *J* = 8.6 Hz, 1H), 3.17-2.98 (m, 2H), 2.61-2.52 (m, 1H), 2.30-2.05 (m, 3H), 1.97-1.85 (m, 1H), 1.75-1.60 (m, 2H), 1.35 (s, 9H), 0.94 (s, 9H). ^{13}C NMR (101 MHz, DMSO-*d6*) δ 178.10, 171.97, 170.71, 156.24, 138.58, 128.61, 128.22, 127.93, 120.22, 78.55, 77.38, 69.98, 59.10, 58.87, 53.02, 49.20, 37.99, 37.03, 35.56, 35.05, 34.77, 28.58, 27.39, 27.30, 26.72. MS (ESI) calcd for $C_{30}H_{44}N_5O_6$ [M + H]$^+$ 570.3292, found 570.3291. HPLC purity: 99.02%.

H117, white solid, 70% yield. ^{1}H NMR (400 MHz, DMSO-*d6*) δ 9.03 (d, *J* = 8.4 Hz, 1H), 7.66 (s, 1H), 7.04-6.52 (m, 2H), 5.09-4.81 (m, 2H), 4.31 (t, *J* = 8.1 Hz, 1H), 4.15-4.06 (m, 1H), 4.03-3.90 (m, 1H), 3.89-3.70 (m, 1H), 3.21-2.99 (m, 2H), 2.36-1.96 (m, 4H), 1.76-1.61 (m, 2H), 1.41-1.23 (m, 9H), 0.98-0.81 (m, 9H). ^{13}C NMR (101 MHz, DMSO-*d6*) δ 177.57, 170.95, 170.06, 155.67, 119.64, 116.93 (t, *J* = 255.6 Hz), 78.15, 74.68, 58.48, 57.95, 53.60, 37.66, 36.61, 35.25, 34.80, 34.25, 30.96, 28.11, 26.92, 26.17, 22.07, 13.96.^{19}F NMR (376 MHz, DMSO-*d6*) δ -82.34 to -80.16 (m). HRMS (ESI) calcd for $C_{24}H_{38}F_2N_4O_6$[M + H]$^+$ 530.2790, found 530.2786. HPLC purity: 98.03%.

H121, white solid, 50% yield.^{1}H NMR (400 MHz, DMSO-*d6*) δ 9.06 (d, *J* = 8.5 Hz, 1H), 7.68 (s, 1H), 6.86 (d, *J* = 9.0 Hz, 1H), 5.19 (s, 1H), 5.02-4.93 (m, 1H), 4.38-

4.28 (m, 1H), 4.22-4.02 (m, 2H), 3.90-3.80 (m, 1H), 3.17-2.99 (m, 2H), 2.57-2.52 (m, 1H), 2.44-2.34 (m, 1H), 2.25-2.05 (m, 3H), 1.78-1.61 (m, 2H), 1.36 (s, 9H), 0.92 (s, 9H). ^{13}C NMR (101 MHz, DMSO-*d6*) δ 177.45, 170.59, 170.14, 155.68, 121.62 (q, *J* = 254.7 Hz). 119.79, 78.32, 78.07, 58.47, 57.60, 53.47, 48.63, 37.58, 36.49, 34.93, 34.44, 34.13, 27.94, 26.79, 26.72, 26.05. MS (ESI) calcd for $C_{24}H_{37}F_3N_5O_6$ $[M + H]^+$ 548.2696, found 548.2704. HPLC purity: 95.89%.

H140, white solid, 39% yield. ^{1}H NMR (400 MHz, DMSO-*d6*) δ 8.97 (d, *J* = 8.6 Hz, 1H), 7.64 (s, 1H), 7.30 (t, *J* = 7.9 Hz, 2H), 7.00-6.86 (m, 3H), 6.73-6.61 (m, 1H), 5.19-5.04 (m, 1H), 5.03-4.91 (m, 1H), 4.43-4.29 (m, 1H), 4.13-3.98 (m, 2H), 3.94-3.82 (m, 1H), 3.15-2.94 (m, 2H), 2.41-2.32 (m, 1H), 2.24-2.01 (m, 3H), 1.79 -1.58 (m, 2H), 1.50-1.14 (m, 9H), 0.93 (s, 9H). MS (ESI) calcd for $C_{29}H_{42}N_5O_2$ $[M + H]^+$ 556.3135, found 556.3137. HPLC purity: 99.67%.

H118, white solid, 45% yield. ^{1}H NMR (400 MHz, DMSO-*d6*) δ 8.90 (d, *J* = 8.5 Hz, 1H), 7.65 (s, 1H), 6.70 (d, *J* = 9.3 Hz, 1H), 5.01-4.87 (m, 1H), 4.37 (s, 1H), 4.27 (t, *J* = 7.9 Hz, 1H), 4.12 (d, *J* = 9.3 Hz, 1H), 3.80-3.45 (m, 2H), 3.18-2.97 (m, 2H), 2.23-2.03 (m, 2H), 2.02-1.82 (m, 2H), 1.74-1.58 (m, 2H), 1.36 (s, 9H), 1.15 (s, 9H), 0.91 (s, 9H). ^{13}C NMR (101 MHz, DMSO-*d6*) δ 177.60, 171.56, 169.96, 155.62, 119.71, 78.04, 73.57, 69.58, 58.35, 55.48, 37.59, 37.34, 36.62, 34.68, 34.23, 28.14, 27.98, 26.93, 26.18. MS (ESI) calcd for $C_{27}H_{46}N_5O_6$ $[M + H]^+$ 536.3448, found 536.3447. HPLC purity: 95.94 %.

H123, white solid, 59% yield. ^{1}H NMR (400 MHz, DMSO-*d6*) δ 9.04 (d, *J* = 8.8 Hz, 1H), 8.00 (d, *J* = 9.1 Hz, 1H), 7.68 (s, 1H), 6.78 (t, *J* = 75.4 Hz, 1H), 5.02-4.86 (m, 2H), 4.43 (d, *J* = 9.1 Hz, 1H), 4.31-4.23 (m, 1H), 3.99-3.76 (m, 2H), 3.18-3.10 (m, 1H), 3.06 -3.00 (m, 1H), 2.35-2.24 (m, 1H), 2.22-1.98 (m, 5H), 1.91-1.62 (m, 6H), 1.61-1.49 (m, 2H), 0.93 (s, 9H). ^{13}C NMR (101 MHz, DMSO-*d6*) δ 178.10, 174.49, 171.35, 170.11, 124.40 (t, *J* = 240.4 Hz), 120.13, 117.40 (t, *J* = 255.7 Hz), 75.10, 58.44, 56.79, 54.24, 38.05, 37.04, 35.76, 35.43, 34.87, 32.76 (dd, *J* = 47.1, 22.5 Hz), 31.43, 27.33, 25.65. MS (ESI) calcd for $C_{26}H_{38}F_4N_5O_5$ $[M + H]^+$ 576.2809, found 576.2802. HPLC purity: 98.34%.

H124, white solid, 65% yield. ^{1}H NMR (400 MHz, DMSO-*d6*) δ 9.08 (d, *J* = 8.5

Hz, 1H), 7.70 (s, 1H), 7.49-7.40 (m, 1H), 6.78 (t, *J* = 75.3 Hz, 1H), 5.11-4.84 (m, 2H), 4.54 (d, *J* = 9.0 Hz, 1H), 4.42- 4.23 (m, 1H), 3.96-3.74 (m, 2H), 3.19-3.10 (m, 1H), 3.10-3.00 (m, 1H), 2.47-2.41 (m, 1H), 2.37-2.26 (m, 1H), 2.21-1.94 (m, 3H), 1.78-1.62 (m, 2H), 1.44 -1.15 (m, 4H), 0.97 (s, 9H). ^{13}C NMR (101 MHz, DMSO-*d6*) δ 177.42, 170.65, 168.84, 168.39(d, *J* = 20.8Hz), 119.48, 116.81(t, *J* = 256.0 Hz), 77.71 (d, *J* = 232.5 Hz), 74.39, 57.92, 56.59, 53.83, 48.62, 37.71, 36.61, 35.34, 35.11, 34.05, 26.81, 26.72, 25.95, 12.82(t, *J* = 10.0 Hz). MS (ESI) calcd for $C_{23}H_{33}F_3N_5O_5$ [M + H]$^+$ 516.2434, found 516.2437. HPLC purity: 97.65%.

(2*S*,4*R*)-1-((*S*)-2-amino-3,3-dimethylbutanoyl)-4-(difluoromethoxy)pyrrolidine-2-carboxylic acid (16)

To a solution of **11b** (1.67 g, 4.2 mmol, 1 equiv) in CH_2Cl_2 (4 mL) was added TFA (4 mL) at 0 °C. The solution was stirred for 4 h at room temperature. Then the solution was concentrated to give TFA salt of compound **16** (2.78g, 100% yield) as light yellow oil.

(2*S*,4*R*)-4-(difluoromethoxy)-1-((*S*)-3,3-dimethyl-2-(2,2,2-trifluoroacetamido) butanoyl)pyrrolidine-2-carboxylic acid (17)

TFA salt of compound **16** (2.78 g, 4.2 mmol, 1 equiv) was dissolved in methanol (20 mL) and cooled to 0 °C. Triethylamine (1.7 g, 16.8 mol, 4 equiv) and trifluoroacetate (958 mg, 6.7 mmol, 1.6 equiv) was added. The resulting reaction solution was stirred at room temperature overnight. Then concentrated under vacuum and water was added. The pH value of solution was adjusted to 2-3 with 1M HCl (aq) and extracted with EtOAc. The organic layer was separated and washed with brine (3×), dried and concentrated under vacuum to give **17** as light yellow solid (1.5 g, 92% yield). ^{1}H NMR (400 MHz, DMSO-*d6*) δ 9.39 (d, *J* = 8.6 Hz, 1H), 6.76 (t, *J* = 75.2 Hz, 1H), 5.01-4.73 (m, 1H), 4.60-4.45 (m, 1H), 4.32 (t, *J* = 8.5 Hz, 1H), 4.01-3.67 (m, 2H), 2.44-2.32 (m, 1H), 2.19-2.07 (m, 1H), 0.97 (s, 9H). ^{19}F NMR (376 MHz, DMSO-*d6*) δ -72.99 (s), -81.45 to -80.95 (m).

(2*S*,4*R*)-*N*-((*S*)-1-amino-1-oxo-3-((*S*)-2-oxopyrrolidin-3-yl)propan-2-yl)-4-(difluoromethoxy)-1-((*S*)-3,3-dimethyl-2-(2,2,2-trifluoroacetamido)butanoyl) pyrrolidine-2-carboxamide (18)

Compound **18** was synthesized by following the general procedure C as described above using compound **17** reacting with TFA salt of compound **3**. The crude product

was purified by silica gel column chromatography (PE→CH_2Cl_2: methanol = 15:1) to afford compound **18** as off white solid (670 mg, 62 % yield). ^{1}H NMR (400 MHz, DMSO-*d6*) δ 9.49 (d, *J* = 8.8 Hz, 1H), 8.33 (d, *J* = 9.0 Hz, 1H), 7.54 (s, 1H), 7.34 (s, 1H), 7.03 (s, 1H), 6.77 (t, *J* = 75.3 Hz, 1H), 4.97-4.71 (m, 1H), 4.55 (d, *J* = 8.9 Hz, 1H), 4.47 (t, *J* = 8.2 Hz, 1H), 4.39 -4.27 (m, 1H), 3.93-3.79 (m, 2H), 3.21-2.93 (m, 2H), 2.36-2.26 (m, 1H), 2.24-2.13 (m, 1H), 2.10 -1.88 (m, 3H), 1.74-1.57 (m, 1H), 1.54-1.40 (m, 1H), 0.99 (s, 9H). ^{19}F NMR (376 MHz, DMSO-*d6*) δ -72.99 (s), -81.18 to -80.78 (m).

(2*S*,4*R*)-*N*-((*S*)-1-cyano-2-((*S*)-2-oxopyrrolidin-3-yl)ethyl)-4-(difluoromethoxy)-1-((*S*)-3,3-dimethyl-2-(2,2,2-trifluoroacetamido)butanoyl)pyrrolidine-2-carbox-amide (H122)

H122 was synthesized following the general procedure D described above using **18** as start material. The crude product was purified by silica gel column chromatography (PE→CH_2Cl_2: methanol = 35:1) to give 340 mg product. The product was precipitated from EtOAc:Et_2O = 2:1, dried at 50 °C under vacuum to afford **H122** as off white solid (325 mg, 54 % yield).^{1}H NMR (400 MHz, DMSO-*d6*) δ 9.52 (d, *J* = 8.2 Hz, 1H), 9.09 (d, *J* = 8.7 Hz, 1H), 7.69 (s, 1H), 6.79 (t, *J* = 75.2 Hz, 1H), 5.04-4.88 (m, 2H), 4.54 (d, *J* = 8.3 Hz, 1H), 4.37-4.26 (m, 1H), 3.96-3.80 (m, 2H), 3.19-2.98 (m, 2H), 2.38-2.27(m, 1H), 2.23-1.98 (m, 3H), 1.78-1.63 (m, 2H), 0.98 (s, 9H). ^{13}C NMR (101 MHz, DMSO-*d6*) δ 177.38, 170.52, 167.71, 156.56 (q, *J* = 37.0 Hz), 119.39, 117.05 (q, *J* = 288.9 Hz), 116.70 (t, *J* = 255.0 Hz), 74.30, 57.92, 57.71, 53.88, 37.46, 36.43, 35.04, 35.00, 35.14, 34.20, 26.66, 25.85. ^{19}F NMR (376 MHz, DMSO-*d6*) δ -72.91 (s), -81.35 to -80.65 (dd, *J* = 75.2, 3.0 Hz). MS (ESI) calcd for $C_{21}H_{29}F_5N_5O_5$ [M + H]$^+$ 526.2089, found 526.2090. HPLC purity: 99.13%.

Synthesis of 19c-d

Compound **11c-d** (1.0 equiv) was dissolved in THF and H_2O (v/v = 3.5/1). LiOH.H_2O (2.0 equiv) was added at 0 → 5 °C. The reaction solution was stirred for 3.5 h at room temperature. The residue was diluted with water and cooled to 0 °C. The pH value of solution was adjusted to 3 with 1M HCl (aq). The resulting mixture was extracted with ethyl acetate. The organic layers were combined, washed with brine,

dried and concentrated under vacuum to give compound **19c-d**. The product was used in the next step without further purification.

19c, light yellow oil. ^{1}H NMR (400 MHz, DMSO-*d6*) δ 12.60 (s, 1H), 6.75 (t, *J* = 75.4 Hz, 1H), 4.93-4.64 (m, 1H), 4.14 (q, *J* = 7.9 Hz, 1H), 2.45-2.31 (m, 1H), 2.22-2.07 (m, 1H), 1.38 (d, *J* = 17.5 Hz, 9H). ^{19}F NMR (376 MHz, DMSO-*d6*) δ -81.18 (d, *J* = 11.2 Hz).

Synthesis of 20c-d

Compound **20c-d** was synthesized by following the general procedure C as described above using compound **19c-d** reacting with TFA salt of compound **3**. The crude product was purified by silica gel column chromatography (PE→CH_2Cl_2: methanol = 15:1) to afford title compound.

20c, off white solid, 30% yield. ^{1}H NMR (400 MHz, DMSO-*d6*) δ 8.33-8.11 (m, 1H), 7.69-7.56 (m, 1H), 7.30 (m, 1H), 7.10-7.01 (m, 1H), 6.75 (td, *J* = 75.5, 3.3 Hz, 1H), 4.86-4.70 (m, 1H), 4.43-4.14 (m, 1H), 3.67-3.41 (m, 1H), 3.25- 2.97 (m, 1H), 2.44-2.11 (m, 2H), 2.10 -1.90 (m, 1H), 1.82-1.42 (m, 1H), 1.43-1.20 (m, 9H). ^{19}F NMR (376 MHz, DMSO-*d6*) δ -81.08 to -80.94 (m).

20d, off white solid, 67% yield. ^{1}H NMR (400 MHz, DMSO-*d6*) δ 8.36-8.17 (m, 1H), 7.68-7.55 (m, 1H), 7.41-7.24 (m, 1H), 7.06 (d, *J* = 6.2 Hz, 1H), 5.12-5.01 (m, 1H), 4.39-4.16 (m, 2H), 3.72-3.52 (m, 2H), 3.23-2.90 (m, 2H), 2.47-2.33 (m, 1H), 2.30-2.17 (m, 3H), 2.04-1.90 (m, 1H), 1.80-1.46 (m, 2H), 1.43-1.26 (m, 9H). ^{19}F NMR (376 MHz, DMSO-*d6*) δ -56.99 (d, *J* = 22.0 Hz).

Synthesis of 21c-d

To a solution of **20c** or **20d** in CH_2Cl_2 was added TFA at 0 °C (CH_2Cl_2:TFA = 1:1, v/v). The solution was stirred for 4 h at room temperature. Then the solution was concentrated. Ethyl ether was added and removed by means of dropper carefully so as not to disturb the precipitate. The residue was concentrated to give TFA salt of **21c**-**d** as light yellow solid

21d, light yellow syrup. ^{1}H NMR (400 MHz, DMSO-*d6*) δ 8.78 (d, *J* = 8.1 Hz, 1H), 7.67 (s, 1H), 7.54 (s, 1H), 7.15 (s, 1H), 5.33-5.24 (m, 1H), 4.52-4.43 (m, 1H), 4.35-4.25 (m, 1H), 3.73-3.65 (m, 1H), 3.56-3.47 (m, 1H), 3.30-3.05 (m, 2H), 2.72-2.62

(m, 1H), 2.40-2.13 (m, 3H), 2.05-1.92 (m, 1H), 1.80-1.64 (m, 1H), 1.64-1.50 (m, 1H). ^{19}F NMR (376 MHz, DMSO-*d6*) δ -57.33 (s), -74.43 (s).

Synthesis of 22a-f

22a-f was synthesized following the general procedure C described above using **21c** or **21d** as start material. The crude product was purified by silica gel column chromatography (PE→CH_2Cl_2: methanol = 15:1) to give title product.

22a, white solid, 71% yield. ^{1}H NMR (400 MHz, DMSO-*d6*) δ 8.28 (d, *J* = 9.0 Hz, 1H), 7.55 (s, 1H), 7.33 (s, 1H), 7.03 (s, 1H), 6.75 (t, *J* = 75.5 Hz, 1H), 6.05 (d, *J* = 9.5 Hz, 1H), 4.92-4.84 (m, 1H), 4.66-4.60 (m, 3H), 4.45-4.37 (m, 1H), 4.34-4.21 (m, 2H), 4.05-4.00 (m, 2H), 3.99-3.88 (m, 3H), 3.85-3.76 (m, 1H), 3.22-2.96 (m, 2H), 2.33-2.09 (m, 2H), 2.07-1.88 (m, 2H),1.72-1.42 (m, 2H), 0.92 (s, 9H).

22b, light yellow solid, 76% yield. ^{1}H NMR (400 MHz, DMSO-*d6*) δ 8.33 (d, *J* = 8.7 Hz, 1H), 7.56 (s, 1H), 7.28 (s, 1H), 7.02 (s, 1H), 6.94 (t, *J* = 75.6 Hz, 1H), 4.95-4.83 (m, 1H), 4.48-4.37 (m, 1H), 4.34-4.15 (m, 1H), 4.04-3.88 (m, 2H), 3.86-3.75 (m, 1H), 3.18-3.03 (m, 2H), 2.34-2.19 (m, 1H), 2.16-1.99 (m, 2H), 1.97-1.88 (m, 1H), 1.78-1.43 (m, 9H), 1.35 (s, 9H), 1.14-1.02 (m, 3H), 0.98-0.82 (m, 2H).

22c, light yellow solid, 63% yield. ^{1}H NMR (400 MHz, DMSO-*d6*) δ 8.31 (d, *J* = 8.6 Hz, 1H), 7.58 (s, 1H), 7.24 (s, 1H), 7.15-7.01 (m, 2H), 6.74 (t, *J* = 75.7 Hz, 1H), 4.96-4.84 (m, 1H), 4.39 (t, *J* = 7.9 Hz, 1H), 4.29-4.17 (m, 1H), 4.09-3.91 (m, 2H), 3.87-3.75 (m, 1H), 3.19-3.01 (m, 2H), 2.33-2.23 (m, 1H), 2.20-2.03 (m, 3H), 1.98-1.88 (m, 1H), 1.70-1.48 (m, 6H), 1.46-1.39 (m,1H), 1.36 (s, 9H), 1.27-1.14 (m, 2H). ^{19}F NMR (376 MHz, DMSO-*d6*) δ -83.11 to -79.68 (m).

22d, off white solid, 60% yield. ^{1}H NMR (400 MHz, DMSO-*d6*) δ 8.35 (d, *J* = 8.8 Hz, 1H), 8.29 (d, *J* = 8.1 Hz, 1H), 7.57 (s, 1H), 7.34 (s, 1H), 7.04 (s, 1H), 5.19-5.08 (m, 1H), 4.47 (t, *J* = 8.1 Hz, 1H), 4.38-4.26 (m, 2H), 4.21-4.15 (m, 1H), 3.91-3.81 (m, 1H), 3.20-3.04 (m, 2H), 2.60-2.52 (m, 1H), 2.44-2.34 (m, 1H), 2.20-2.08 (m, 2H), 2.02-1.91 (m, 1H), 1.90-1.72 (m, 3H), 1.71-1.56 (m, 4H), 1.55-1.45 (m, 1H), 1.38-1.07 (m, 7H), 1.00-0.83 (m, 2H). ^{19}F NMR (376 MHz, DMSO-*d6*) δ -56.98 (s), -195.89 (s).

22e, light yellow solid, 58% yield. ^{1}H NMR (400 MHz, DMSO-*d6*) δ 8.33 (d, *J* = 8.8 Hz, 1H), 8.20 (d, *J* = 8.1 Hz, 1H), 7.57 (s, 1H), 7.31 (s, 1H), 7.03 (s, 1H), 6.74 (t, *J*

= 75.5 Hz, 1H), 4.92-4.85 (m, 1H), 4.44 (t, *J* = 7.8 Hz, 1H), 4.39-4.23 (m, 2H), 4.03-3.93 (m, 1H), 3.87-3.78 (m, 1H), 3.20-3.03 (m, 2H), 2.33-2.21 (m, 1H), 2.19 -2.02 (m, 2H), 1.98-1.89 (m, 1H), 1.87-1.71 (m, 3H), 1.72-1.55 (m, 4H), 1.53-1.45 (m, 1H), 1.36-1.20 (m, 3H), 1.22-1.06 (m, 5H), 0.99-0.81 (m, 2H). ^{19}F NMR (376 MHz, DMSO-*d*6) δ -83.12 to -78.83 (m), -195.85 (s).

22f, light yellow solid, 66% yield. ^{1}H NMR (400 MHz, DMSO-*d*6) δ 8.38-8.22 (m, 1H), 7.69-7.55 (m, 1H), 7.28-7.16 (m, 1H), 7.09-7.00 (m, 1H), 6.96-6.53 (m, 2H), 4.94-4.86 (m, 1H), 4.82-4.74 (m, 1H), 4.63-4.51 (m, 1H), 4.47-4.36 (m, 1H), 4.27-4.15 (m, 1H), 3.89-3.76 (m, 2H), 3.57-3.39 (m, 2H), 3.20-3.02 (m, 2H), 2.43-2.24 (m, 3H), 2.21-2.00 (m, 3H), 1.97-1.86 (m, 1H), 1.71-1.46 (m, 2H), 1.43-1.24 (m, 9H). ^{19}F NMR (376 MHz, DMSO-*d*6) δ -82.18 to -80.46 (m).

(*S*)-3,3-dimethyl-2-(2-oxa-6-azaspiro[3.3]heptane-6-carboxamido)butanoic acid (25)

Compound **23** (529 mg, 2.9 mmol, 1 equiv) was dissolved in ACN (10 mL) and cooled to 0 °C. Triethylamine (884 mg, 8.8 mol, 3 equiv) and CDI (520 mg, 3.2 mmol, 1.1 equiv) was added. The resulting reaction solution was stirred at 0 °C for 2 h. Then 2-oxa-6-azaspiro[3.3]heptane (840 mg, 2.9 mmol, 1 equiv) was added. The resulting solution was stirred at room temperature for 5h. Then EtOAc and water was added. The organic layer was separated and washed with brine, dried and concentrated under vacuum. The crude product was purified by silica gel column chromatography eluted with 45% EtOAc/petroleum ether to afford compound **24** as light yellow oil (700 mg, 90% yield). ^{1}H NMR (400 MHz, $CDCl_3$) δ 4.78 (s, 1H), 4.63 (d, *J* = 9.4 Hz, 1H), 4.32-4.24 (m, 1H), 4.20 - 4.08 (m, 1H), 3.71 (m, 3H), 0.96 (s, 9H).

Compound **25** was synthesized following the general procedure B described above using **24** as start material. The crude was used directly for the next step.

(*S*)-2-cyclohexyl-2-(1-fluorocyclopropane-1-carboxamido)acetic acid (28)

Compound **27** was synthesized by following the general procedure A as described above using compound **26** reacting with 1-fluoro-cyclopropanecarboxylic acid. The crude product was purified by silica gel column chromatography eluted with 40% EtOAc/petroleum ether to afford compound **27** as light yellow oil (960 mg, 96% yield).

^{1}H NMR (400 MHz, $CDCl_3$) δ 6.97-6.79 (m, 1H), 4.63-4.55 (m, 1H), 3.75 (s, 3H), 1.94 -1.60 (m, 5H), 1.50- 0.91 (m, 10H). ^{19}F NMR (376 MHz, $CDCl_3$) δ -197.62 (s).

Compound **28** was synthesized following the general procedure B described above using **27** as start material. The crude was used directly for the next step.

Synthesis of H129-H131, H135, H136, H141

H129-H131, **H135**, **H136, H141** was prepared following the general procedure D described above using **22a-f** as start material.

H129, white solid, 34% yield. ^{1}H NMR (400 MHz, DMSO-*d6*) δ 9.02 (d, J = 8.7 Hz, 1H), 7.66 (s, 1H), 6.76 (t, J = 75.4 Hz, 1H), 6.06 (d, J = 9.4 Hz, 1H), 5.02-4.79 (m, 2H), 4.62 (s, 4H), 4.32-4.19 (m, 2H), 4.06-3.99 (m, 2H), 3,98-3.90 (m, 3H), 3.85-3.77 (m, 1H), 3.19-3.10 (m, 1H), 3.10-2.96 (m, 1H), 2.34-2.23(m, 1H), 2.21-1.94 (m, 3H), 1.81-1.59 (m, 2H), 0.89 (s, 9H). ^{19}F NMR (376 MHz, DMSO-*d6*) δ -80.51 – -81.79 (m). ^{13}C NMR (101 MHz, DMSO-*d6*) δ 178.12, 171.38, 170.87, 159.62, 120.10, 117.38 (t, J = 255.9 Hz), 80.30, 75.05, 59.08, 58.41, 57.91, 54.12, 38.10, 37.66, 37.09, 35.80, 35.30, 34.87, 27.32, 26.73. MS (ESI) calcd for $C_{25}H_{37}F_2N_6O_6$ $[M + H]^+$555.2743, found 555.2745. HPLC purity: 97.27%.

H130, white solid, 53% yield. ^{1}H NMR (400 MHz, DMSO-*d6*) δ 9.01 (d, J = 8.5 Hz, 1H), 7.67 (s, 1H), 6.96 (d, J = 8.8 Hz, 1H), 6.66 (t, J = 75.6 Hz, 1H), 5.09-4.83 (m, 2H), 4.29 (t, J = 8.1 Hz, 1H), 4.09-3.90 (m, 2H), 3.85-3.73 (m, 1H), 3.25-2.98 (m, 2H), 2.62-2.53 (m, 1H), 2.35-2.24 (m, 1H), 2.23-2.12 (m, 1H), 2.10-1.99 (m, 2H), 1.82-1.51 (m, 8H), 0.93 (s, 9H), 1.17-1.03 (m, 3H), 0.98-0.83 (m, 2H). ^{19}F NMR (376 MHz, DMSO-*d6*) δ -81.96 to -80.56 (m). ^{13}C NMR (101 MHz, DMSO-*d6*) δ 178.19, 171.48, 171.01, 156.05, 120.07, 117.37 (t, J = 255.6 Hz), 78.46, 75.05, 58.37, 56.90, 53.24, 38.24, 37.22, 35.68, 34.44, 29.07, 28.59, 27.54, 26.35, 26.06, 25.98. MS (ESI) calcd for $C_{26}H_{40}F_2N_5O_6$ $[M + H]^+$ 556.2947, found 556.2947. HPLC purity: 98.46%.

H131, white solid, 38% yield. ^{1}H NMR (400 MHz, DMSO-*d6*) δ 8.98 (d, J = 8.4 Hz, 1H), 7.66 (s, 1H), 7.10 (d, J = 8.3 Hz, 1H), 6.75 (t, J = 75.7 Hz, 1H), 5.20-4.72 (m, 2H), 4.30 (t, J = 8.0 Hz, 1H), 4.14-3.91 (m, 2H), 3.85-3.62 (m, 1H), 3.21-3.01 (m, 2H), 2.59-2.52 (m, 1H), 2.35-2.23 (m, 1H), 2.21-1.97 (m, 4H), 1.81-1.49 (m, 6H), 1.48-1.40 (m, 1H), 1.36 (s, 9H), 1.28-1.11 (m, 2H). ^{19}F NMR (376 MHz, DMSO-*d6*) δ -81.91 to

-80.70 (m). ^{13}C NMR (101 MHz, DMSO-*d*6) δ 178.15, 171.44, 171.29, 156.03, 120.05, 117.35 (t, *J* = 255.6 Hz), 78.53, 75.00, 58.35, 56.03, 53.15, 41.94, 38.31, 37.23, 35.66, 34.40, 29.06, 28.79, 28.59, 27.56, 25.40, 24.89. MS (ESI) calcd for $C_{25}H_{38}F_2N_5O_6$ [M + H]$^+$ 542.2790, found 556.2784. HPLC purity: 99.19%.

H136, white solid, 60% yield. ^{1}H NMR (400 MHz, DMSO-*d*6) δ 9.03 (d, *J* = 8.5 Hz, 1H), 8.22 (d, *J* = 8.0 Hz, 1H), 7.68 (s, 1H), 6.75 (t, *J* = 75.5 Hz, 1H), 5.03-4.94 (m, 1H), 4.93-4.82 (m, 1H), 4.42-4.26 (m, 2H), 4.08-3.99 (m, 1H), 3.86-3.75 (m, 1H), 3.21-2.99 (m, 2H), 2.61-2.52 (m, 1H), 2.36-2.25 (m, 1H), 2.22-2.00 (m, 3H), 1.90-1.51 (m, 8H), 1.39-1.04 (m, 7H), 1.00-0.82 (m, 2H). ^{19}F NMR (376 MHz, DMSO) δ -86.31 to -76.06 (m), -195.92 (s). ^{13}C NMR (101 MHz, DMSO-*d*6) δ 178.21, 171.33, 170.14, 169.19 (d, *J* = 21.3 Hz), 120.05, 117.35 (t, *J* = 255.9 Hz), 77.89 (d, *J* = 232.8 Hz). 74.92, 58.45, 55.72, 53.37, 38.32, 37.27, 35.68, 34.39, 28.95, 28.76, 27.52, 26.28, 26.04, 25.88, 13.28 (d, *J* = 10.7 Hz). MS (ESI) calcd for $C_{25}H_{35}F_3N_5O_5$ [M + H]$^+$ 542.2590, found 542.2592. HPLC purity: 95.15%.

H135, white solid, 62% yield. ^{1}H NMR (400 MHz, DMSO-*d*6) δ 9.03 (d, *J* = 8.5 Hz, 1H), 8.29 (d, *J* = 7.9 Hz, 1H), 7.67 (s, 1H), 5.21-5.10 (m, 1H), 5.06-4.89 (m, 1H), 4.40-4.26 (m, 2H), 4.21 (d, *J* = 12.0 Hz, 1H), 3.95-3.81 (m, 1H), 3.21-3.05 (m, 2H), 2.60-2.52 (m, 1H), 2.45-2.35 (m, 1H), 2.24-1.99 (m, 3H), 1.91-1.53 (m, 8H), 1.37-1.04 (m, 7H), 1.00-0.82 (m, 2H). ^{13}C NMR (101 MHz, DMSO-*d*6) δ 178.13, 171.05, 170.20, 169.22 (d, *J* = 21.3 Hz), 121.59 (q, *J* = 254.8 Hz), 119.99, 78.66, 77.84 (d, *J* = 233.7 Hz), 58.24, 55.82, 53.31, 38.37, 37.26, 35.47, 34.42, 28.93, 28.81, 27.52, 26.29, 26.02, 25.88, 13.28 (d, *J* = 5.8 Hz).^{19}F NMR (376 MHz, DMSO-*d*6) δ -56.99 (s), -201.16 to -189.93 (m).MS (ESI) calcd for $C_{25}H_{34}F_4N_5O_5$ [M + H]$^+$ 560.2496, found 560.2453. HPLC purity: 97.79%.

H141, off white solid, 54% yield. ^{1}H NMR (400 MHz, DMSO-*d*6) δ 9.00-8.90 (m, 1H), 7.71 (s, 1H), 7.09-6.43 (m, 2H), 5.07-4.88 (m, 2H), 4.81-4.73 (m, 1H), 4.60-4.49 (m, 1H), 4.41-4.26 (m, 1H), 4.01-3.70 (m, 2H), 3.55-3.40 (m, 2H), 3.22-2.99 (m, 2H), 2.46 -2.23 (m, 3H), 2.19-1.92 (m, 4H), 1.80-1.50 (m, 2H), 1.35 (d, *J* = 25.5 Hz, 9H).^{19}F NMR (376 MHz, DMSO-*d*6) δ -82.13 to -80.61 (m). MS (ESI) calcd for $C_{24}H_{33}F_4N_5NaO_7$ [M + H]$^+$ 602.2214, found 602.2216. HPLC purity: 99.80%.

Table S1. Diffraction data and refinement statistics

*Values in parentheses are for highest-resolution shel

	M^{pro} H109
**PDB Code**	9IK2
**Data collection**	
Space group	P622
Cell dimensions	
a, *b*, *c* (Å)	106.22, 106.22, 82.38
α, β, γ (°)	90, 90, 120
Wavelength (Å)	0.979
Resolution (Å)	50-1.80 (1.80-1.86)
R_{merge}	0.108 (0.872)
$I / \sigma I$	185.2 / 3.9 (15.5 / 2.4)
CC1/2	0.954 (0.955)
Completeness (%)	99.9 (100.0)
Redundancy	29.5 (24.7)
**Refinement**	
Resolution (Å)	37.59-1.80
No. reflections	25943
R_{work} / R_{free}	0.1832/0.2118
No. atoms	
Protein	2353
Ligand/ion	37
Water	237
B-factors	
Protein	22.6
Ligand/ion	19.2
Water	33.3
R.m.s. deviations	
Bond lengths (Å)	0.003
Bond angles (°)	0.689
Ramachandran plot	
Favored (%)	97.68
Allowed (%)	2.32
Outliers (%)	0

^{1}H NMR, ^{13}C NMR, HPLC, HRMS spectra of target compounds

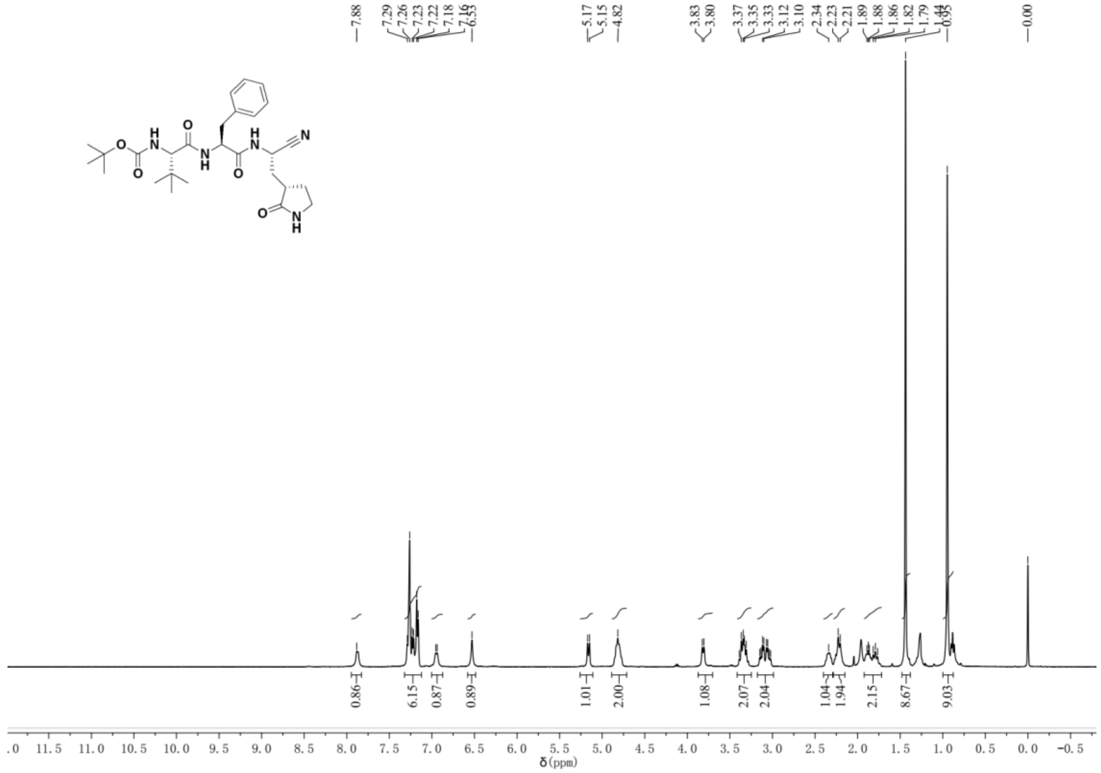

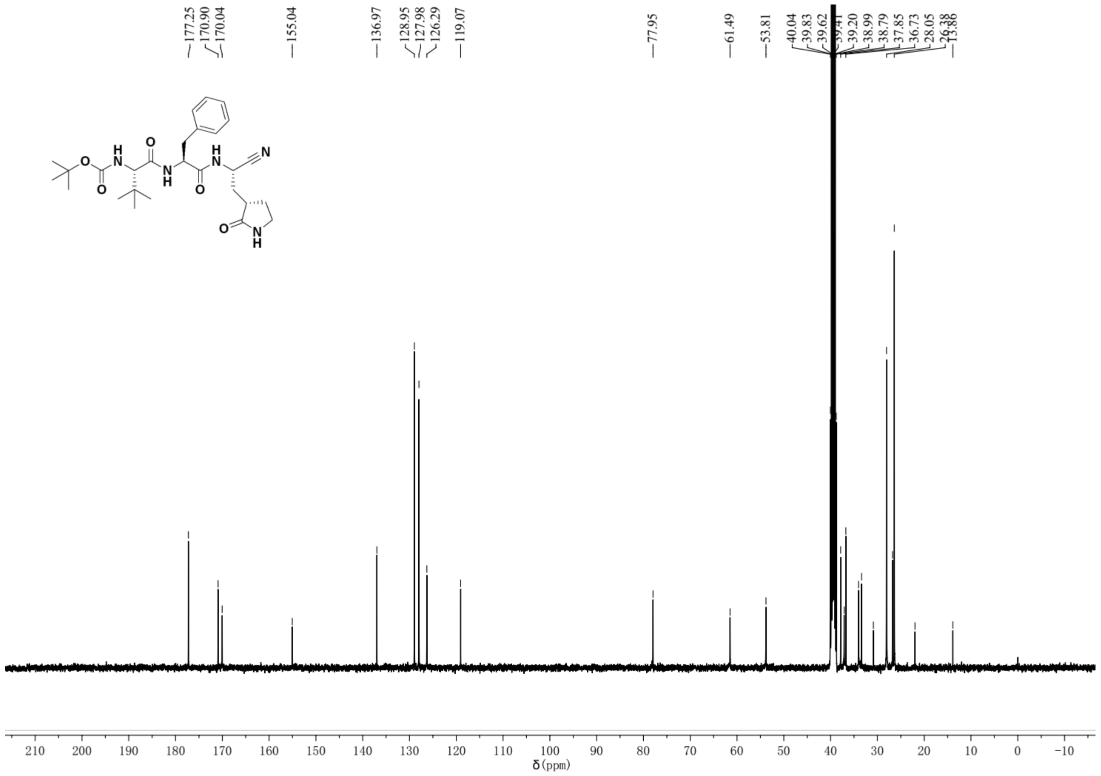

^{1}H NMR and ^{13}C NMR of **H109**

SHIMADZU LabSolutions

Analysis Report

<Sample Information>

Sample Name :
Sample ID :
Data Filename : HW210705-0-2-88A6.lcd
Method Filename : proteasome intermediate.lcm
Batch Filename :
Vial # : 0
Sample Type : Unknown
Injection Volume : 5 uL
Date Acquired : 10/8/2021 10:56:27 AM
Acquired by : System Administrator
Date Processed : 8/10/2022 9:07:39 PM
Processed by : System Administrator

<Chromatogram>

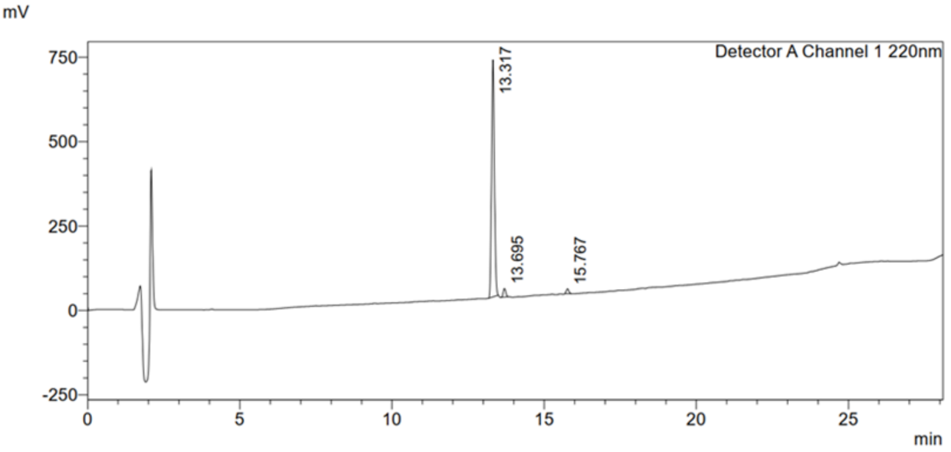

Peak#	Ret. Time	Area	Height	Conc.	Area%	Height%
1	13.317	4553493	702480	95.160	95.160	94.660
2	13.695	145258	24975	3.036	3.036	3.365
3	15.767	86362	14652	1.805	1.805	1.974
Total		4785113	742107		100.000	100.000

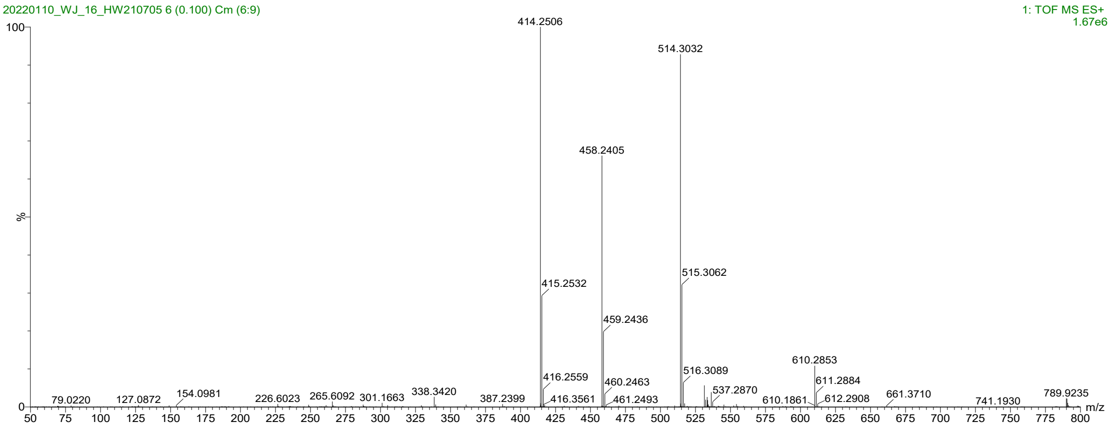

HPLC and HRMS of **H109**

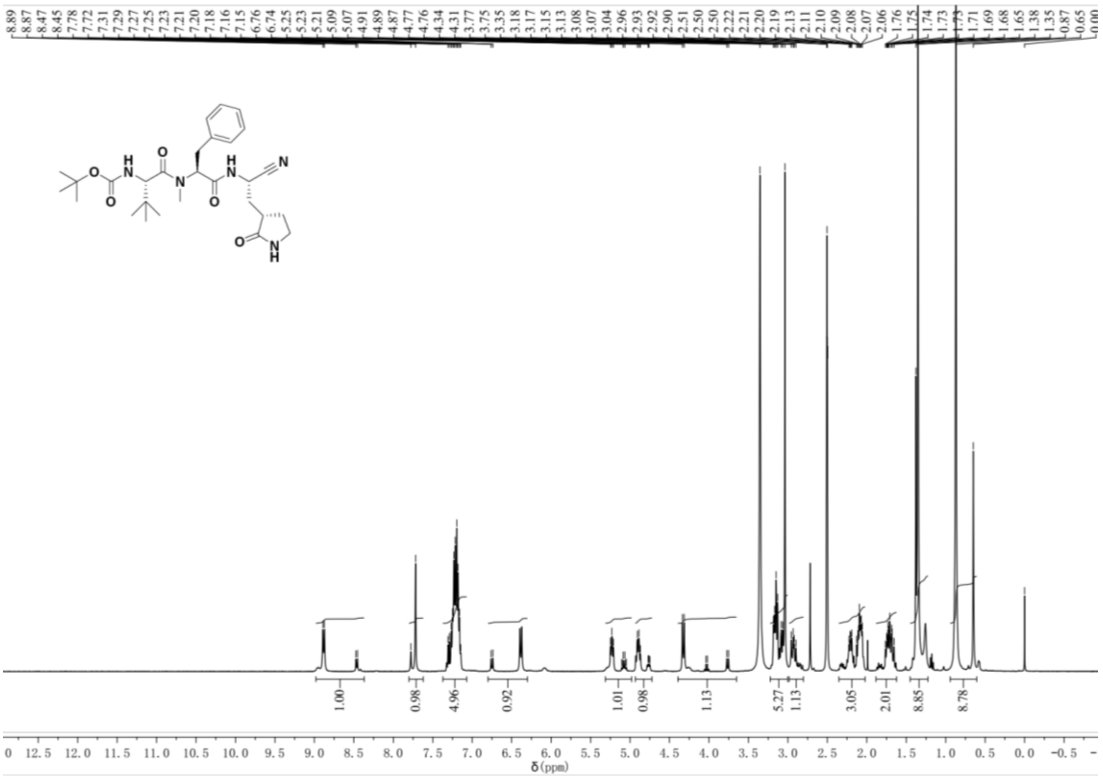

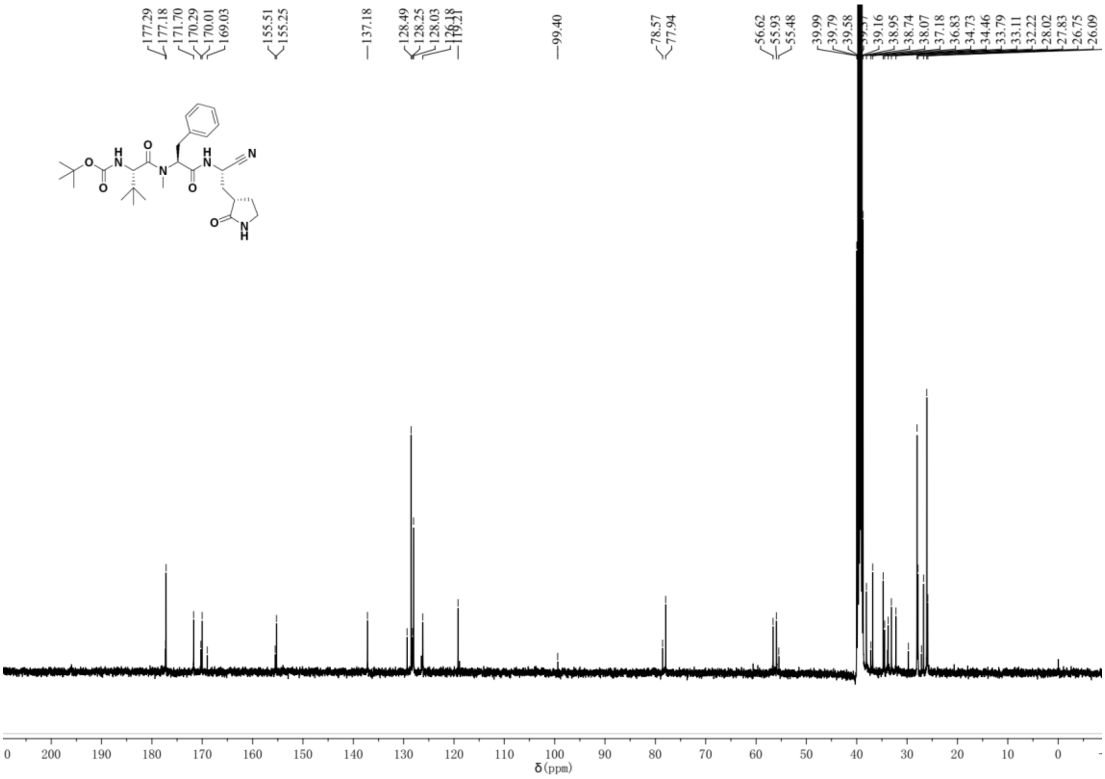

^{1}H NMR and ^{13}C NMR of **H119**

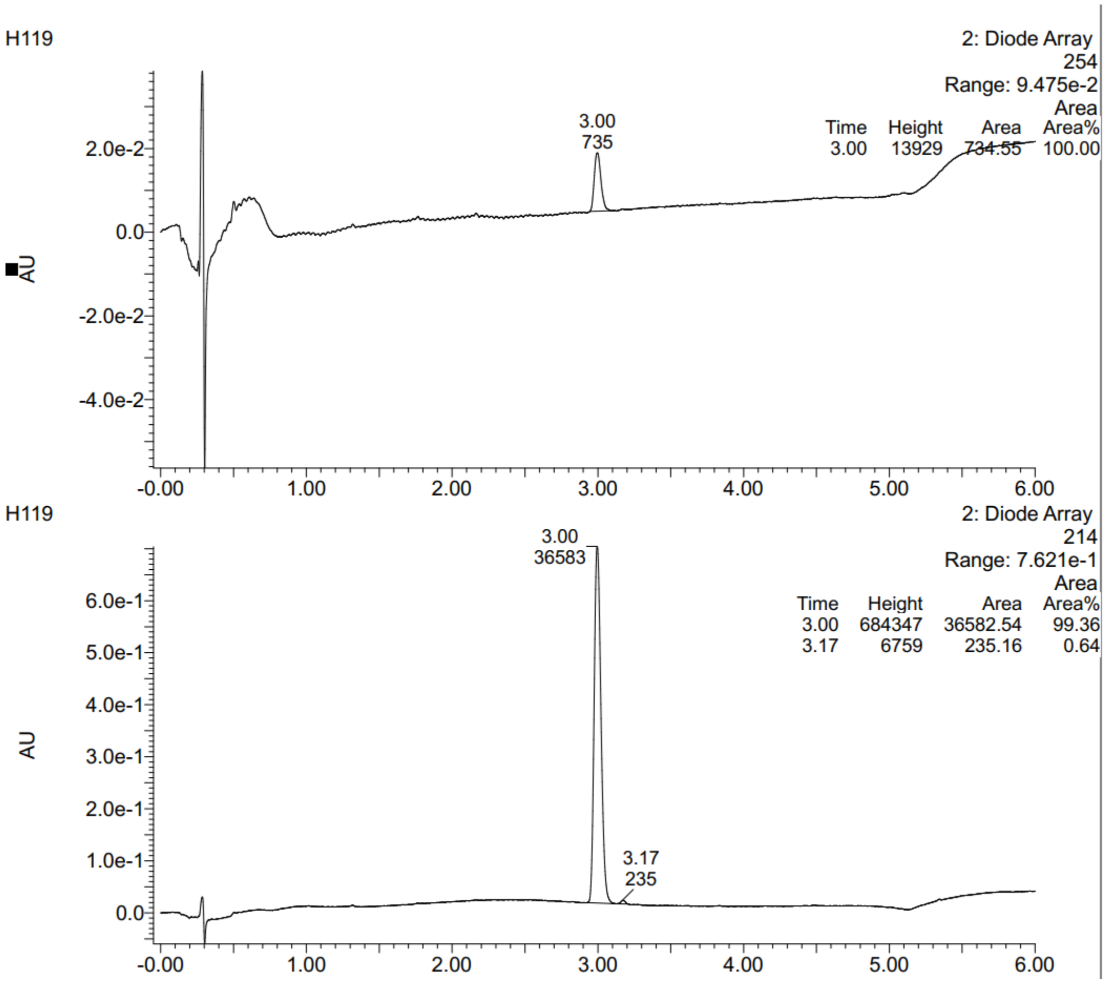

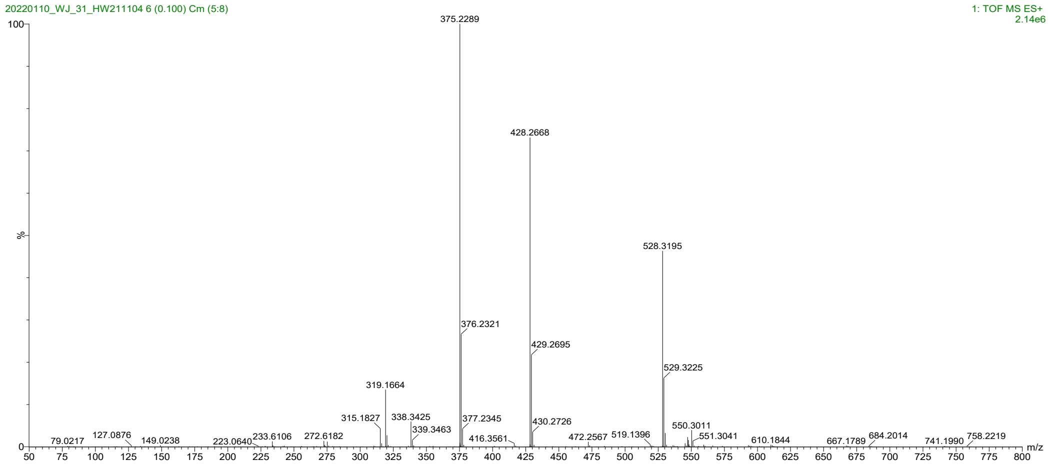

HPLC and HRMS of **H119**

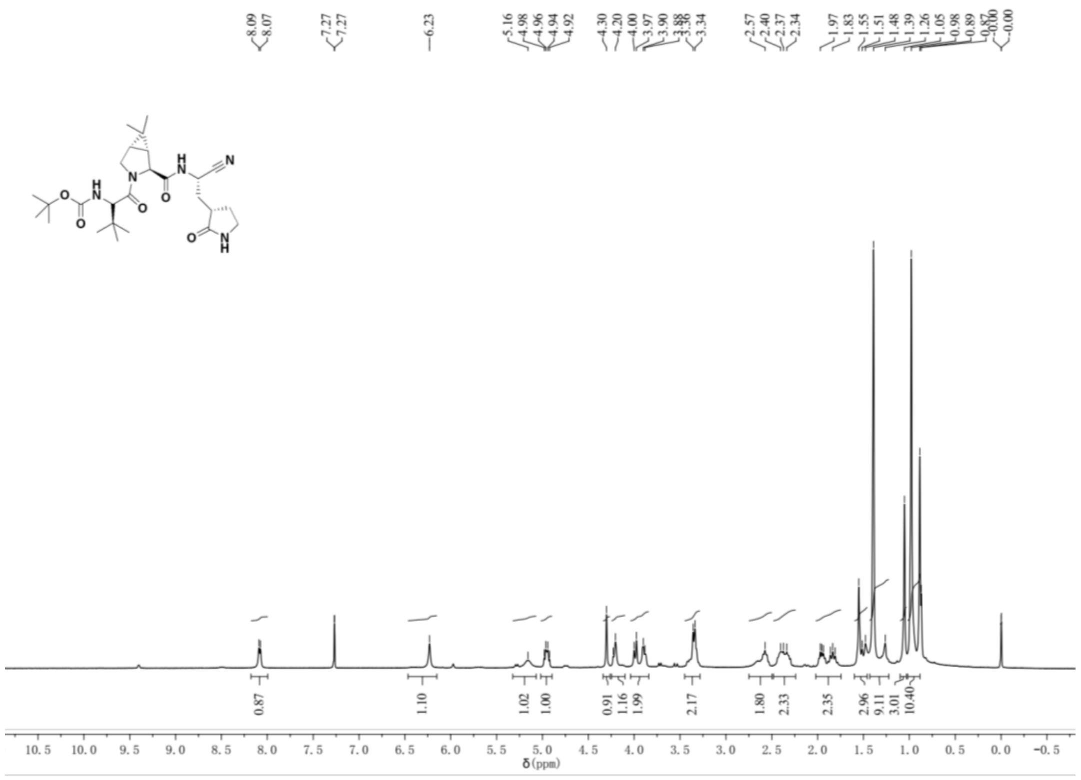

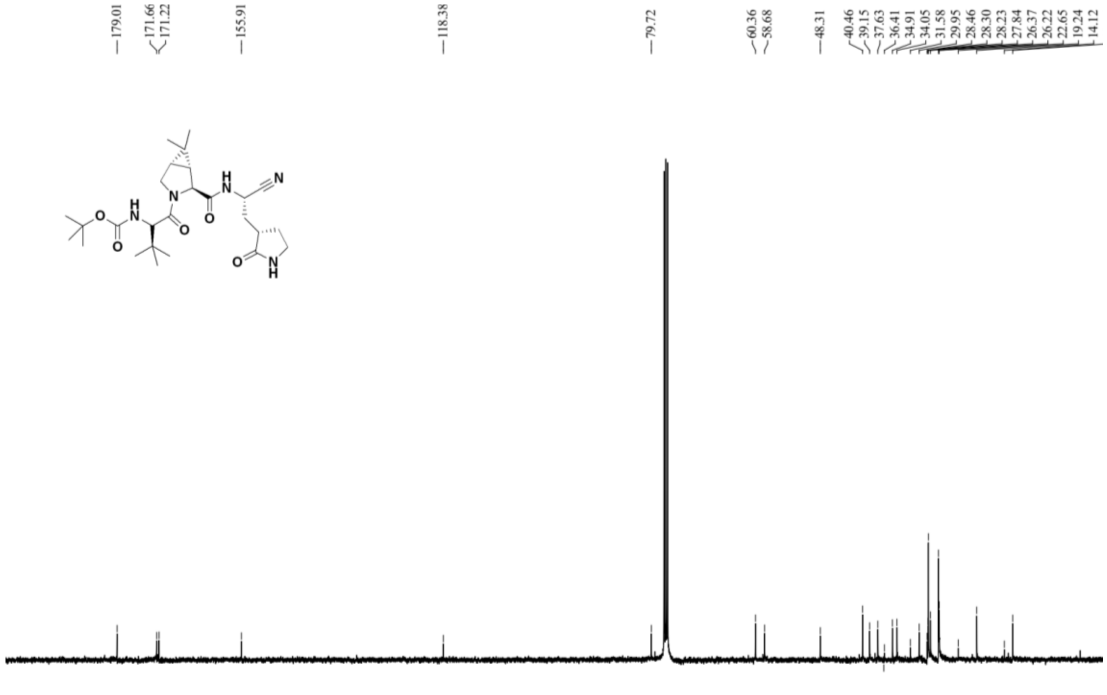

^{1}H NMR and ^{13}C NMR of **H116**

SHIMADZU LabSolutions

Analysis Report

<Sample Information>

Sample Name	:		
Sample ID	:		
Data Filename	: HW211009-0-1-95A`.lcd		
Method Filename	: proteasome intermediate.lcm		
Batch Filename	:		
Vial #	: 0	Sample Type	: Unknown
Injection Volume	: 5 uL		
Date Acquired	: 10/20/2021 2:57:11 PM	Acquired by	: System Administrator
Date Processed	: 10/20/2021 3:25:19 PM	Processed by	: System Administrator

<Chromatogram>

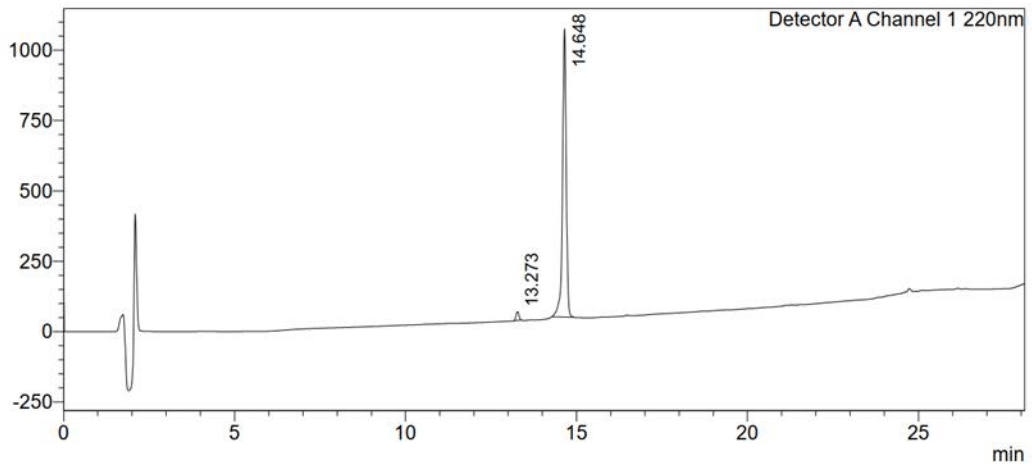

Peak#	Ret. Time	Area	Height	Area%	Height%	Conc.
1	13.273	177069	30186	2.245	2.862	2.245
2	14.648	7711326	1024507	97.755	97.138	97.755
Total		7888395	1054693	100.000	100.000	

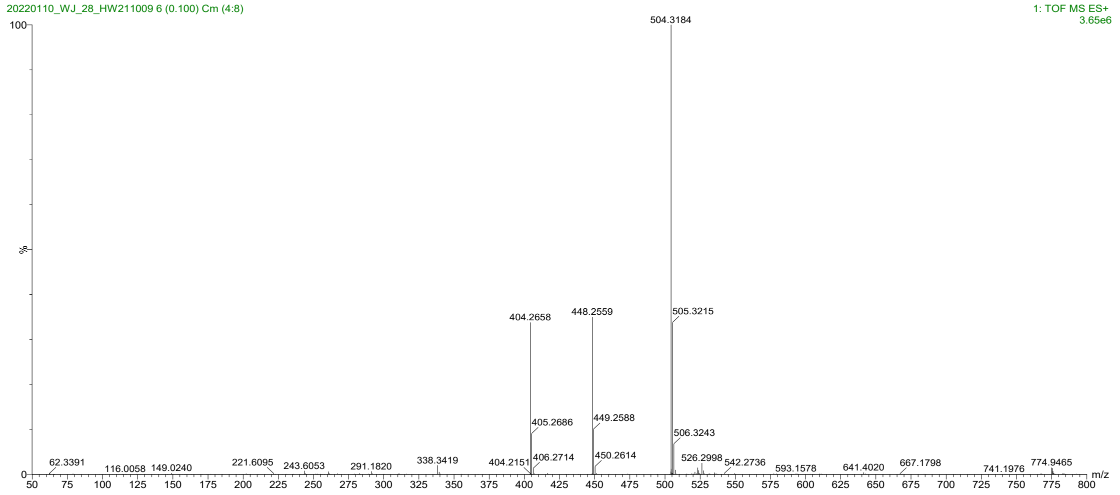

HPLC and HRMS of **H116**

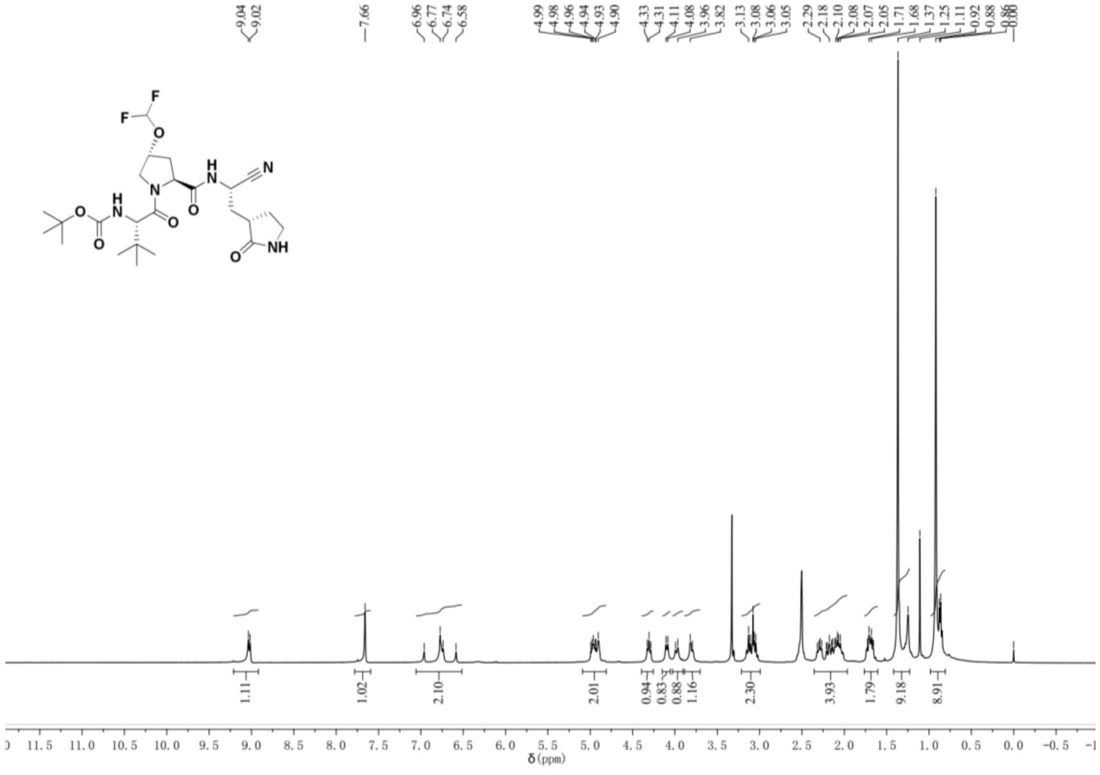

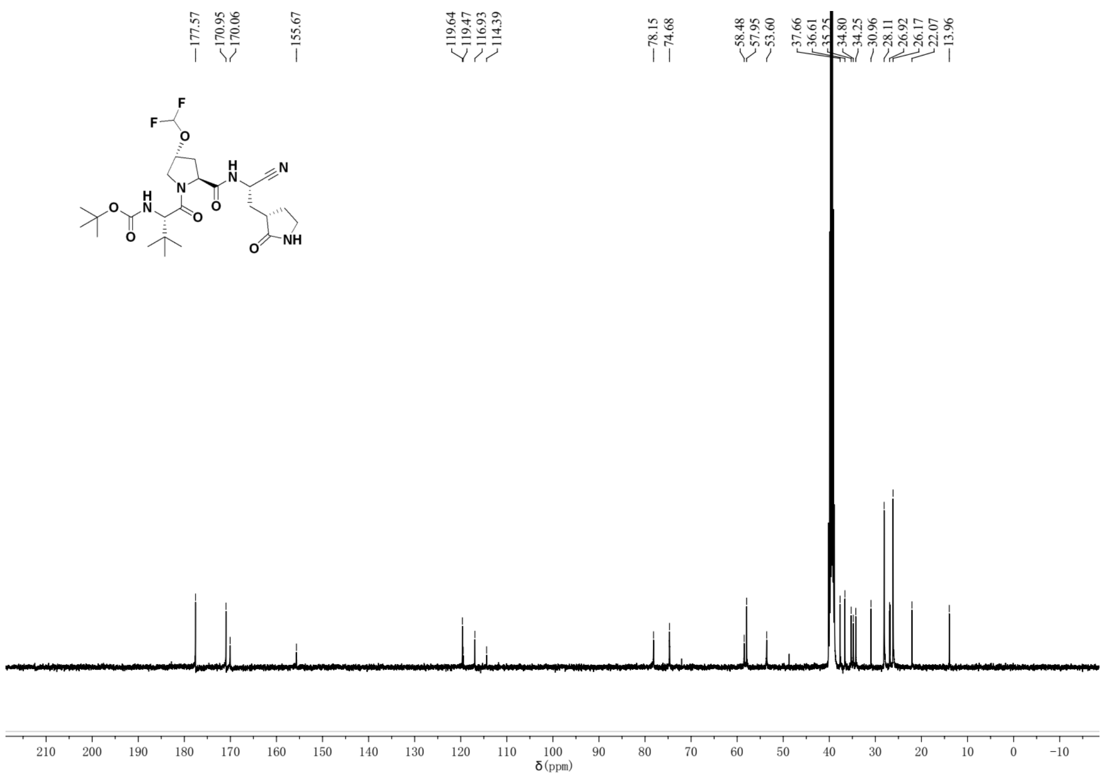

^{1}H NMR and ^{13}C NMR of **H117**

SHIMADZU LabSolutions

Analysis Report

<Sample Information>

Sample Name	:		
Sample ID	:		
Data Filename	: HW211021-0-1-106A1.lcd		
Method Filename	: proteasome intermediate.lcm		
Batch Filename	:		
Vial #	: 0	Sample Type	: Unknown
Injection Volume	: 5 uL		
Date Acquired	: 11/8/2021 7:25:32 PM	Acquired by	: System Administrator
Date Processed	: 11/8/2021 7:53:39 PM	Processed by	: System Administrator

<Chromatogram>

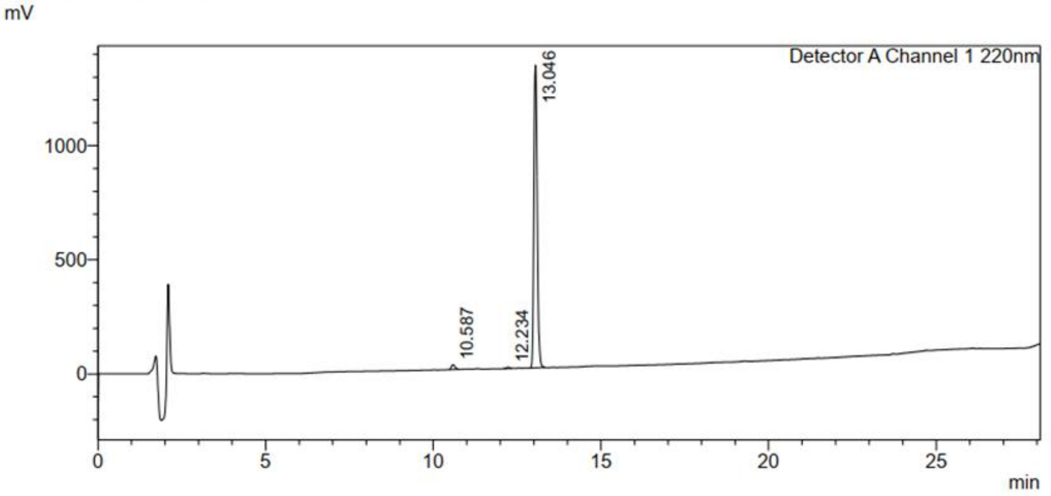

Peak#	Ret. Time	Area	Height	Area%	Height%	Conc.
1	10.587	152405	20825	1.638	1.543	1.638
2	12.234	31260	4961	0.336	0.368	0.336
3	13.046	9123451	1323468	98.027	98.089	98.027
Total		9307117	1349254	100.000	100.000	

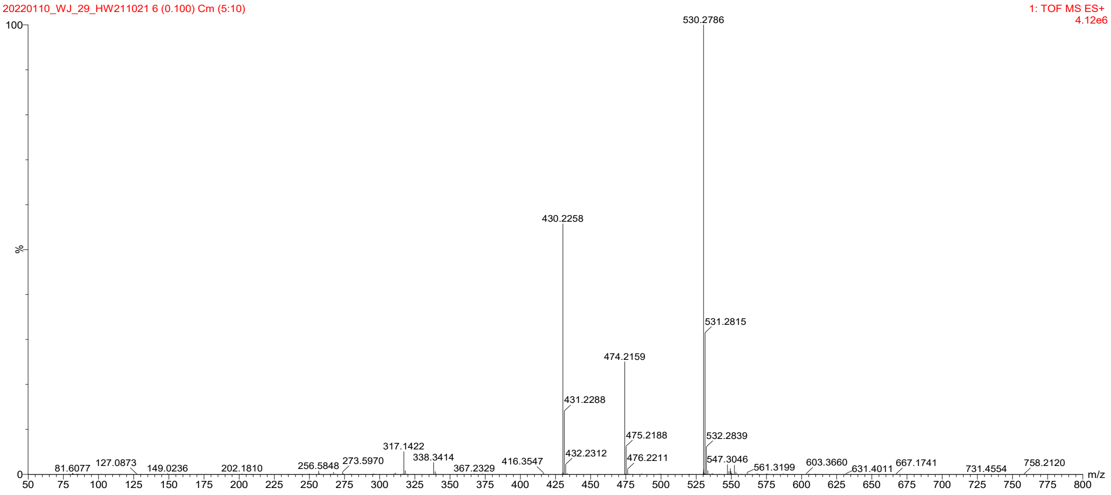

HPLC and HRMS of **H117**

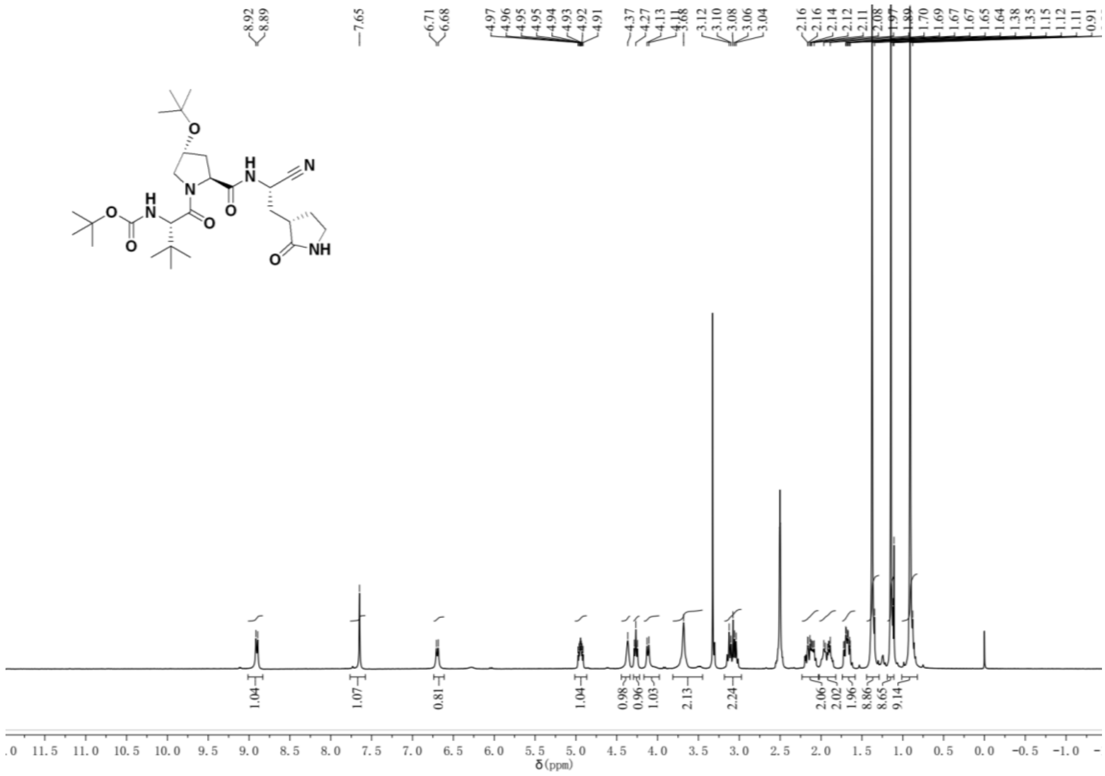

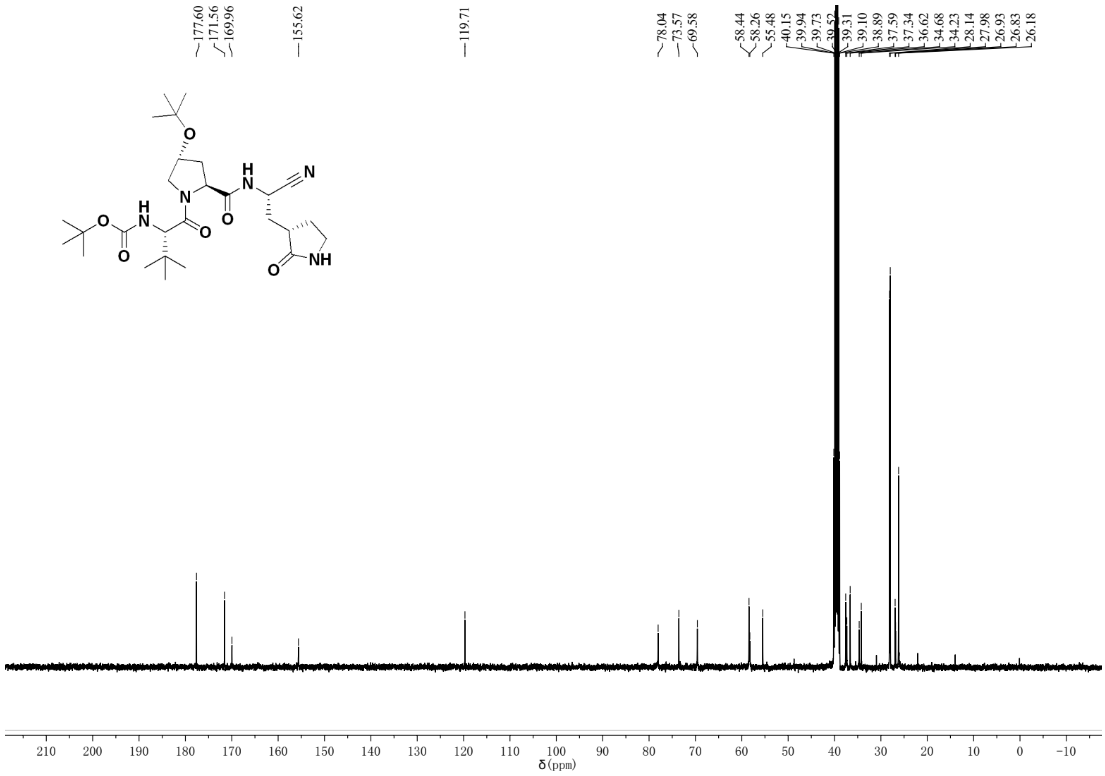

^{1}H NMR and ^{13}C NMR of **H118**

SHIMADZU LabSolutions

Analysis Report

<Sample Information>

Sample Name	:		
Sample ID	:		
Data Filename	: HW211101-0-1-114A2.lcd		
Method Filename	: proteasome intermediate.lcm		
Batch Filename	:		
Vial #	: 0	Sample Type	: Unknown
Injection Volume	: 8 uL		
Date Acquired	: 11/14/2021 8:03:45 PM	Acquired by	: System Administrator
Date Processed	: 11/14/2021 8:31:52 PM	Processed by	: System Administrator

<Chromatogram>

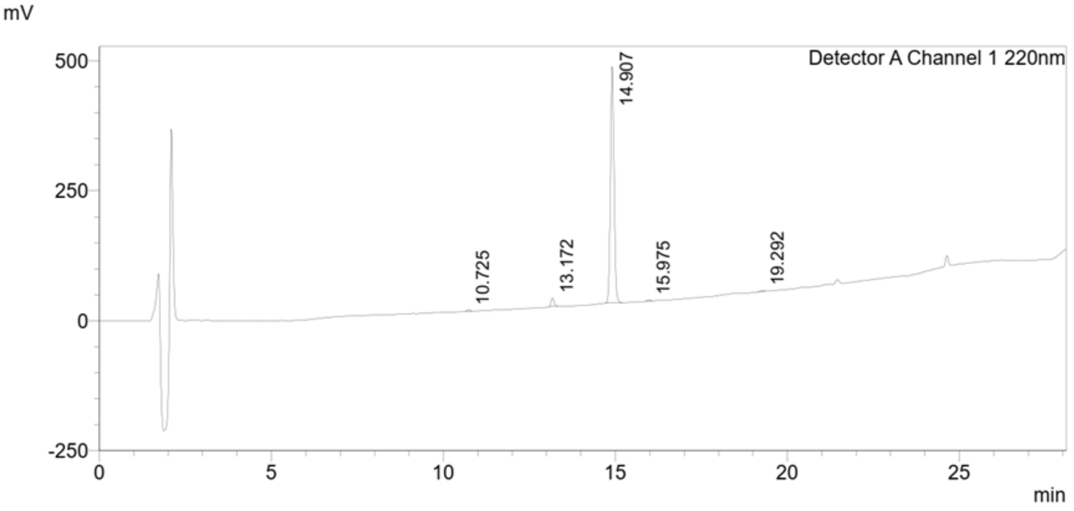

Peak#	Ret. Time	Area	Height	Area%	Height%	Conc.
1	10.725	17433	3195	0.509	0.669	0.509
2	13.172	97517	16361	2.848	3.427	2.848
3	14.907	3284874	453877	95.940	95.076	95.940
4	15.975	13774	2382	0.402	0.499	0.402
5	19.292	10295	1569	0.301	0.329	0.301
Total		3423894	477384	100.000	100.000	

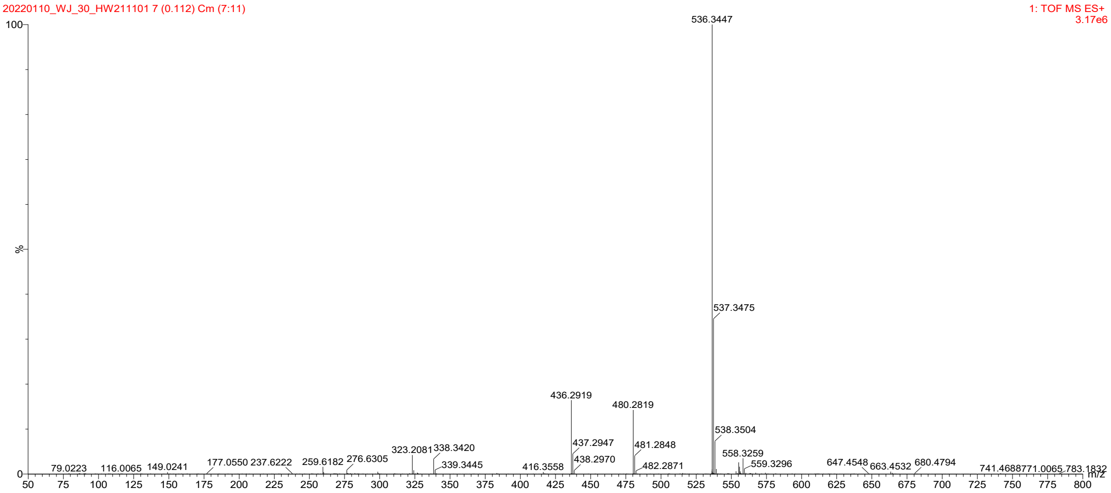

HPLC and HRMS of **H118**

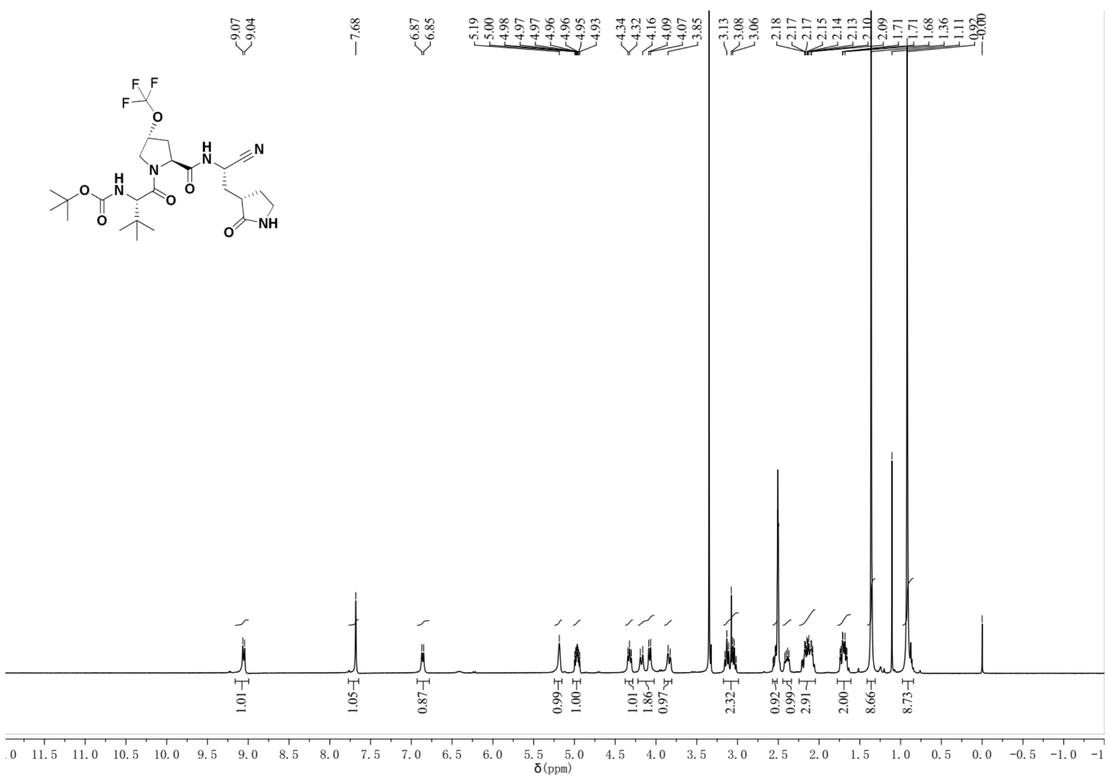

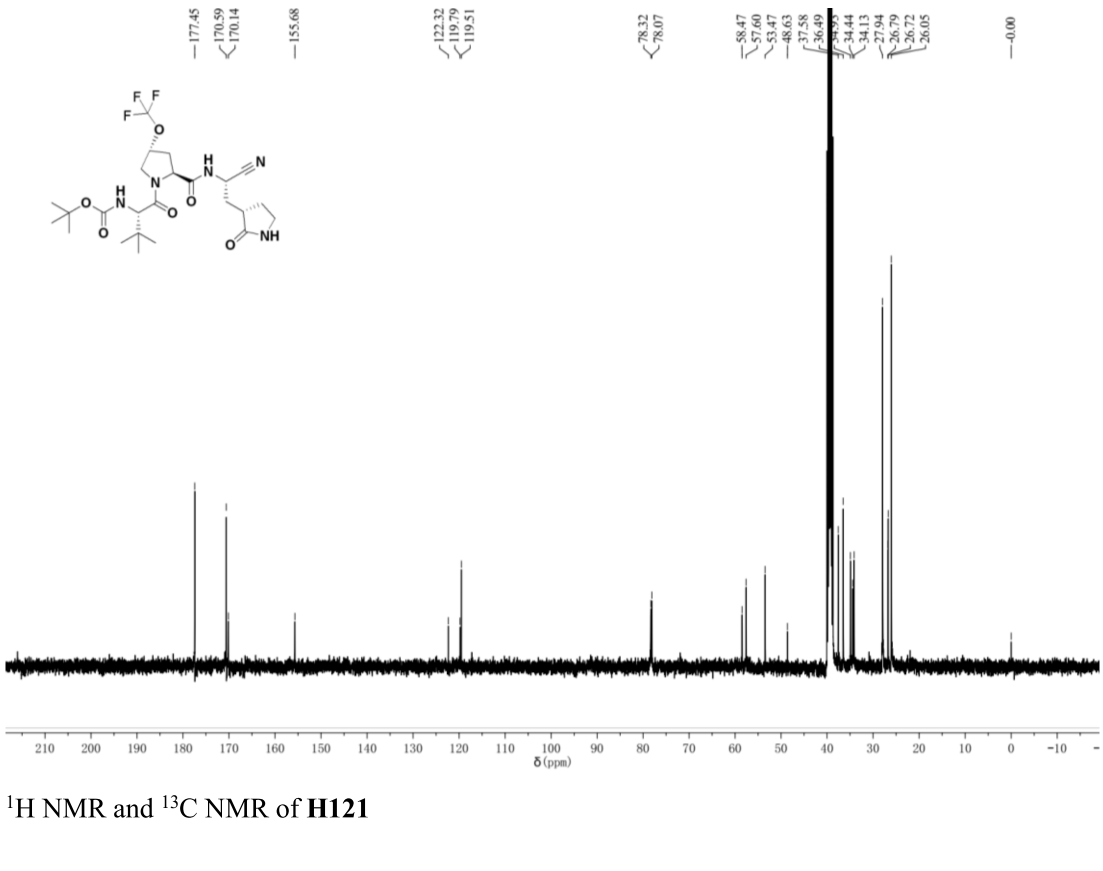

^{1}H NMR and ^{13}C NMR of **H121**

SHIMADZU LabSolutions Analysis Report

<Sample Information>

Sample Name :
Sample ID :
Data Filename : HW211122-0-1-134A2.lcd
Method Filename : proteasome intermediate.lcm
Batch Filename :
Vial # : 0
Sample Type : Unknown
Injection Volume : 10 uL
Date Acquired : 12/2/2021 3:37:18 PM
Acquired by : System Administrator
Date Processed : 12/2/2021 4:05:26 PM
Processed by : System Administrator

<Chromatogram>

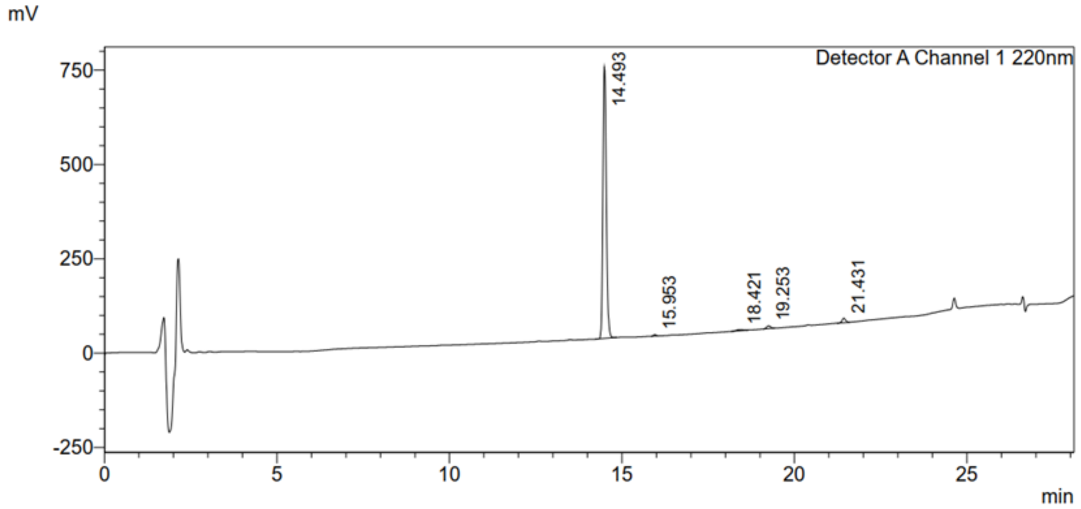

Peak#	Ret. Time	Area	Height	Area%	Height%	Conc.
1	14.493	4908346	718835	95.889	96.541	95.889
2	15.953	22937	3594	0.448	0.483	0.448
3	18.421	44278	3079	0.865	0.414	0.865
4	19.253	61841	7277	1.208	0.977	1.208
5	21.431	81367	11805	1.590	1.585	1.590
Total		5118769	744590	100.000	100.000	

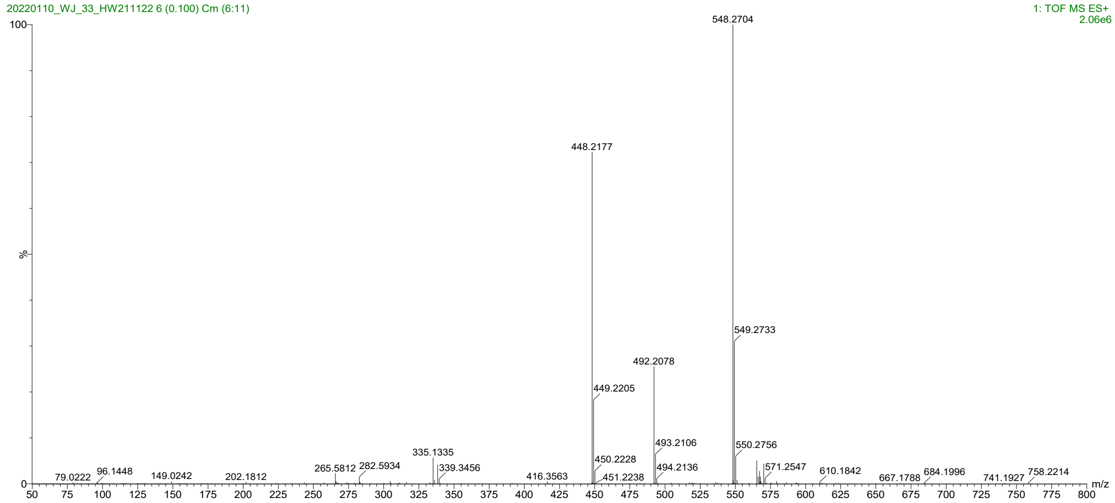

HPLC and HRMS of **H121**

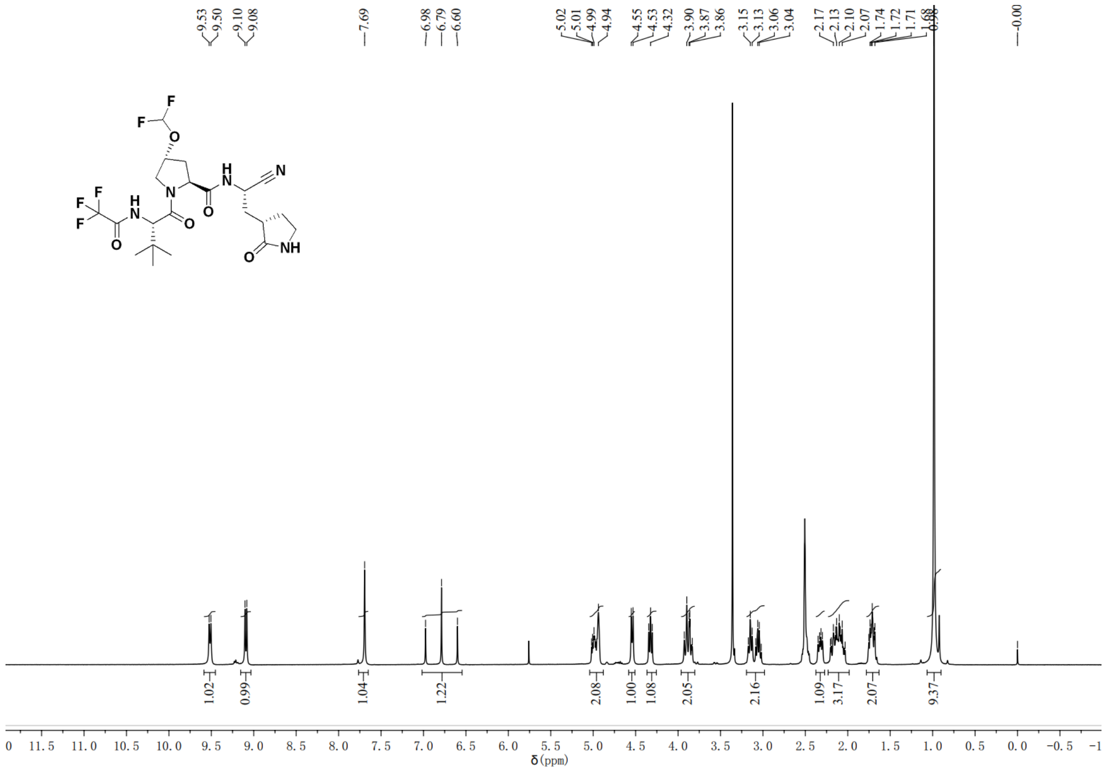

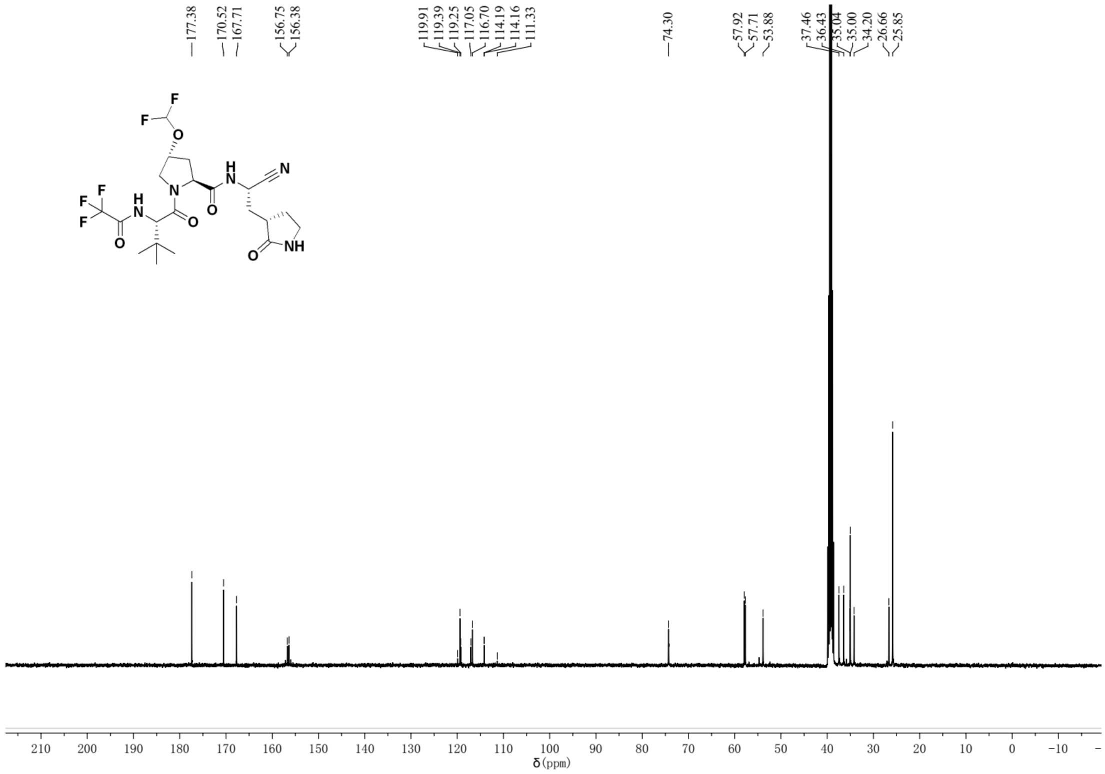

^{1}H NMR and ^{13}C NMR of **H122**

SHIMADZU LabSolutions

Analysis Report

<Sample Information>

Sample Name	:		
Sample ID	:		
Data Filename	: HW211201-0-1-145A1.lcd		
Method Filename	: proteasome intermediate.lcm		
Batch Filename	:		
Vial #	:	Sample Type	: Unknown
Injection Volume	: 5 uL		
Date Acquired	: 8/22/2022 9:43:36 AM	Acquired by	: System Administrator
Date Processed	: 8/22/2022 10:11:44 AM	Processed by	: System Administrator

<Chromatogram>

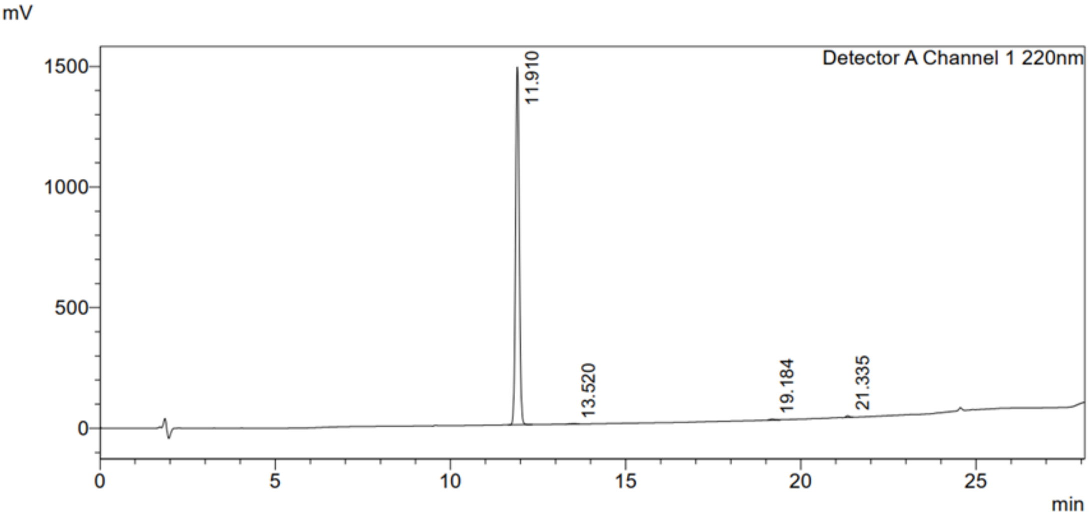

Peak#	Ret. Time	Area	Height	Area%	Height%	Conc.
1	11.910	10795046	1482314	99.132	99.178	99.132
2	13.520	22046	1699	0.202	0.114	0.202
3	19.184	29911	3891	0.275	0.260	0.275
4	21.335	42544	6702	0.391	0.448	0.391
Total		10889547	1494606	100.000	100.000	

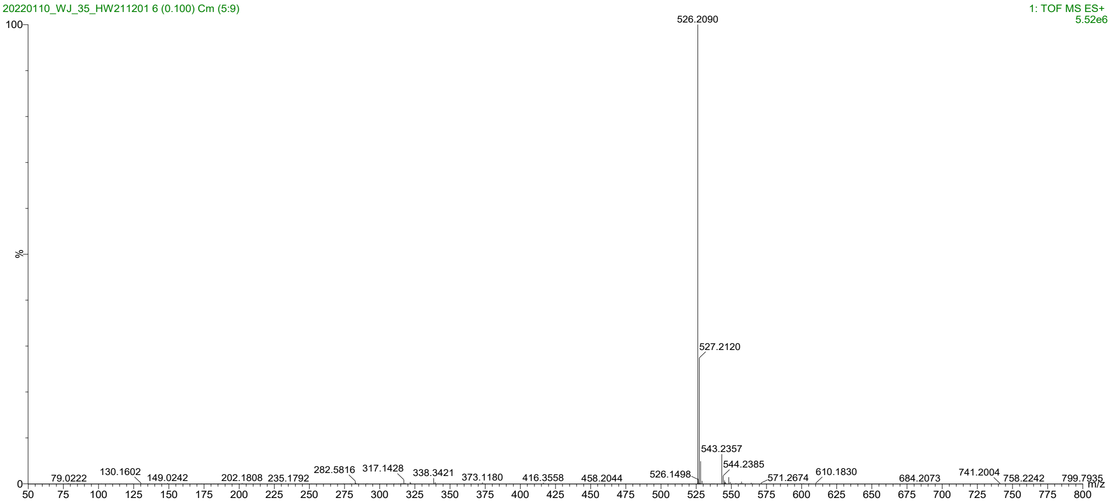

HPLC and HRMS of **H122**

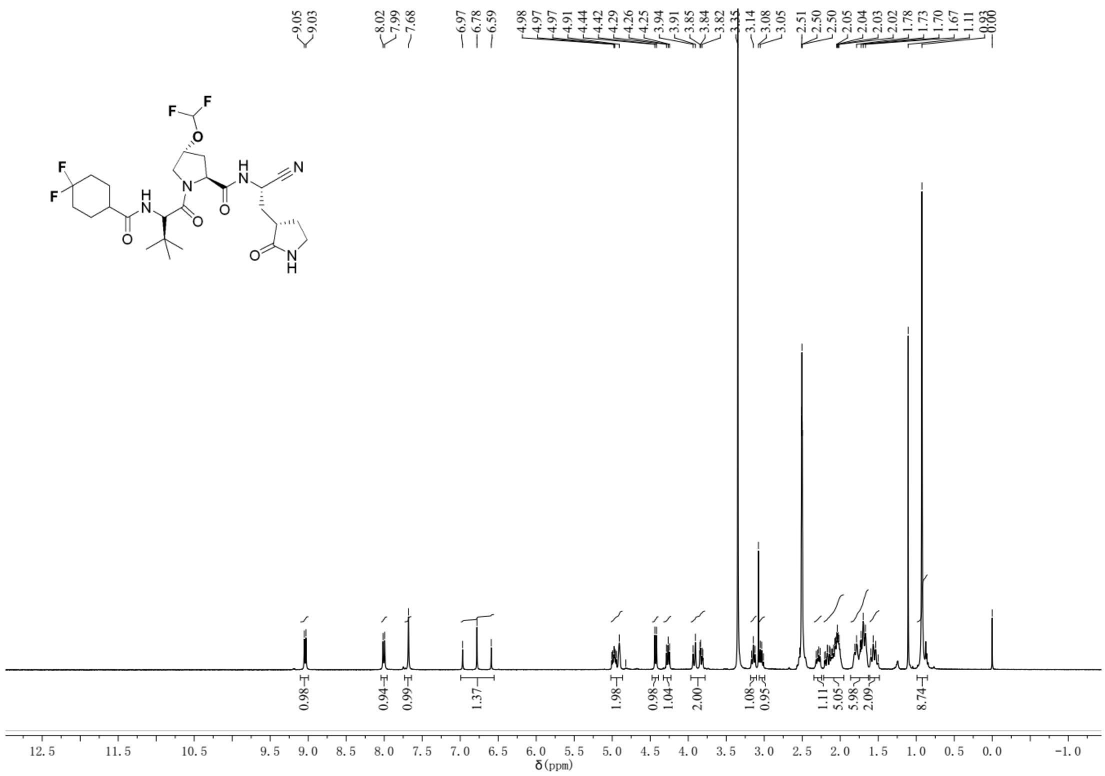

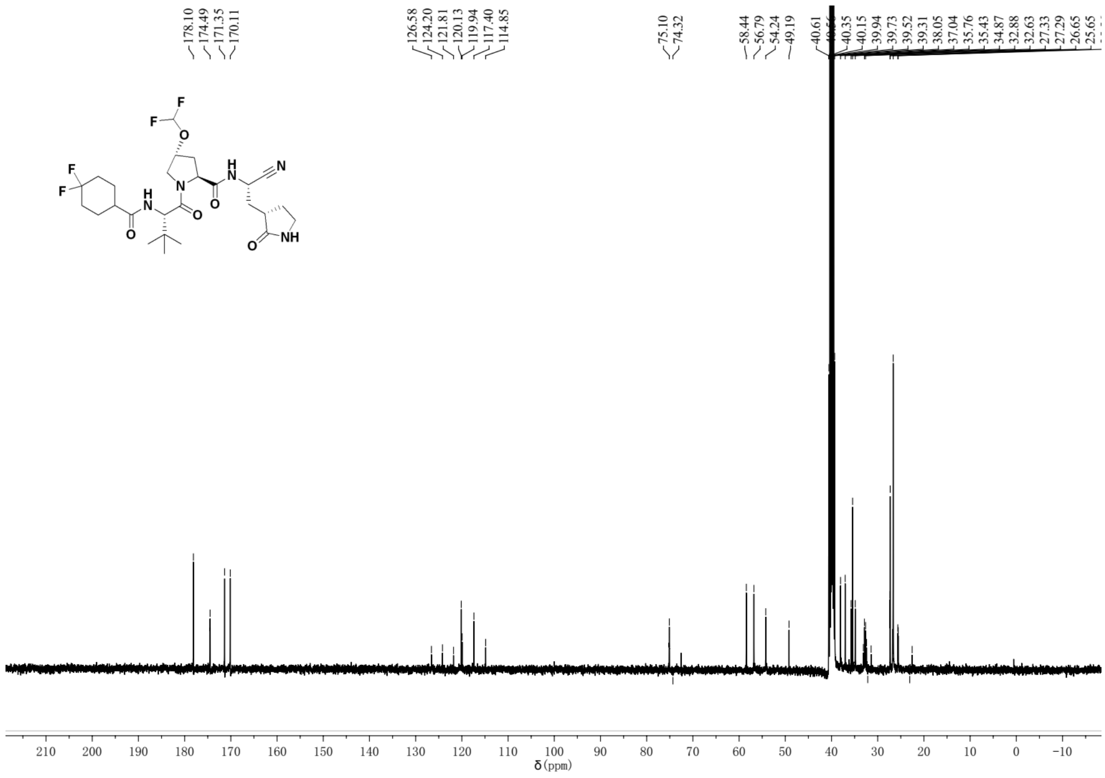

^{1}H NMR and ^{13}C NMR of **H123**

SHIMADZU LabSolutions **Analysis Report**

<Sample Information>

Sample Name	:
Sample ID	:
Data Filename	: HW211213-0-1-157A1.lcd
Method Filename	: proteasome intermediate.lcm
Batch Filename	:
Vial #	: 0
Injection Volume	: 5 uL
Date Acquired	: 12/19/2021 4:40:00 PM
Date Processed	: 1/4/2022 1:41:10 PM
Sample Type	: Unknown
Acquired by	: System Administrator
Processed by	: System Administrator

<Chromatogram>

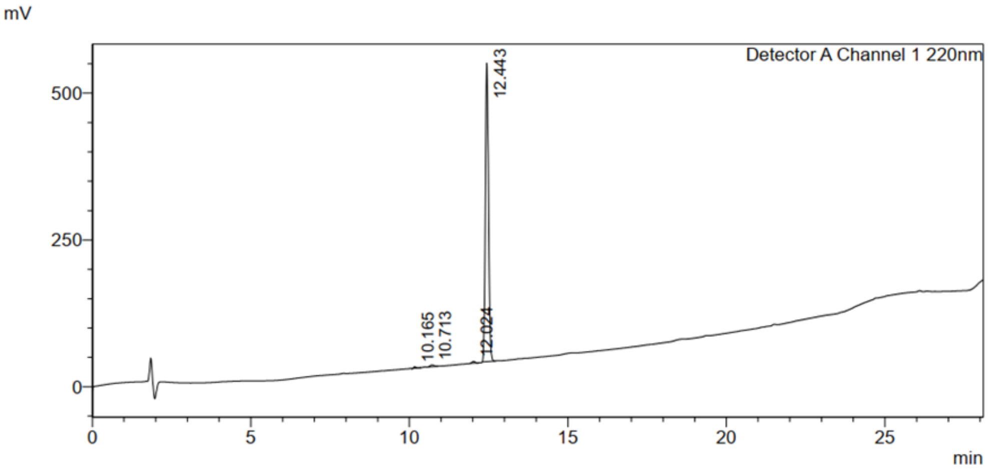

Peak#	Ret. Time	Area	Height	Area%	Height%	Conc.
1	10.165	15900	2860	0.445	0.553	0.445
2	10.713	24461	2775	0.684	0.536	0.684
3	12.024	19125	2914	0.535	0.563	0.535
4	12.443	3515109	508767	98.336	98.347	98.336
Total		3574595	517316	100.000	100.000	

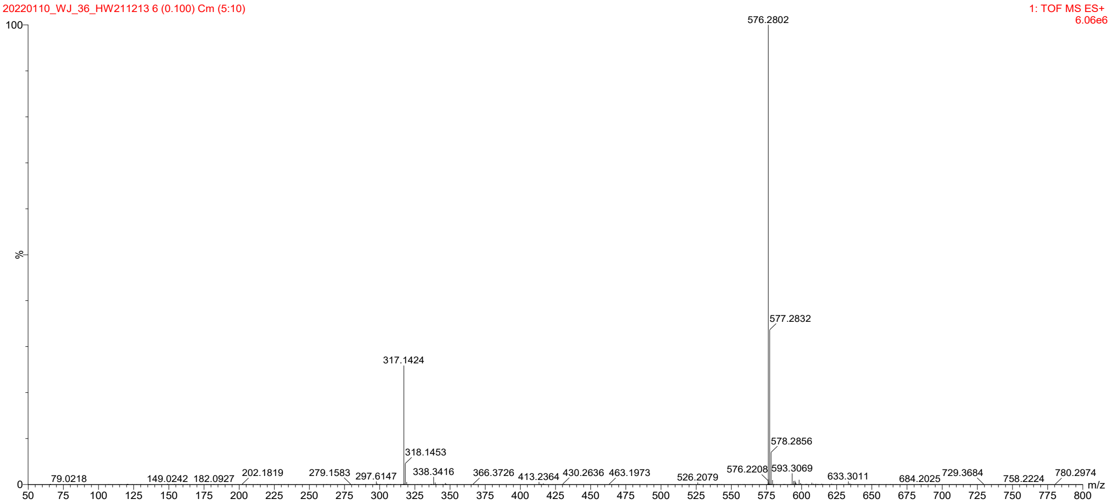

HPLC and HRMS of **H123**

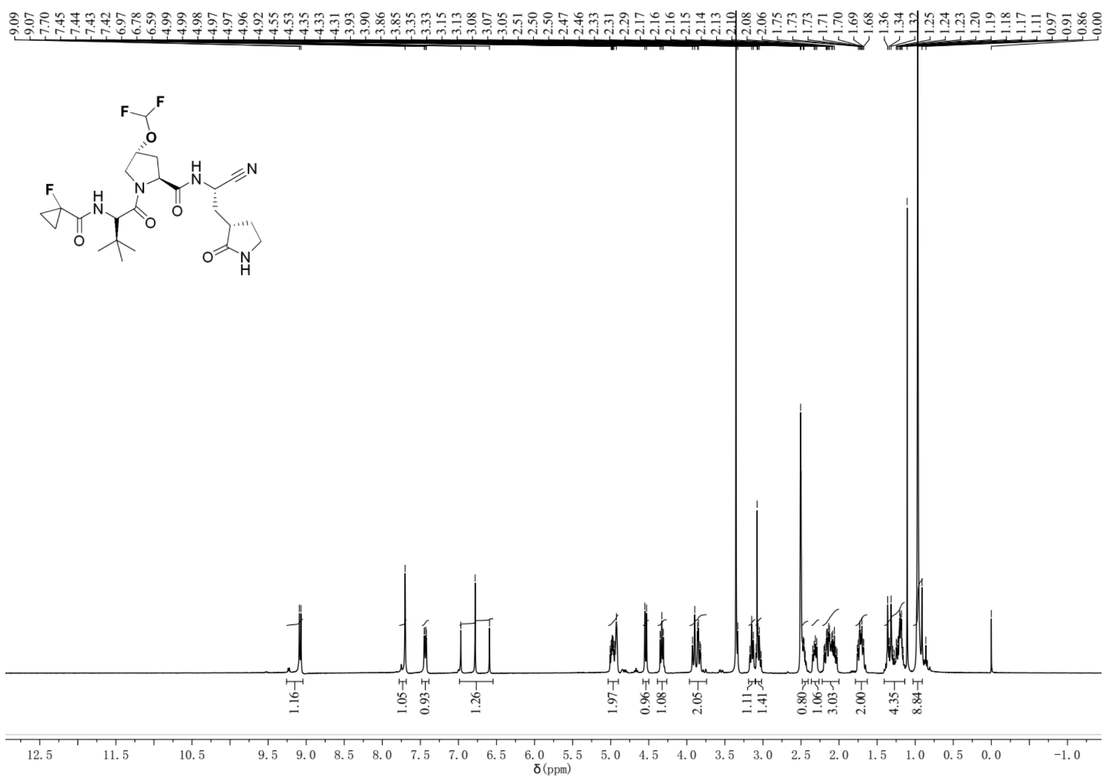

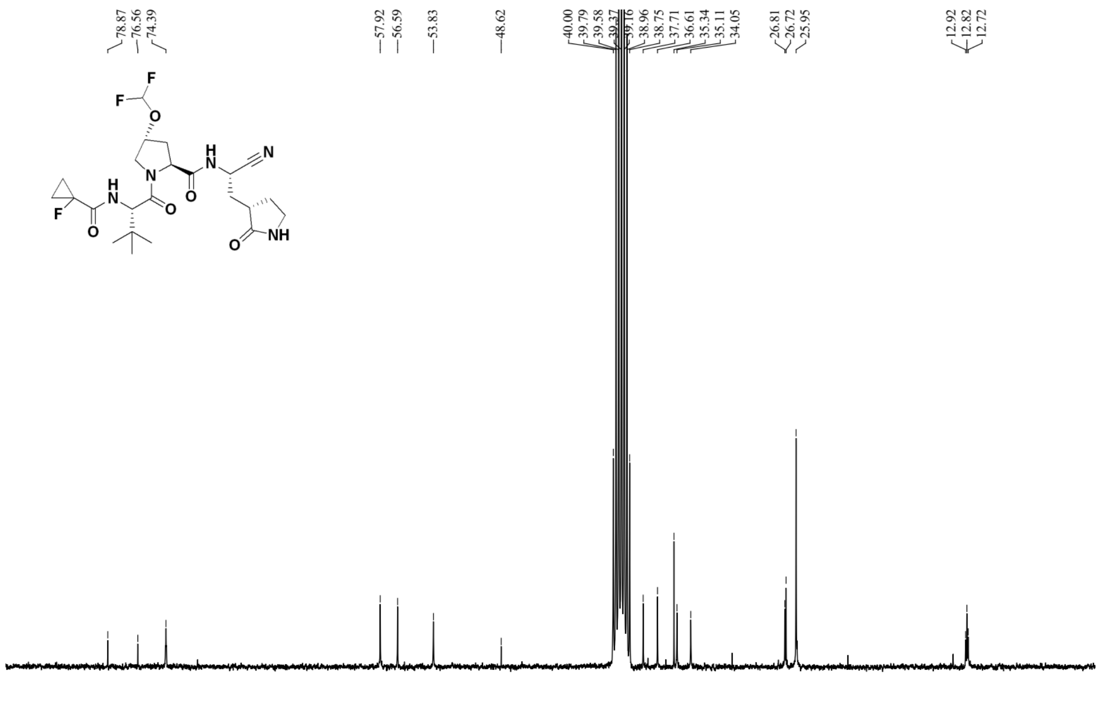

^{1}H NMR and ^{13}C NMR of **H124**

SHIMADZU LabSolutions

Analysis Report

<Sample Information>

Sample Name	:		
Sample ID	:		
Data Filename	: HW211215-0-1-161A1.lcd		
Method Filename	: proteasome intermediate.lcm		
Batch Filename	:		
Vial #	: 0	Sample Type	: Unknown
Injection Volume	: 5 uL		
Date Acquired	: 12/19/2021 4:09:45 PM	Acquired by	: System Administrator
Date Processed	: 1/4/2022 1:41:38 PM	Processed by	: System Administrator

<Chromatogram>

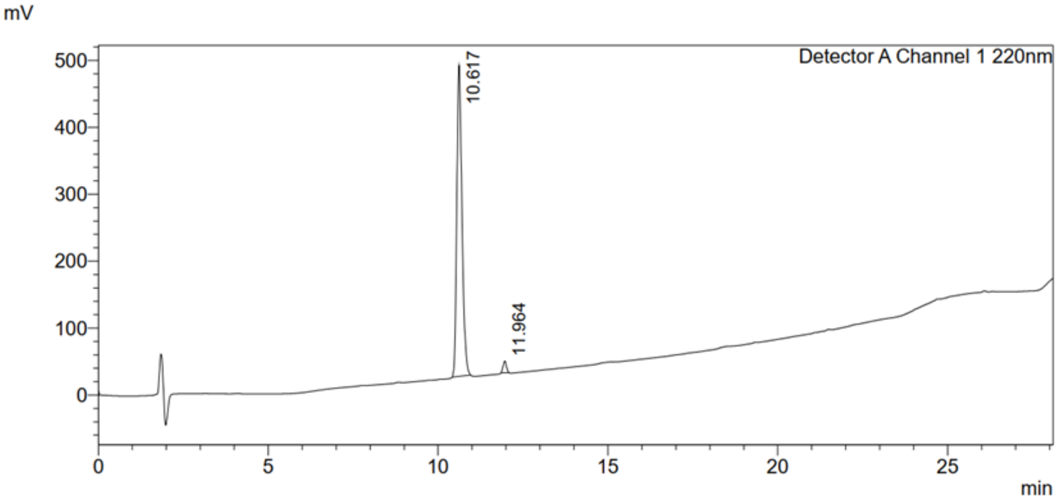

Peak#	Ret. Time	Area	Height	Area%	Height%	Conc.
1	10.617	4925751	464569	97.649	96.399	97.649
2	11.964	118614	17353	2.351	3.601	2.351
Total		5044366	481922	100.000	100.000	

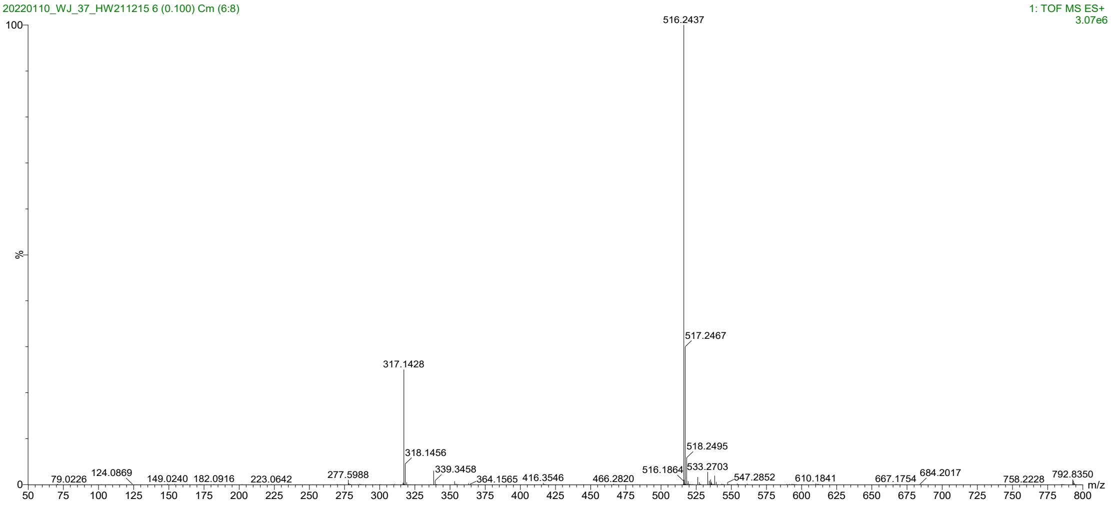

HPLC and HRMS of **H124**

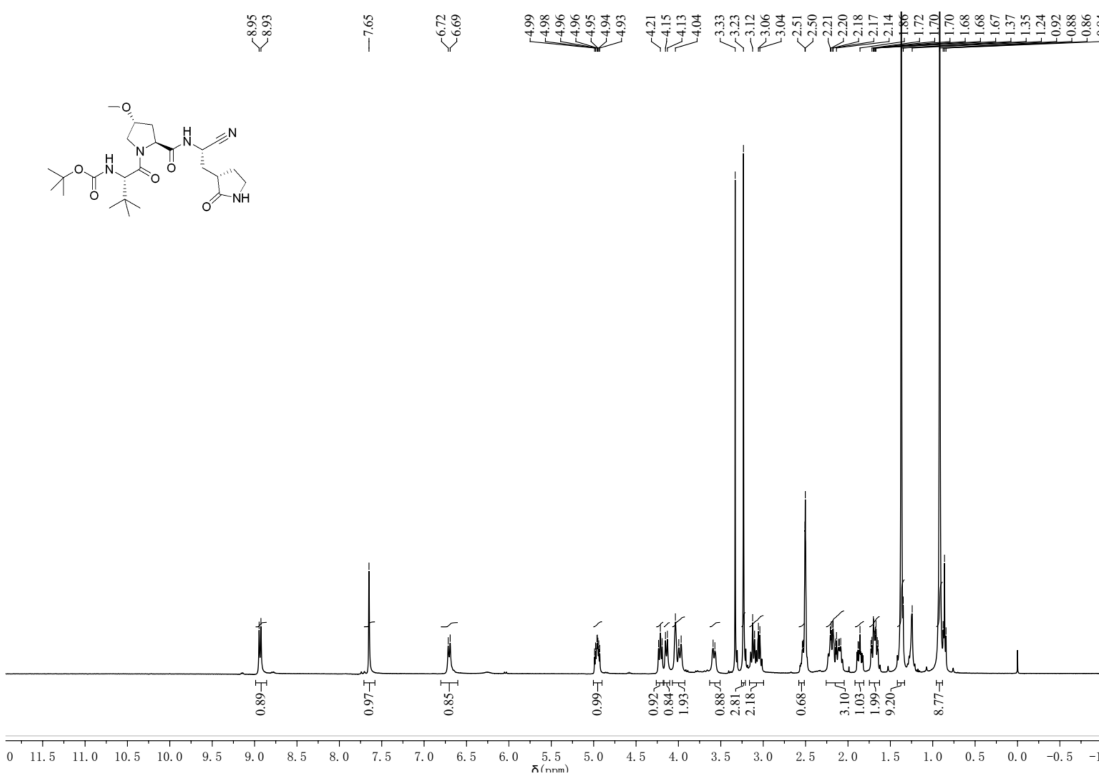

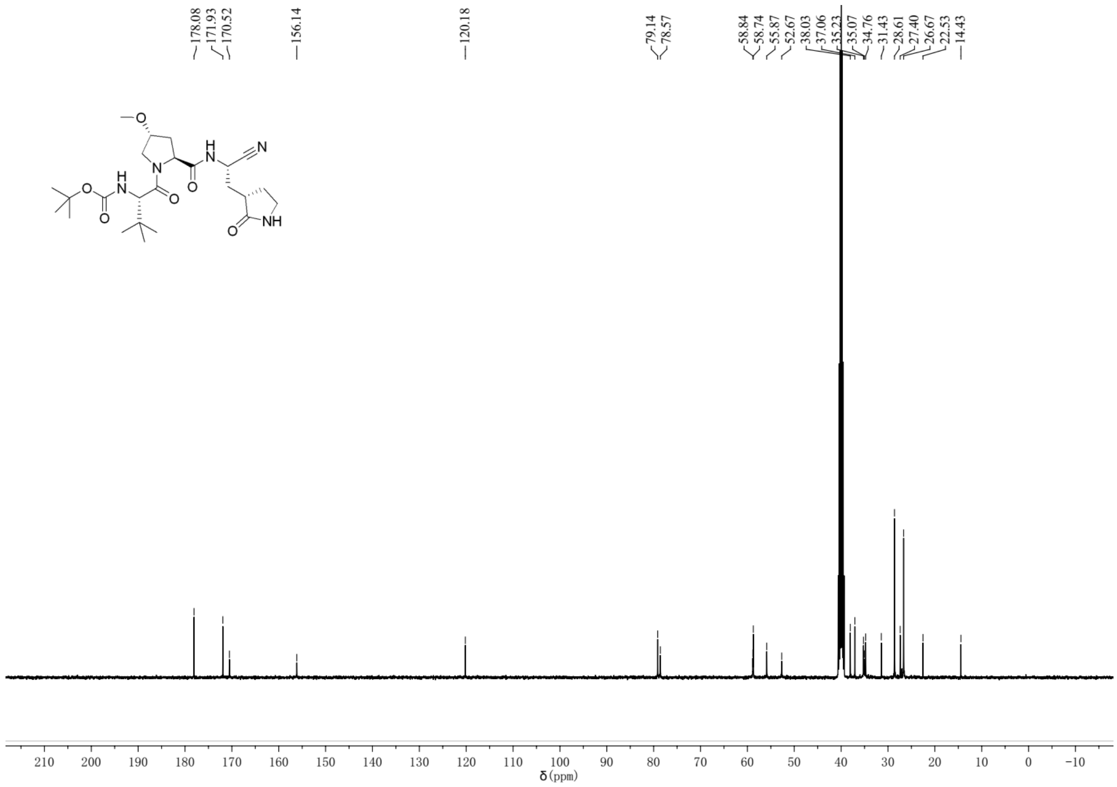

^{1}H NMR and ^{13}C NMR of **H138**

SHIMADZU LabSolutions

Analysis Report

<Sample Information>

Sample Name	:		
Sample ID	:		
Data Filename	: HW210916-0-1-97A2.lcd		
Method Filename	: proteasome intermediate.lcm		
Batch Filename	:		
Vial #	: 0	Sample Type	: Unknown
Injection Volume	: 5 uL		
Date Acquired	: 10/20/2021 5:17:32 PM	Acquired by	: System Administrator
Date Processed	: 10/20/2021 5:45:40 PM	Processed by	: System Administrator

<Chromatogram>

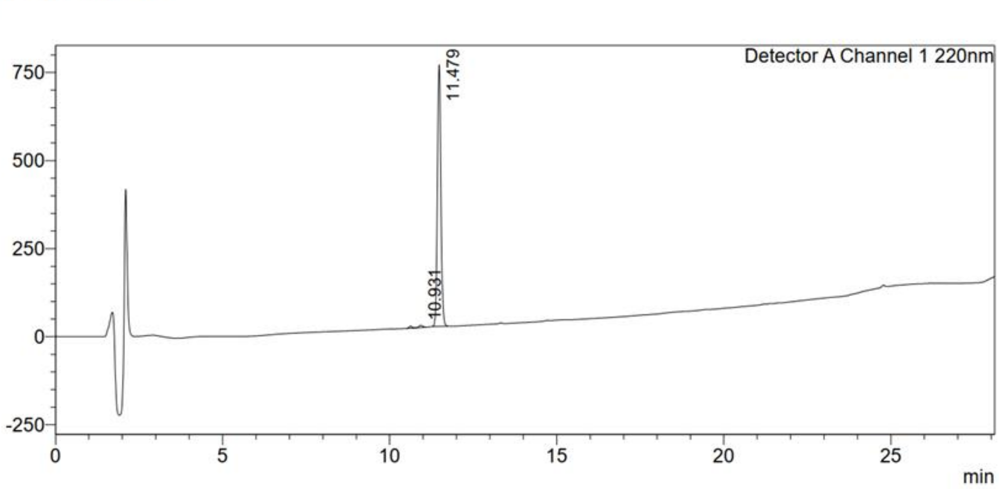

Peak#	Ret. Time	Area	Height	Area%	Height%	Conc.
1	10.931	81272	6177	1.493	0.826	1.493
2	11.479	5363631	741677	98.507	99.174	98.507
Total		5444903	747855	100.000	100.000	

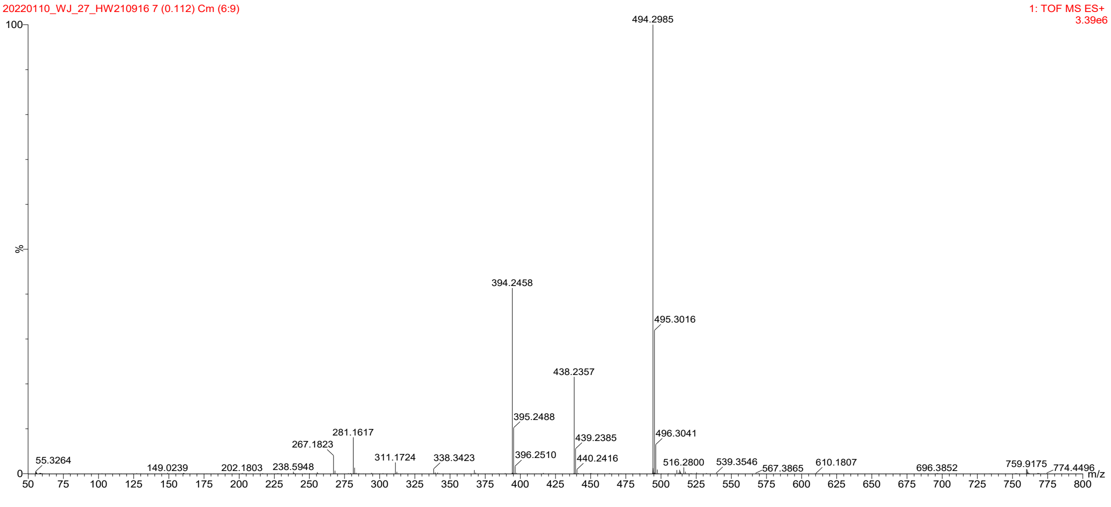

HPLC and HRMS of **H138**

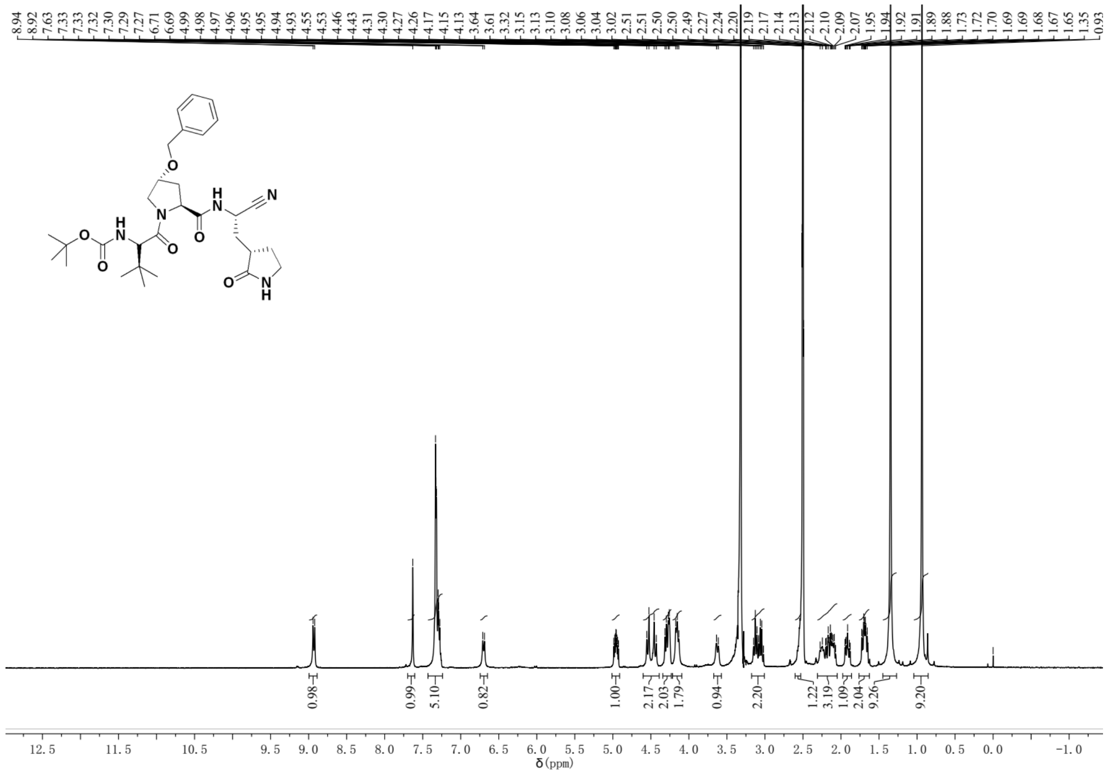

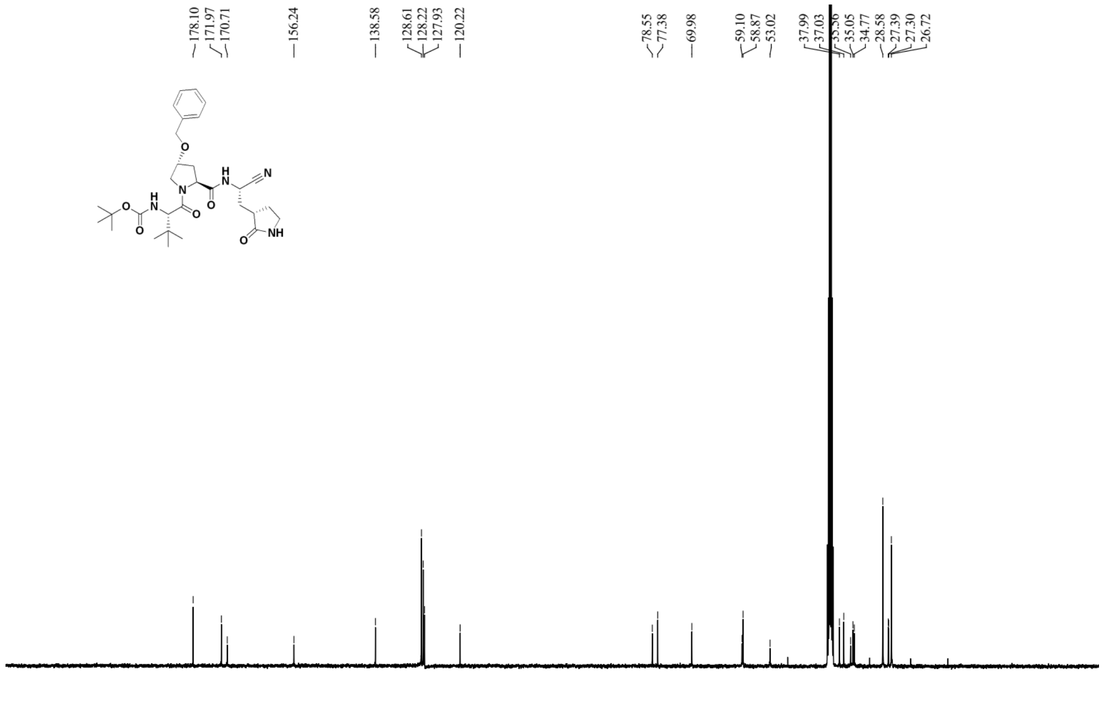

^{1}H NMR and ^{13}C NMR of **H139**

<Sample Information>

Sample Name	:		
Sample ID	:		
Data Filename	: HW211114-0-1-123A1.lcd		
Method Filename	: proteasome intermediate.lcm		
Batch Filename	:		
Vial #	: 0	Sample Type	: Unknown
Injection Volume	: 5 uL		
Date Acquired	: 11/20/2021 7:04:44 PM	Acquired by	: System Administrator
Date Processed	: 11/20/2021 7:32:50 PM	Processed by	: System Administrator

<Chromatogram>

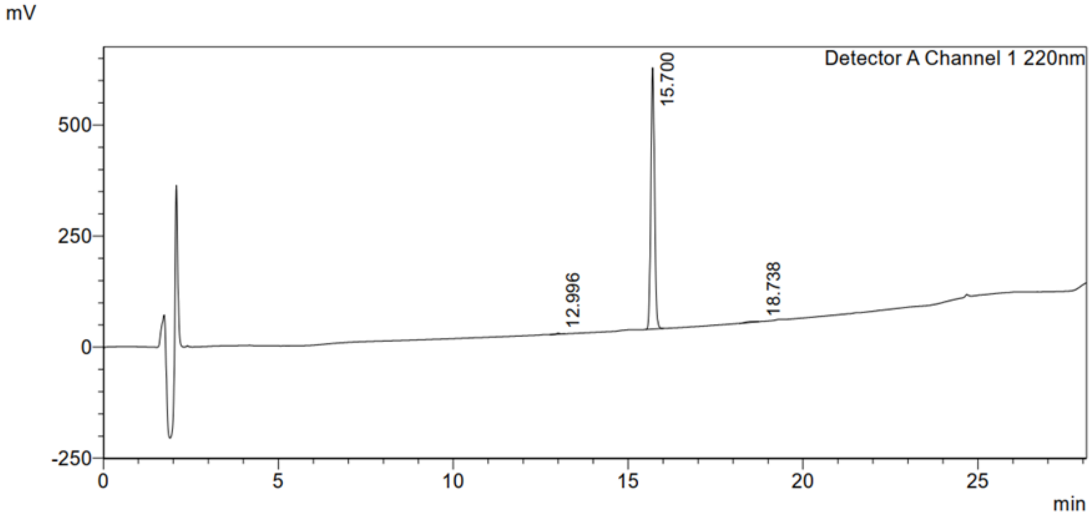

Peak#	Ret. Time	Area	Height	Area%	Height%	Conc.
1	12.996	13745	2182	0.314	0.369	0.314
2	15.700	4331571	588353	99.023	99.630	99.023
3	18.738	28988	5	0.663	0.001	0.663
Total		4374304	590540	100.000	100.000	

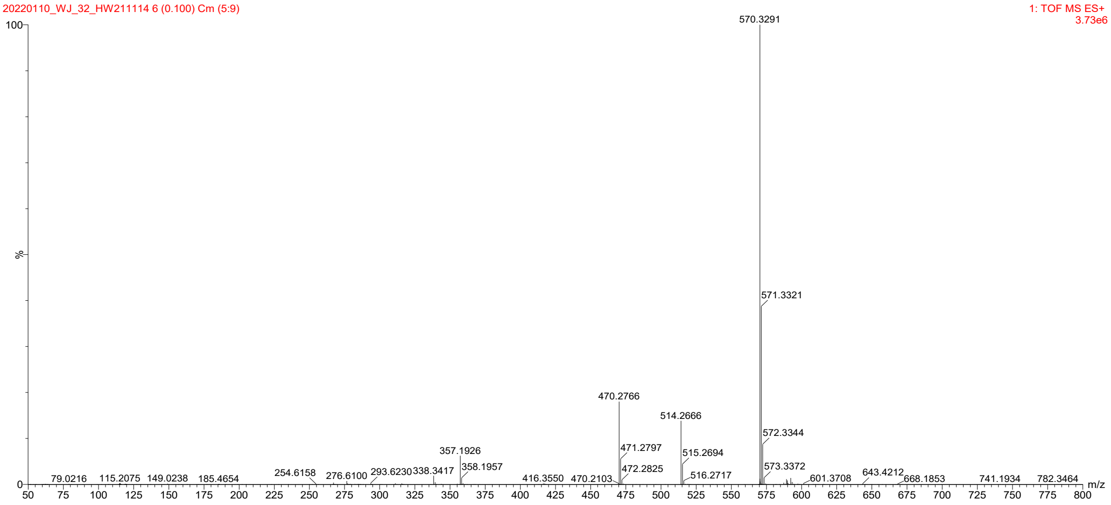

HPLC and HRMS of **H139**

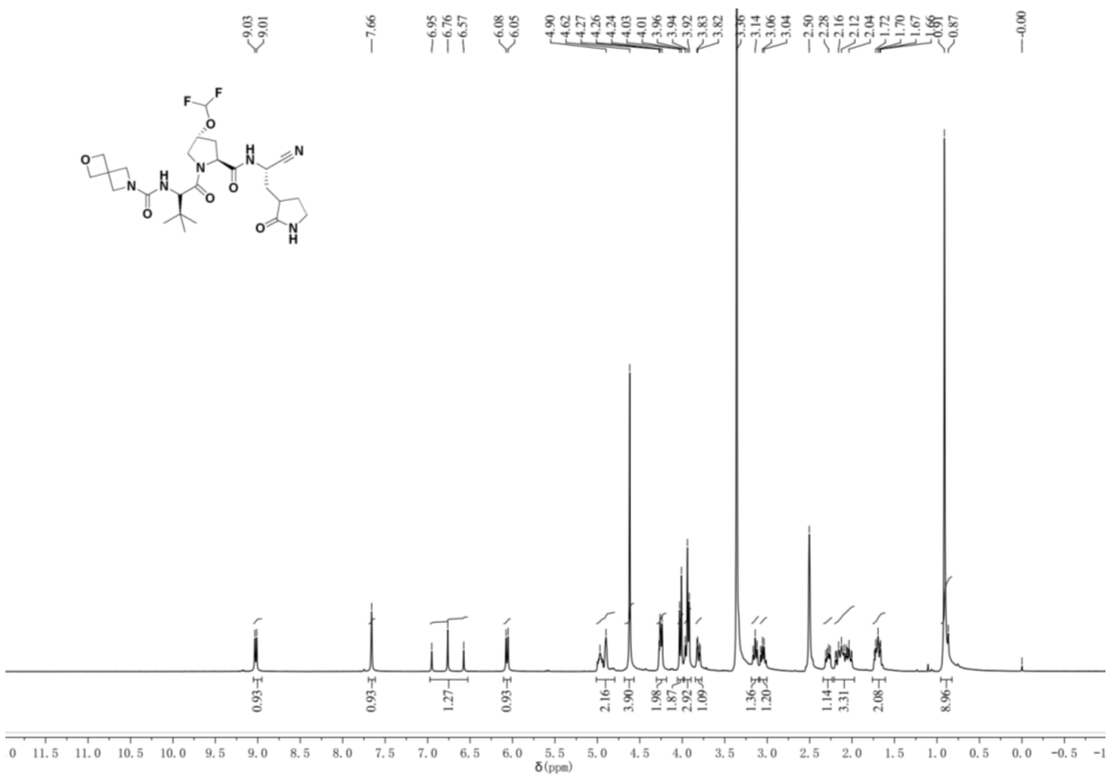

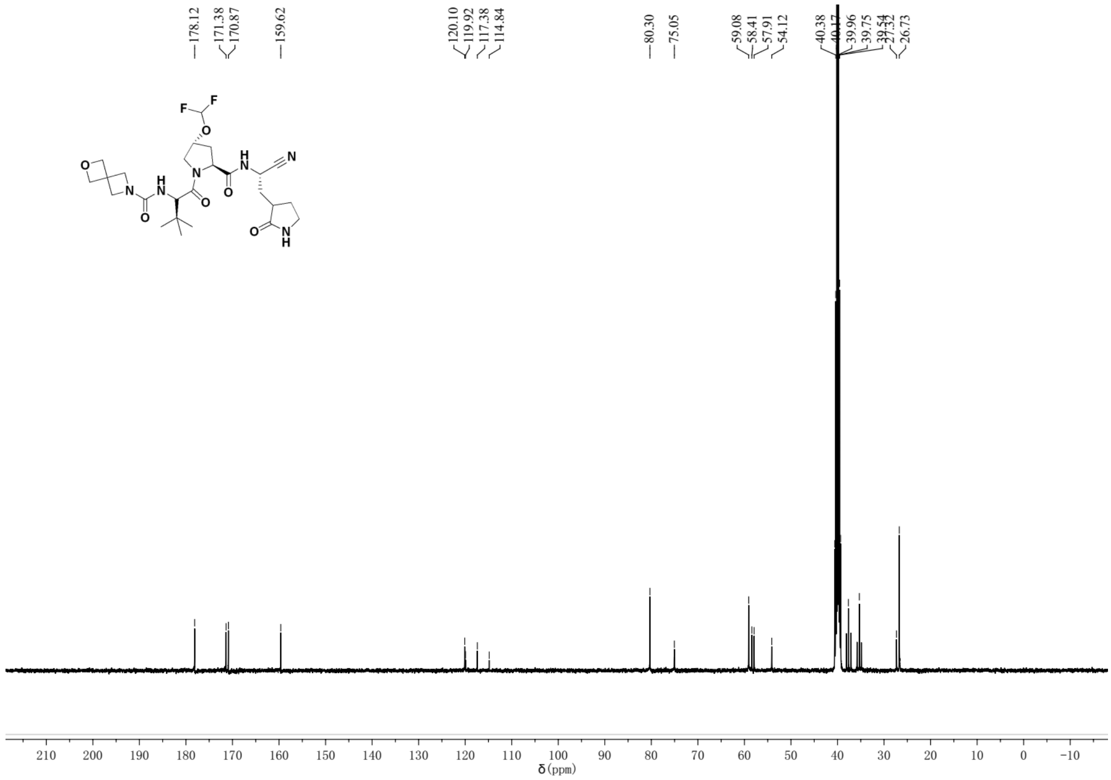

^{1}H NMR and ^{13}C NMR of **H129**

SHIMADZU LabSolutions

Analysis Report

<Sample Information>

Sample Name	:		
Sample ID	:		
Data Filename	: HW220405-0-1-001A2.lcd		
Method Filename	: proteasome intermediate10-85%.lcm		
Batch Filename	:		
Vial #	: 1	Sample Type	: Unknown
Injection Volume	: 8 uL		
Date Acquired	: 5/3/2022 9:12:50 PM	Acquired by	: System Administrator
Date Processed	: 5/3/2022 9:51:05 PM	Processed by	: System Administrator

<Chromatogram>

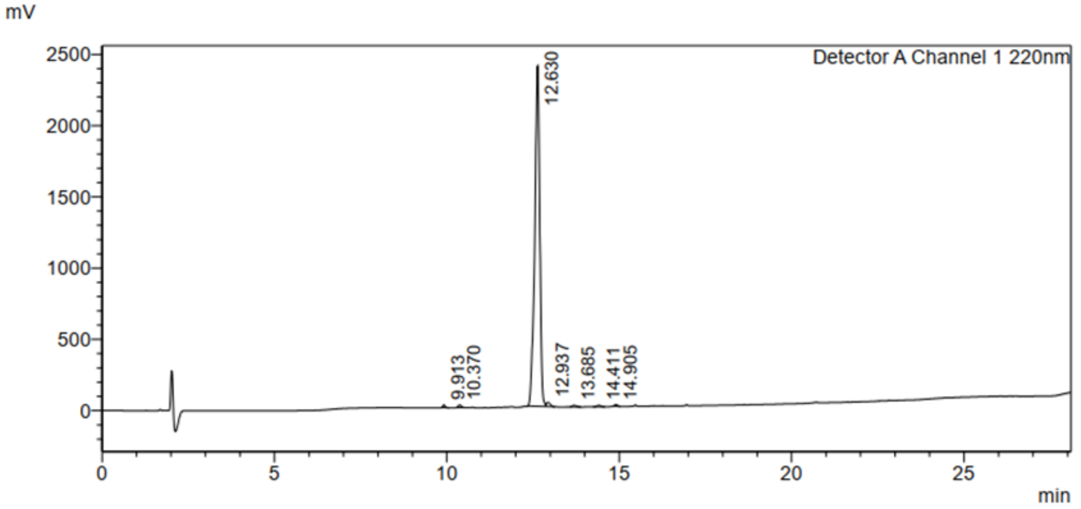

Peak#	Ret. Time	Area	Height	Area%	Height%	Conc.
1	9.913	93985	18754	0.379	0.755	0.379
2	10.370	126618	18686	0.511	0.752	0.511
3	12.630	24122911	2386576	97.272	96.068	97.272
4	12.937	211859	27712	0.854	1.115	0.854
5	13.685	90072	10501	0.363	0.423	0.363
6	14.411	81397	10037	0.328	0.404	0.328
7	14.905	72525	11982	0.292	0.482	0.292
Total		24799366	2484247	100.000	100.000	

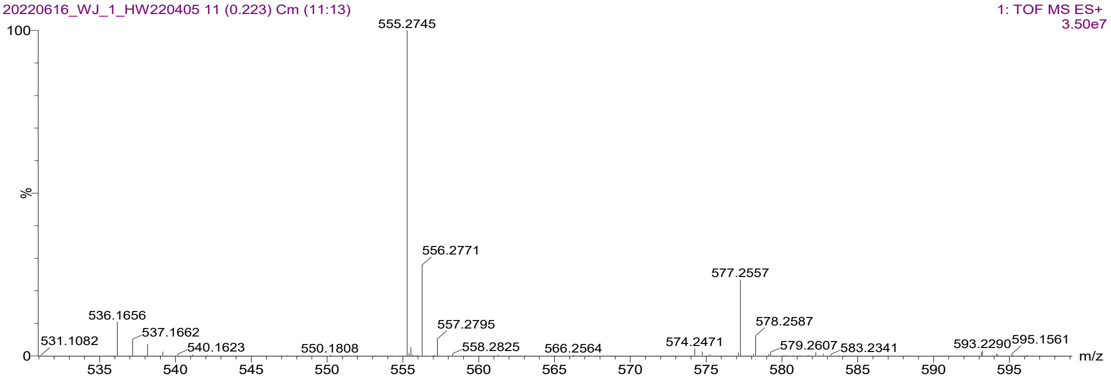

HPLC and HRMS of **H129**

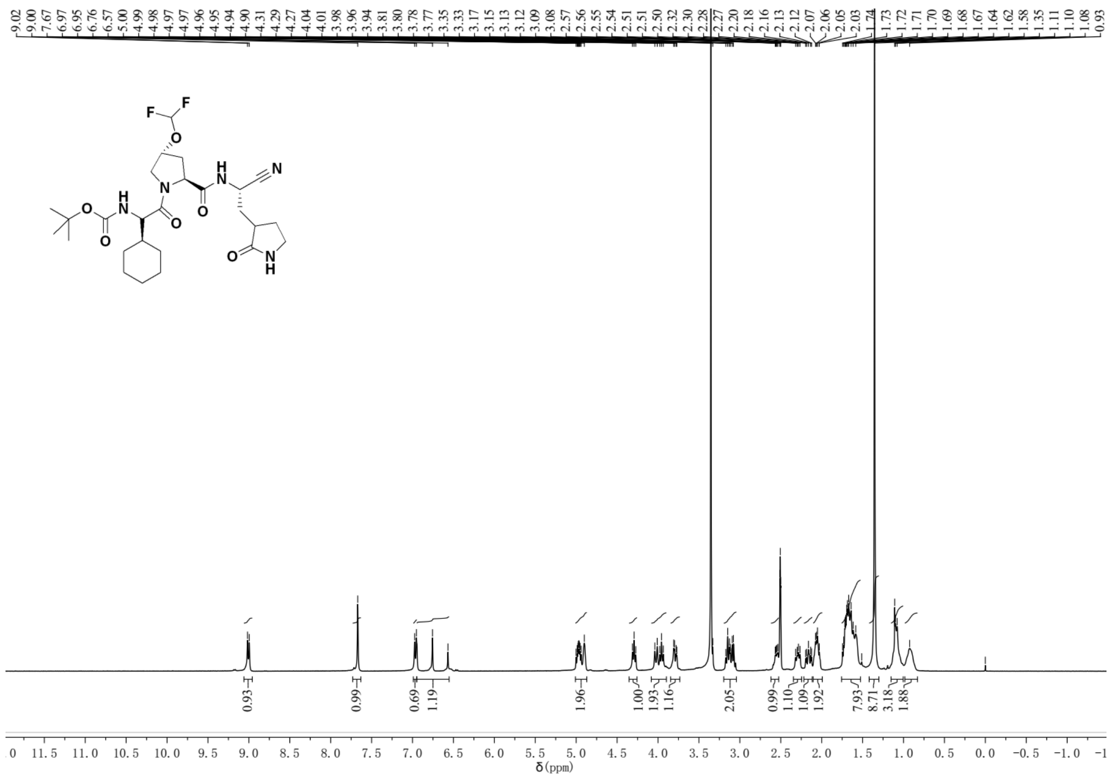
9.02 9.00 7.67 6.97 6.95 6.76 6.57 5.00 4.99 4.98 4.97 4.97 4.96 4.95 4.94 4.90 4.31 4.29 4.27 4.04 4.01 3.98 3.96 3.94 3.81 3.80 3.78 3.77 3.35 3.33 3.17 3.15 3.13 3.12 3.09 3.08 2.57 2.56 2.55 2.54 2.51 2.51 2.50 2.32 2.30 2.28 2.27 2.20 2.18 2.16 2.13 2.12 2.07 2.06 2.05 2.03 1.74 1.73 1.72 1.71 1.70 1.69 1.68 1.67 1.64 1.62 1.58 1.35 1.11 1.10 1.08 0.93
0.93 0.99 0.69 1.19 1.96 1.00 1.93 1.16 2.05 0.99 1.10 1.09 1.92 7.93 8.71 3.18 1.88
11.5 11.0 10.5 10.0 9.5 9.0 8.5 8.0 7.5 7.0 6.5 6.0 5.5 5.0 4.5 4.0 3.5 3.0 2.5 2.0 1.5 1.0 0.5 0.0 −0.5 −1.0
δ(ppm)

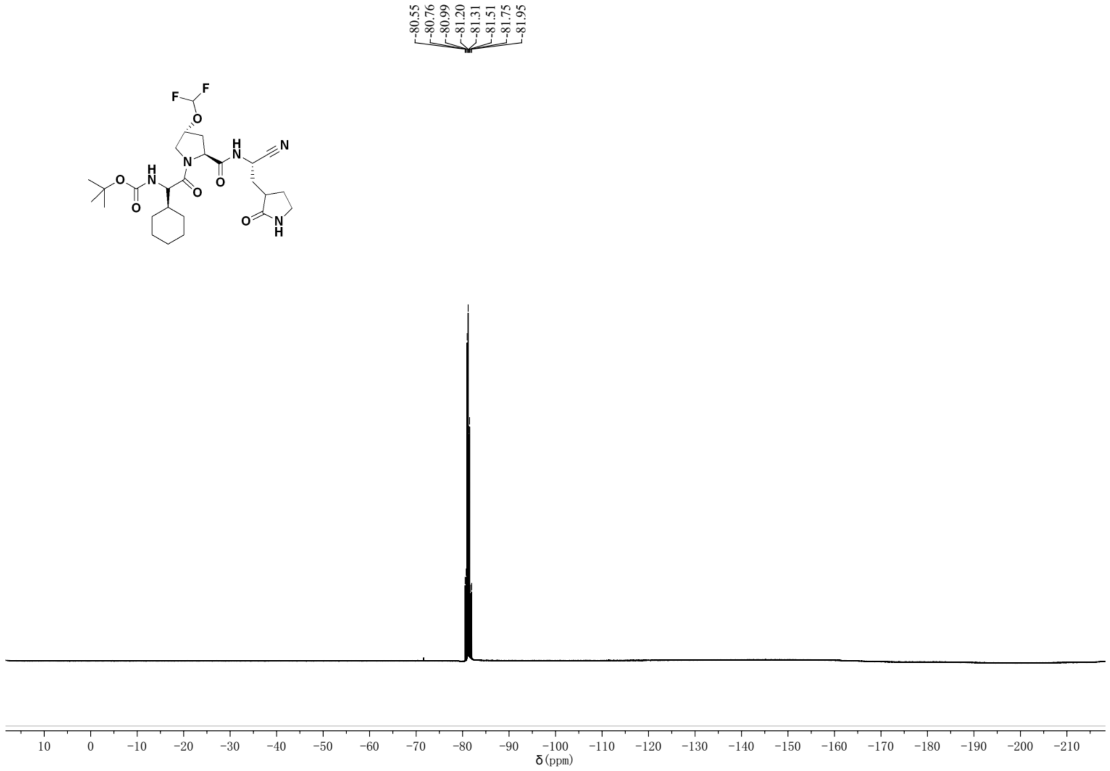
-80.55 -80.76 -80.99 -81.20 -81.31 -81.51 -81.75 -81.95
10 0 −10 −20 −30 −40 −50 −60 −70 −80 −90 −100 −110 −120 −130 −140 −150 −160 −170 −180 −190 −200 −210
δ(ppm)

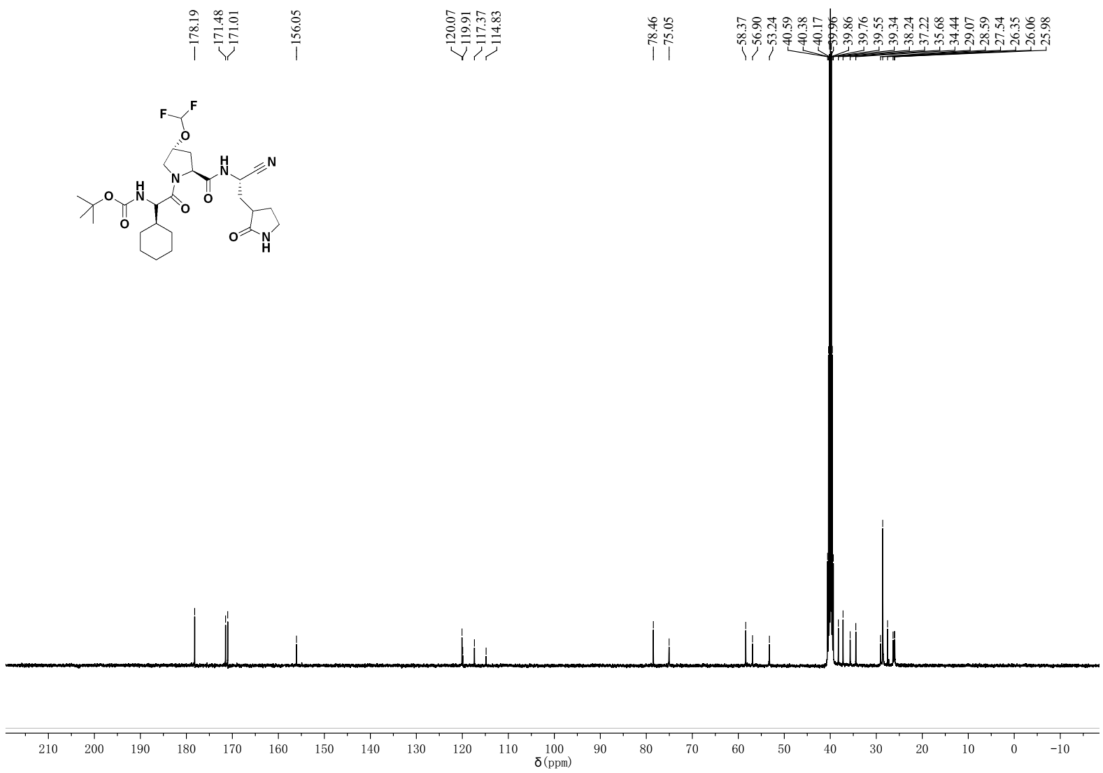

^{1}H NMR, ^{19}F NMR and ^{13}C NMR of **H130**

SHIMADZU LabSolutions

Analysis Report

<Sample Information>

Sample Name :
Sample ID :
Data Filename : HW220412-0-1-191A1.lcd
Method Filename : proteasome intermediate.lcm
Batch Filename :
Vial # : 1
Sample Type : Unknown
Injection Volume : 5 uL
Date Acquired : 4/22/2022 1:07:08 PM
Acquired by : System Administrator
Date Processed : 4/22/2022 7:48:59 PM
Processed by : System Administrator

<Chromatogram>

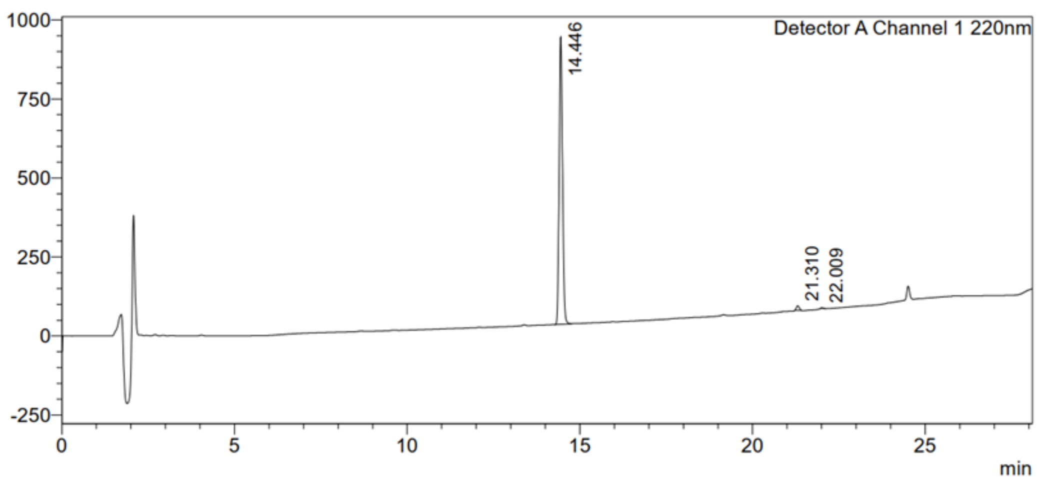

Peak#	Ret. Time	Area	Height	Area%	Height%	Conc.
1	14.446	6141702	908385	98.457	98.179	98.457
2	21.310	85132	14497	1.365	1.567	1.365
3	22.009	11113	2355	0.178	0.255	0.178
Total		6237947	925237	100.000	100.000	

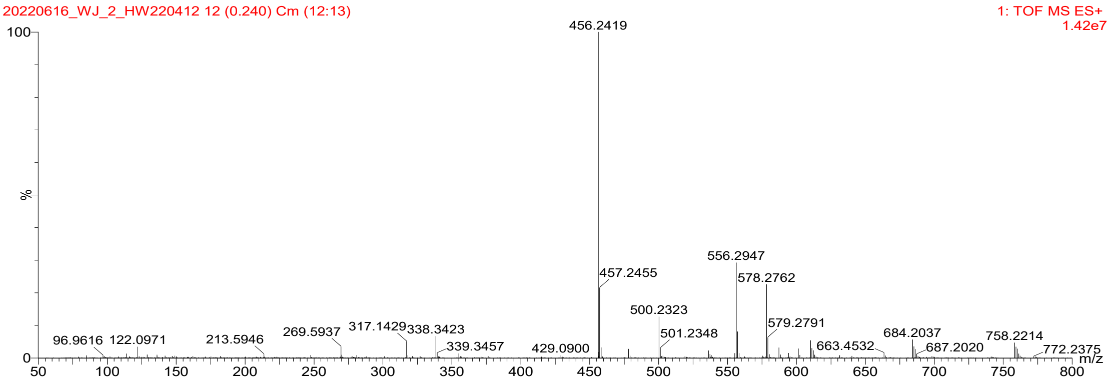

HPLC and HRMS of **H130**

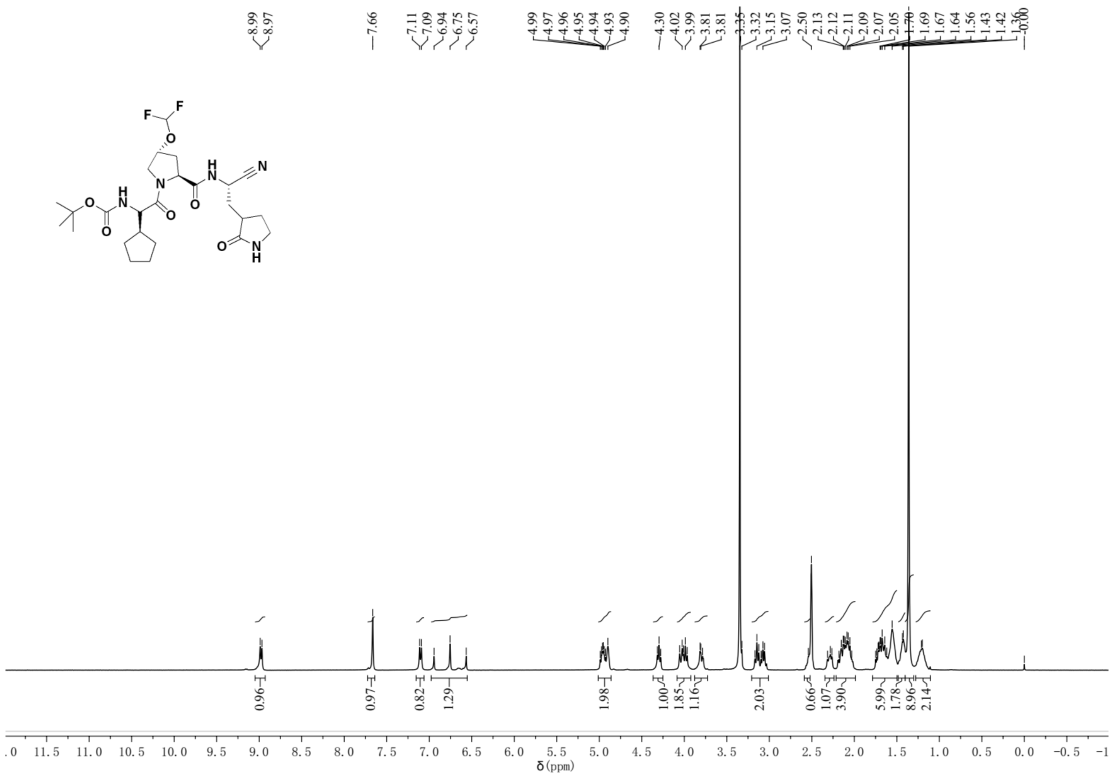

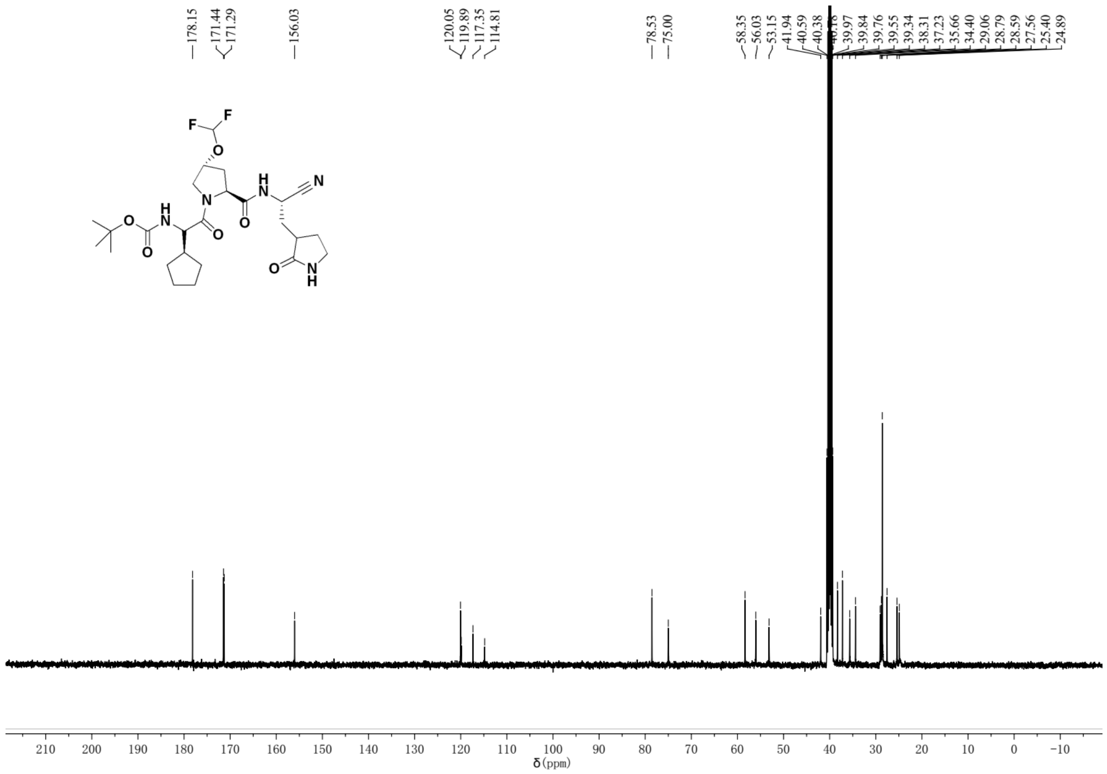

^{1}H NMR and ^{13}C NMR of **H131**

Analysis Report

SHIMADZU LabSolutions

<Sample Information>

Sample Name :
Sample ID :
Data Filename : HW220418-0-1-194A1.lcd
Method Filename : proteasome intermediate.lcm
Batch Filename :
Vial # : 1
Sample Type : Unknown
Injection Volume : 5 uL
Date Acquired : 4/22/2022 3:25:35 PM
Acquired by : System Administrator
Date Processed : 4/22/2022 3:53:42 PM
Processed by : System Administrator

<Chromatogram>

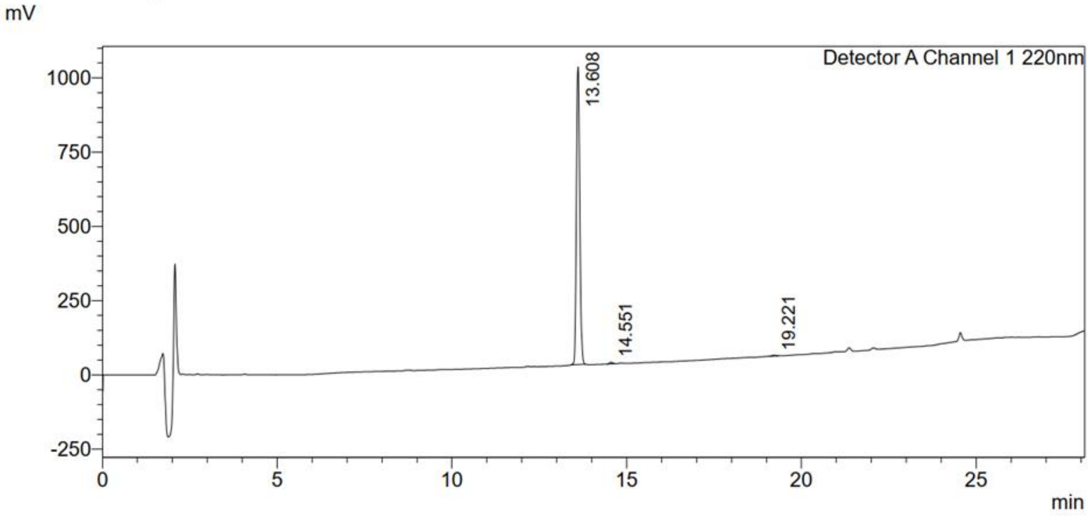

Peak#	Ret. Time	Area	Height	Area%	Height%	Conc.
1	13.608	6648230	1001632	99.188	99.202	99.188
2	14.551	35926	5653	0.536	0.560	0.536
3	19.221	18466	2408	0.276	0.239	0.276
Total		6702622	1009693	100.000	100.000	

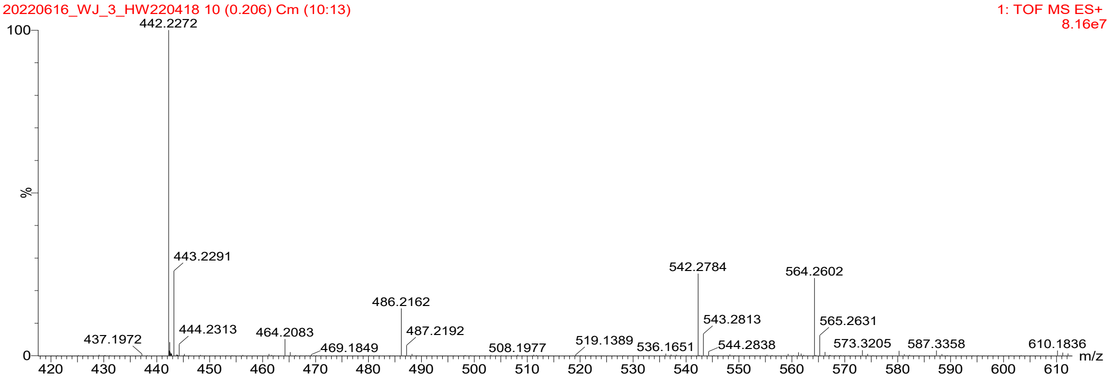

HPLC and HRMS of **H131**

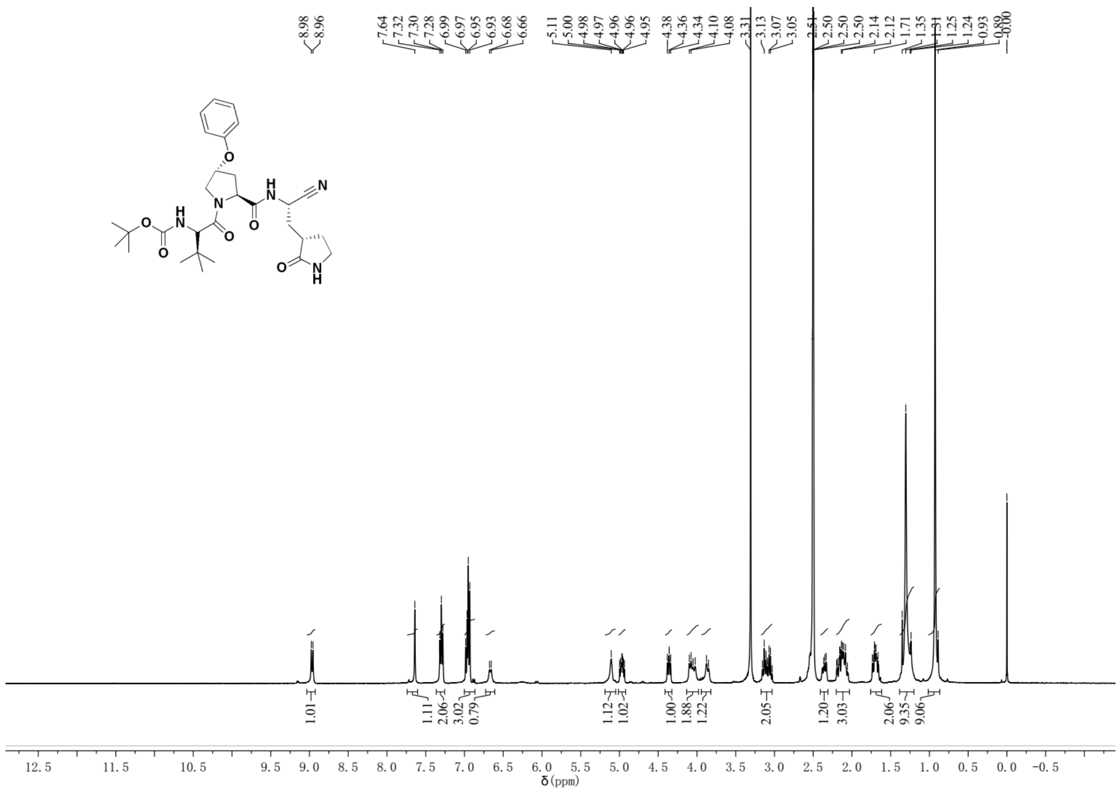

^{1}H NMR of **H140**

SHIMADZU LabSolutions

Analysis Report

<Sample Information>

Sample Name	:		
Sample ID	:		
Data Filename	: HW211124-0-1-139A1.lcd		
Method Filename	: proteasome intermediate.lcm		
Batch Filename	:		
Vial #	: 0	Sample Type	: Unknown
Injection Volume	: 5 uL		
Date Acquired	: 12/2/2021 4:11:47 PM	Acquired by	: System Administrator
Date Processed	: 12/2/2021 4:39:54 PM	Processed by	: System Administrator

<Chromatogram>

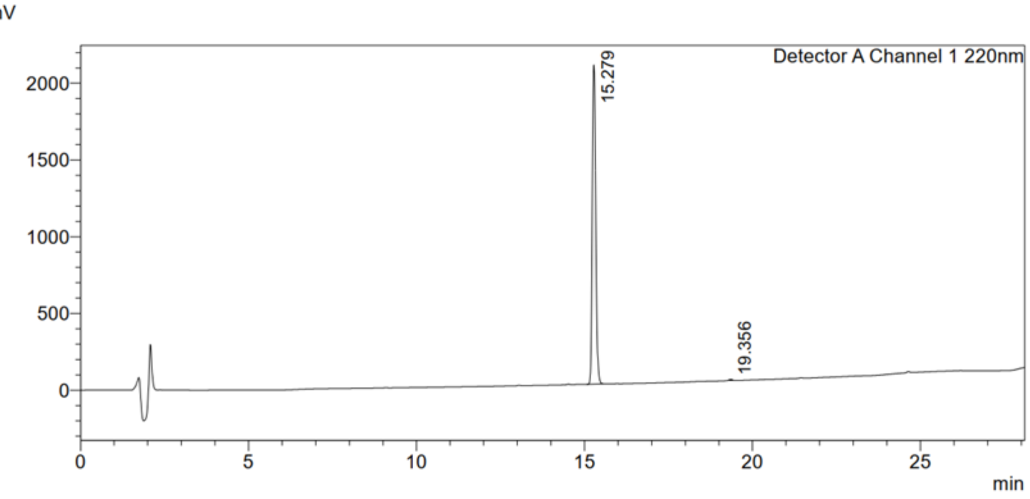

Peak#	Ret. Time	Area	Height	Conc.
1	15.279	14898838	2076194	99.674
2	19.356	48699	7405	0.326
Total		14947537	2083599	

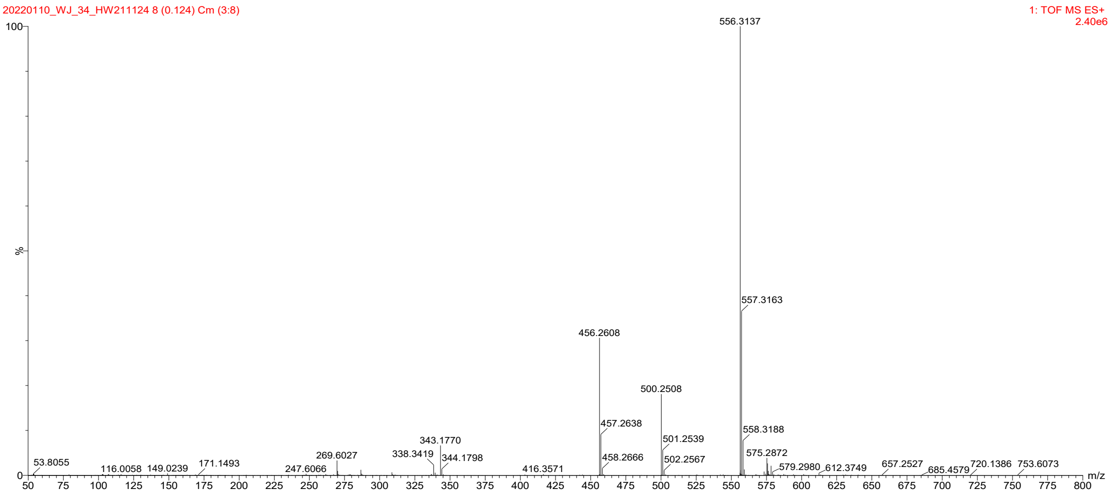

HPLC and HRMS of **H140**

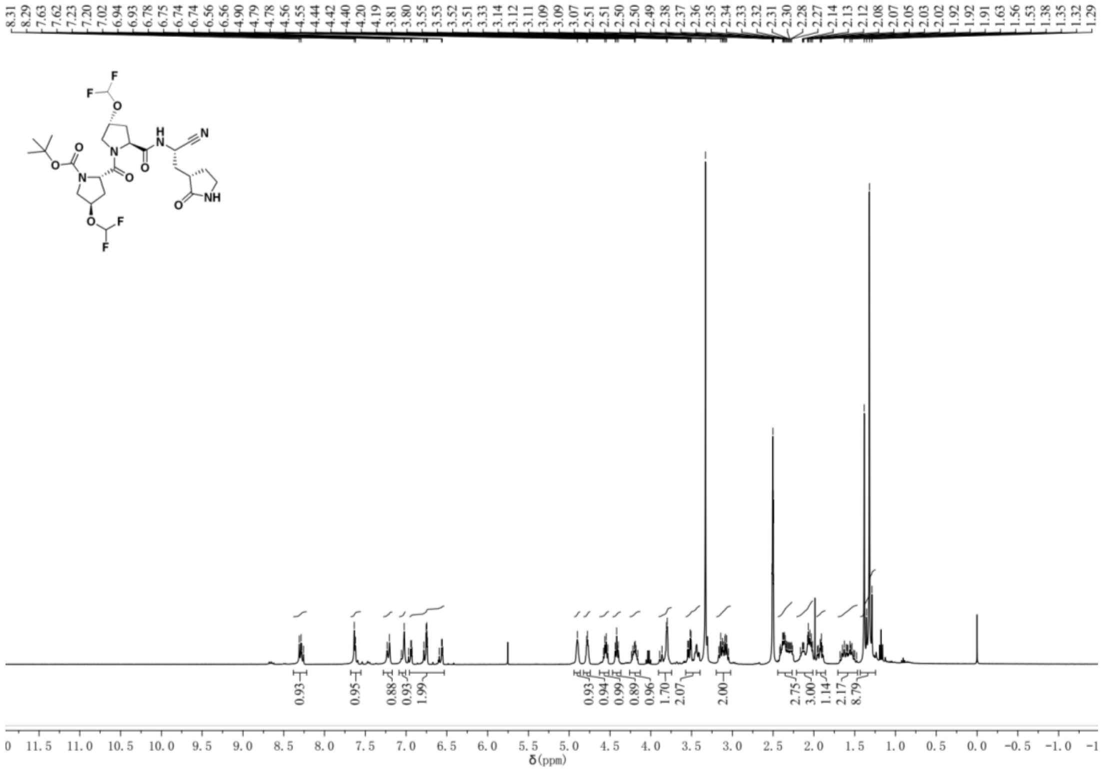
F
F
O
O
N
O
O
N
O
N
H
N
O
NH
O
F
F
8.31 8.29 7.63 7.62 7.23 7.20 7.02 6.94 6.93 6.78 6.75 6.74 6.56 6.56 4.90 4.79 4.78 4.56 4.55 4.44 4.42 4.40 4.20 4.19 3.81 3.80 3.55 3.53 3.52 3.51 3.33 3.14 3.12 3.11 3.09 3.09 3.07 2.51 2.51 2.50 2.50 2.49 2.38 2.37 2.36 2.35 2.34 2.33 2.32 2.31 2.30 2.28 2.27 2.14 2.13 2.12 2.08 2.07 2.05 2.03 2.02 1.92 1.92 1.91 1.63 1.56 1.53 1.38 1.35 1.32 1.29
0.93 0.95 0.88 0.93 1.99 0.93 0.94 0.99 0.89 0.96 1.70 2.07 2.00 2.75 3.00 1.14 2.17 8.79
δ (ppm)

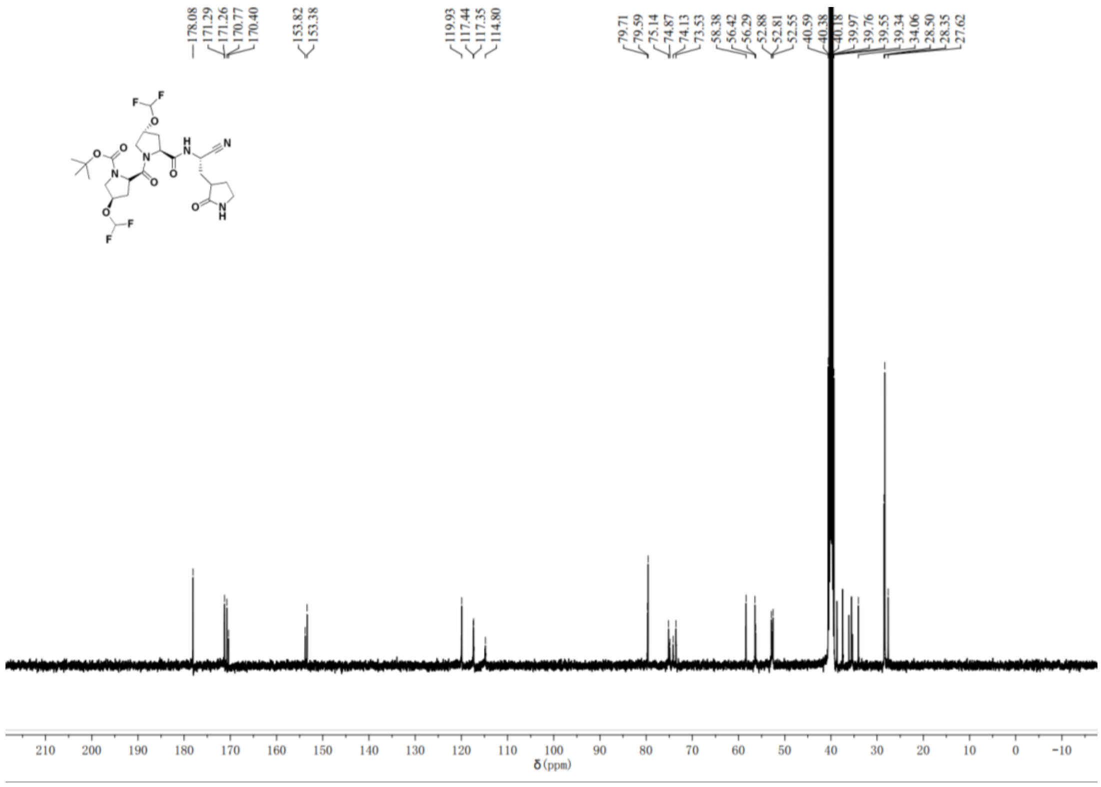
178.08 171.29 171.26 170.77 170.40 153.82 153.38 119.93 117.44 117.35 114.80 79.71 79.59 75.14 74.87 74.13 73.53 58.38 56.42 56.29 52.88 52.81 52.55 40.59 40.38 40.18 39.97 39.76 39.55 39.34 34.06 28.50 28.35 27.62
δ (ppm)

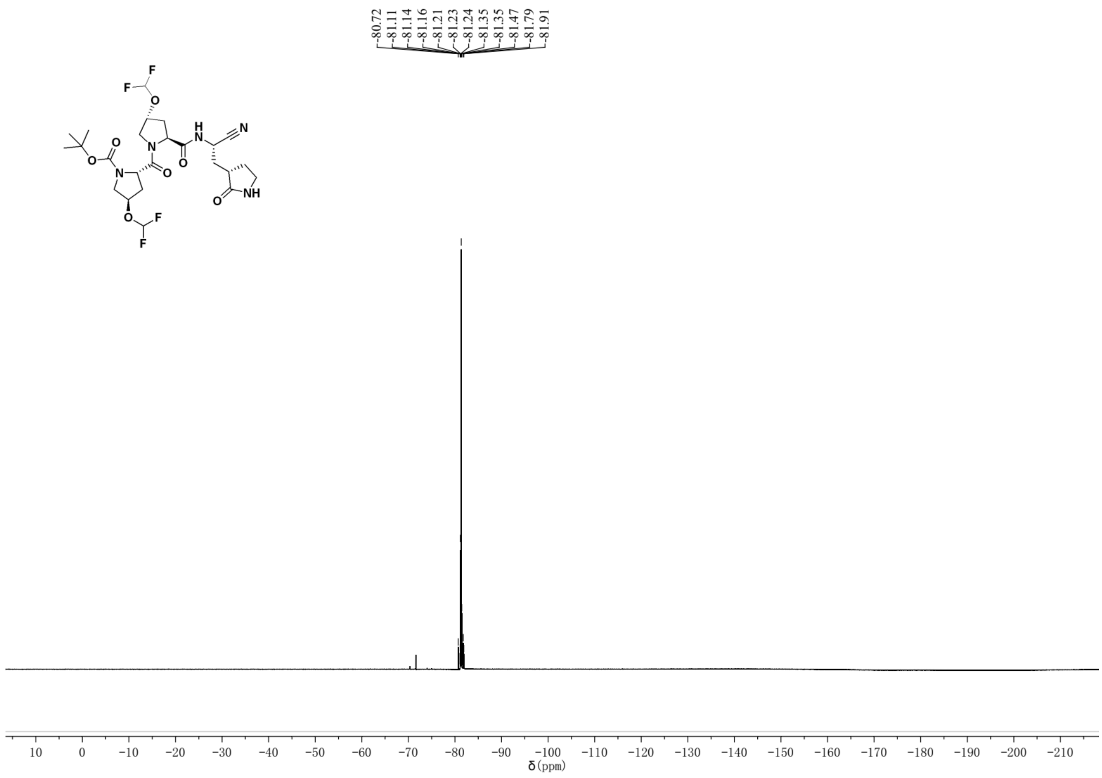

^{1}H NMR, ^{19}F NMR and ^{13}C NMR of **H141**

Analysis Report

SHIMADZU LabSolutions

<Sample Information>

Sample Name	:		
Sample ID	:		
Data Filename	: HW220423-0-1-197A1.lcd		
Method Filename	: proteasome intermediate.lcm		
Batch Filename	:		
Vial #	: 1	Sample Type	: Unknown
Injection Volume	: 8 uL		
Date Acquired	: 5/3/2022 8:03:13 PM	Acquired by	: System Administrator
Date Processed	: 5/3/2022 8:31:20 PM	Processed by	: System Administrator

<Chromatogram>

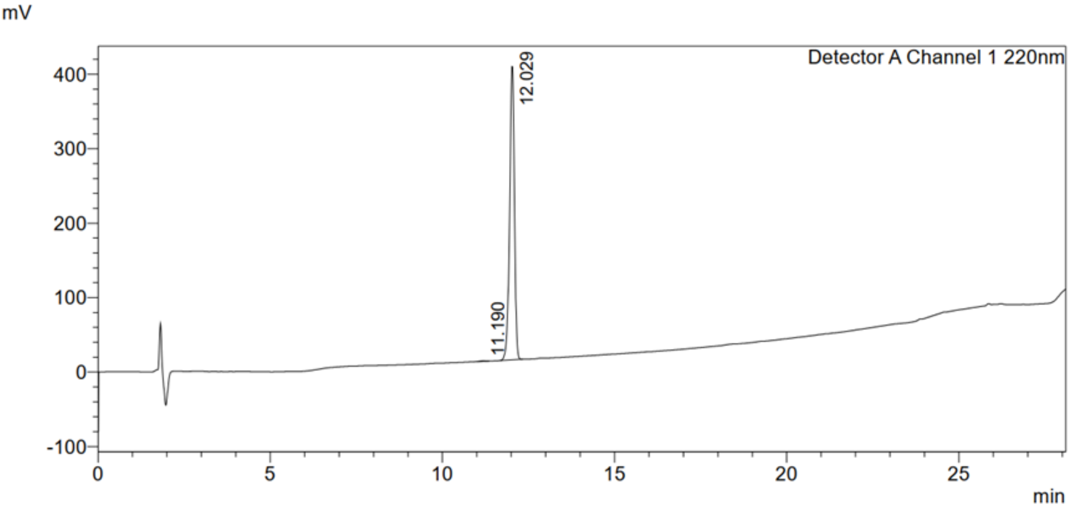

Peak#	Ret. Time	Area	Height	Area%	Height%	Conc.
1	11.190	7618	868	0.200	0.220	0.200
2	12.029	3803377	393818	99.800	99.780	99.800
Total		3810995	394686	100.000	100.000	

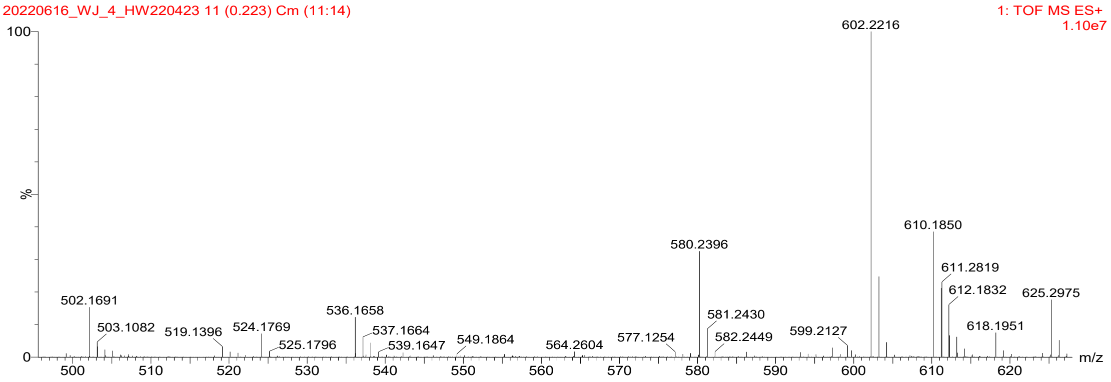

HPLC and HRMS of **H141**

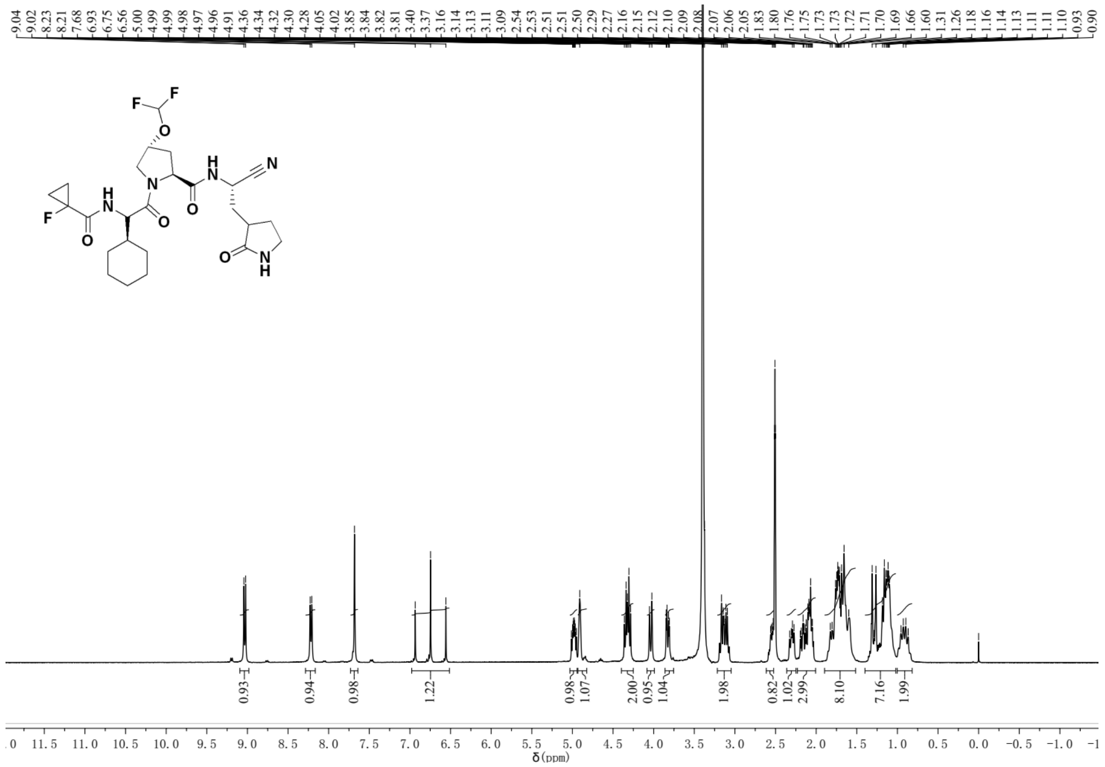
9.04 9.02 8.23 8.21 7.68 6.93 6.75 6.56 5.00 4.99 4.99 4.98 4.97 4.96 4.91 4.36 4.34 4.32 4.30 4.28 4.05 4.02 3.85 3.84 3.82 3.81 3.40 3.37 3.16 3.14 3.13 3.11 3.09 2.54 2.53 2.51 2.50 2.29 2.27 2.16 2.15 2.12 2.10 2.09 2.08 2.07 2.06 2.05 1.83 1.80 1.76 1.75 1.73 1.73 1.72 1.71 1.70 1.69 1.66 1.60 1.31 1.26 1.18 1.16 1.14 1.13 1.11 1.11 1.10 0.93 0.90
0.93 0.94 0.98 1.22 0.98 1.07 2.00 0.95 1.04 1.98 0.82 1.02 2.99 8.10 7.16 1.99
δ(ppm)

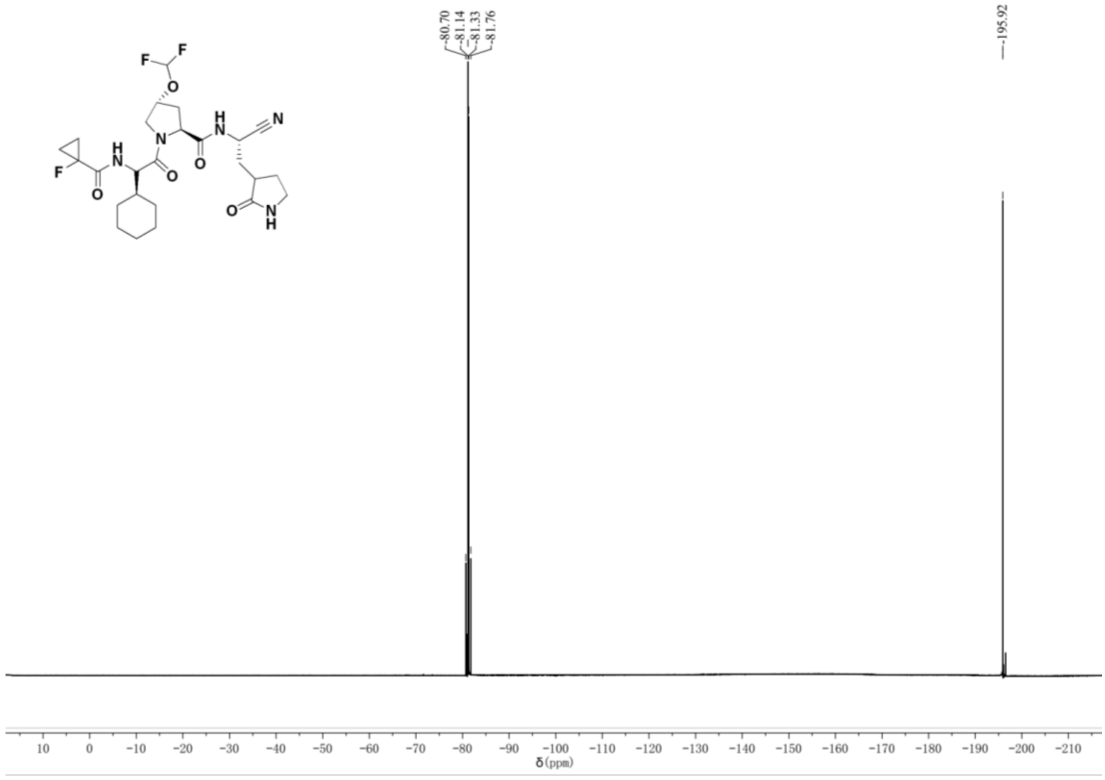
-80.70 -81.14 -81.33 -81.76
-195.92
δ(ppm)

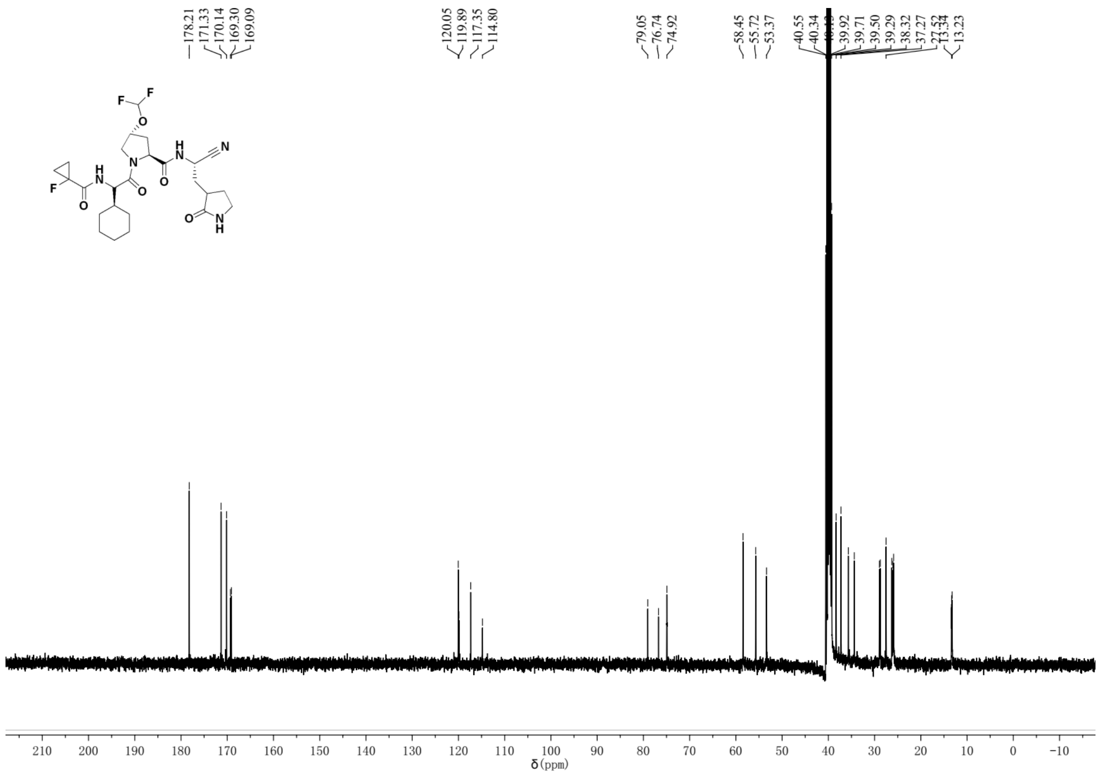

^{1}H NMR, ^{19}F NMR and ^{13}C NMR of **H136**

SHIMADZU LabSolutions

Analysis Report

<Sample Information>

Sample Name	: Dual_V3		
Sample ID	:		
Data Filename	: HW220604-0-1-35A3.lcd		
Method Filename	: proteasome intermediate.lcm		
Batch Filename	:		
Vial #	: 2	Sample Type	: Unknown
Injection Volume	: 5 uL		
Date Acquired	: 6/13/2022 4:08:40 PM	Acquired by	: System Administrator
Date Processed	: 6/13/2022 4:36:48 PM	Processed by	: System Administrator

<Chromatogram>

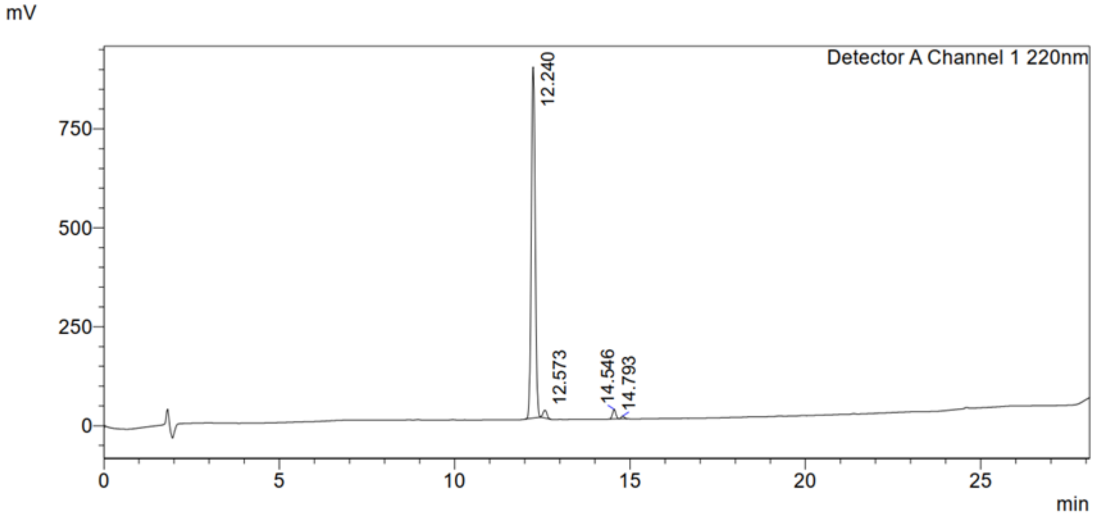

Peak#	Ret. Time	Area	Height	Area%	Height%	Conc.
1	12.240	6758567	887151	95.150	94.582	95.150
2	12.573	148629	19723	2.092	2.103	2.092
3	14.546	154721	24287	2.178	2.589	2.178
4	14.793	41164	6814	0.580	0.726	0.580
Total		7103080	937975	100.000	100.000	

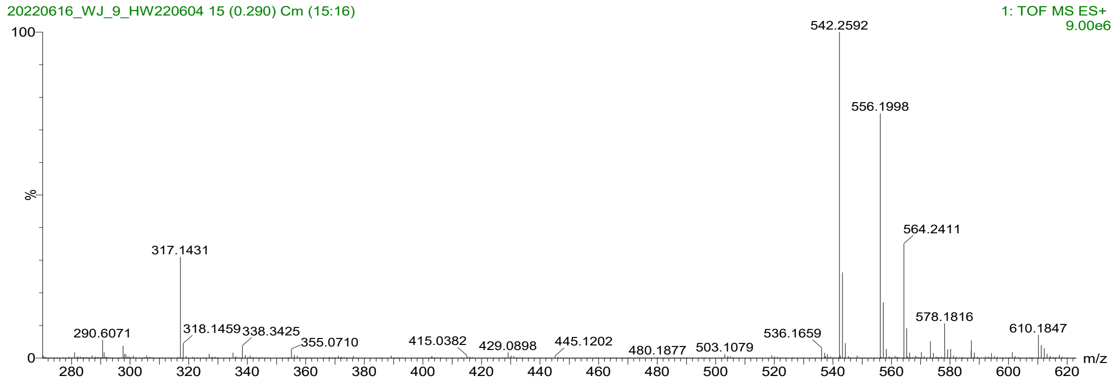

HPLC and HRMS of **H136**

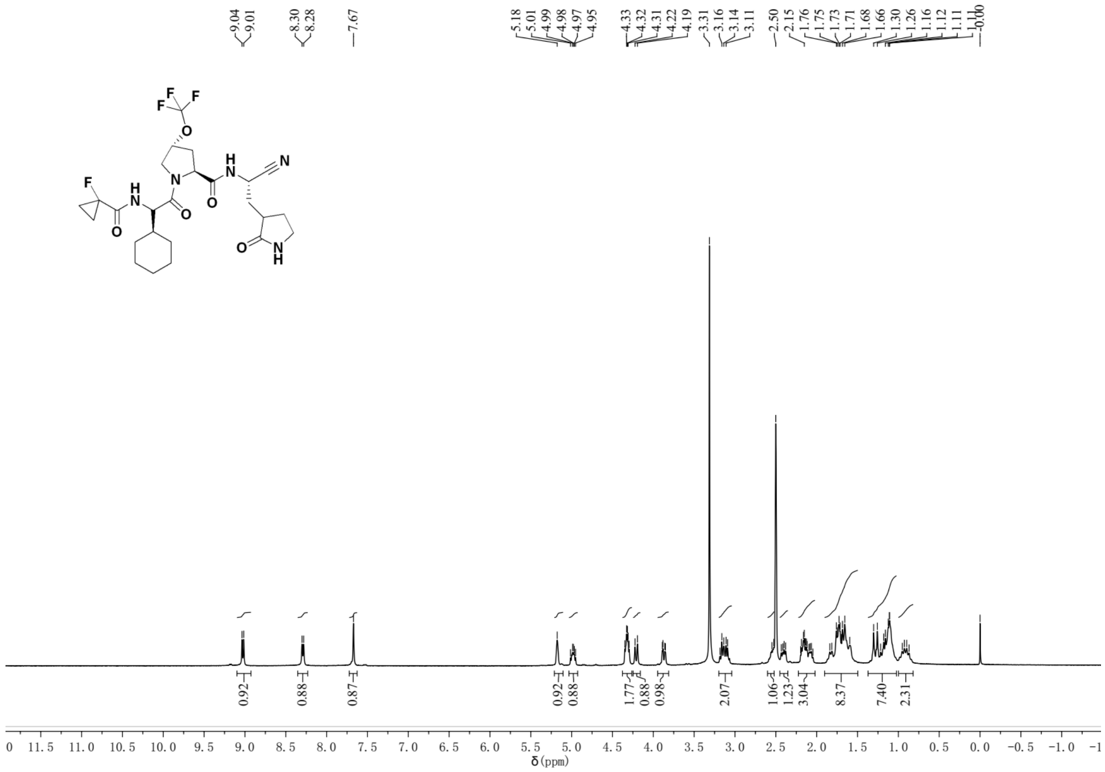
9.04
9.01
8.30
8.28
7.67
5.18
5.01
4.99
4.98
4.97
4.95
4.33
4.32
4.31
4.22
4.19
3.31
3.16
3.14
3.11
2.50
2.15
1.76
1.75
1.73
1.71
1.68
1.66
1.30
1.26
1.16
1.12
1.11
1.11
0.00
0.92
0.88
0.87
0.92
0.88
1.77
0.88
0.98
2.07
1.06
1.23
3.04
8.37
7.40
2.31
δ(ppm)

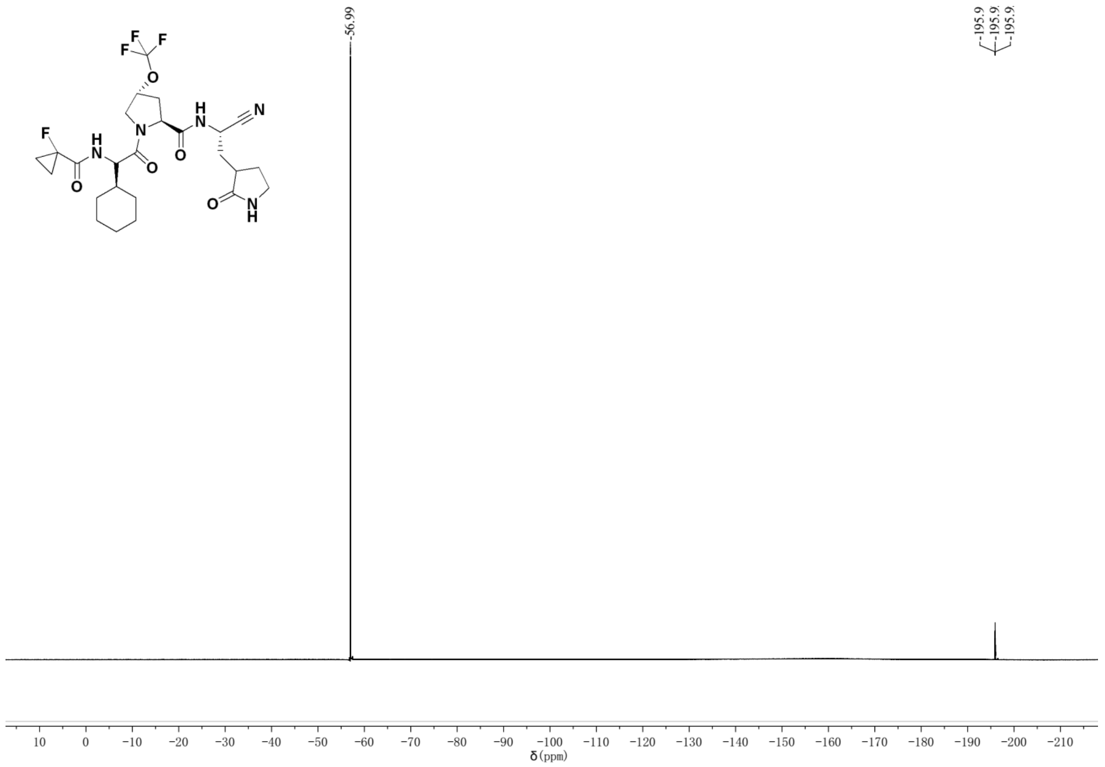
-56.99
-195.9
-195.9
-195.9
δ(ppm)

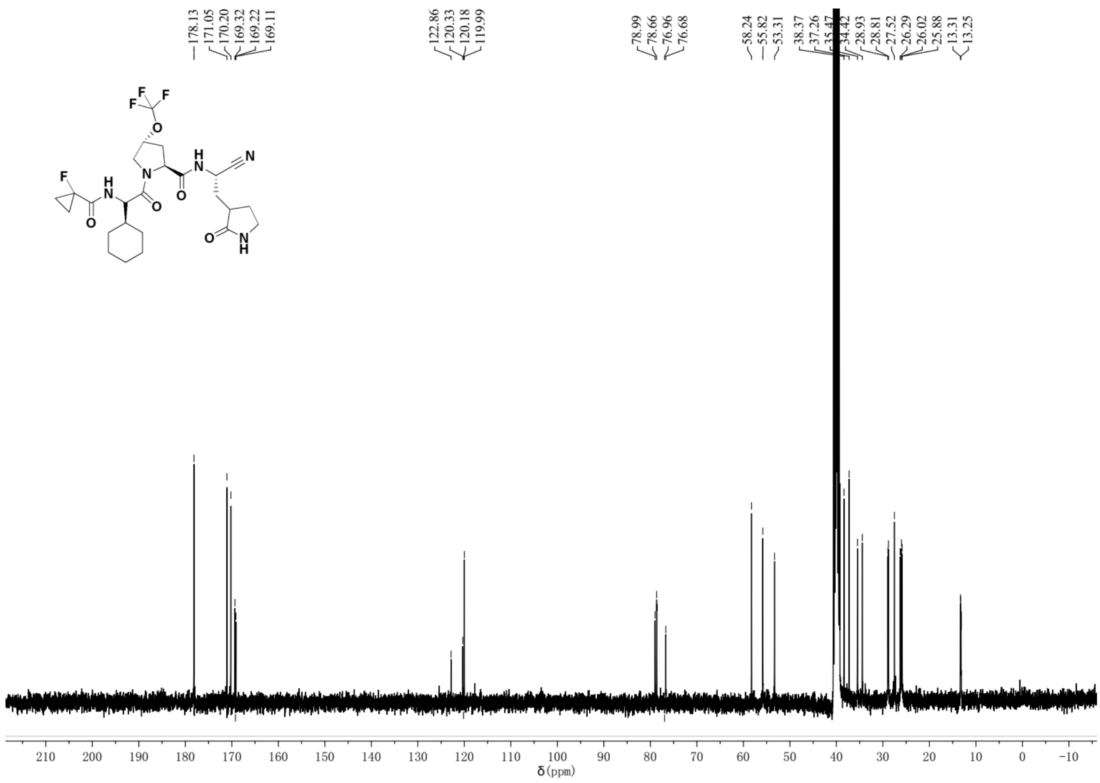

^{1}H NMR, ^{19}F NMR and ^{13}C NMR of **H135**

SHIMADZU LabSolutions

Analysis Report

<Sample Information>

Sample Name	:		
Sample ID	:		
Data Filename	: HW220523-0-2-49A12.lcd		
Method Filename	: proteasome intermediate.lcm		
Batch Filename	:		
Vial #	:	Sample Type	: Unknown
Injection Volume	: 5 uL		
Date Acquired	: 8/4/2022 11:10:50 AM	Acquired by	: System Administrator
Date Processed	: 8/4/2022 11:38:58 AM	Processed by	: System Administrator

<Chromatogram>

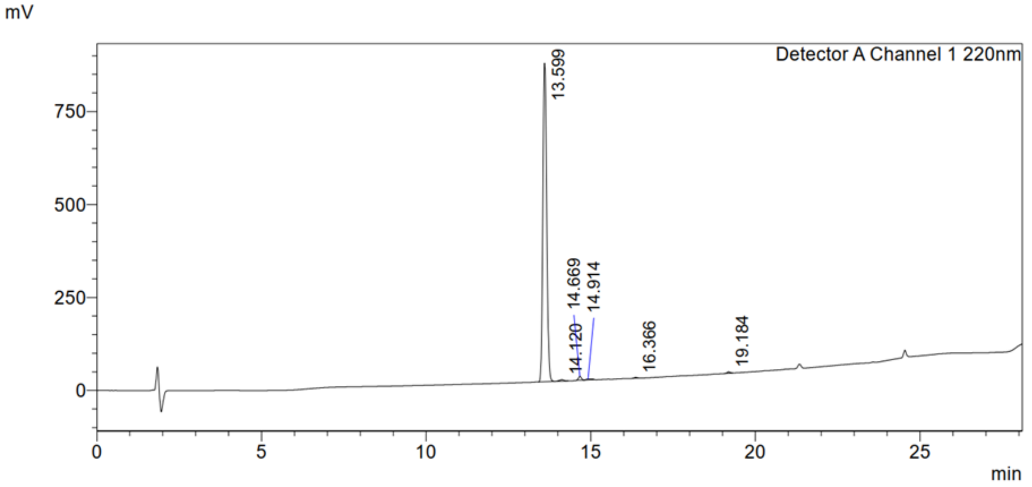

Peak#	Ret. Time	Area	Height	Area%	Height%	Conc.
1	13.599	6837165	856665	97.785	97.515	97.785
2	14.120	30418	3535	0.435	0.402	0.435
3	14.669	59776	10133	0.855	1.153	0.855
4	14.914	24667	2123	0.353	0.242	0.353
5	16.366	13474	2085	0.193	0.237	0.193
6	19.184	26569	3950	0.380	0.450	0.380
Total		6992069	878492	100.000	100.000	

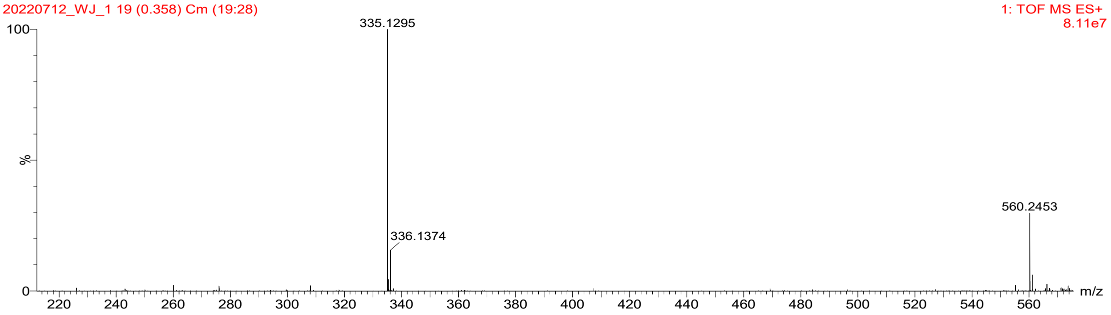

HPLC and HRMS of **H135**

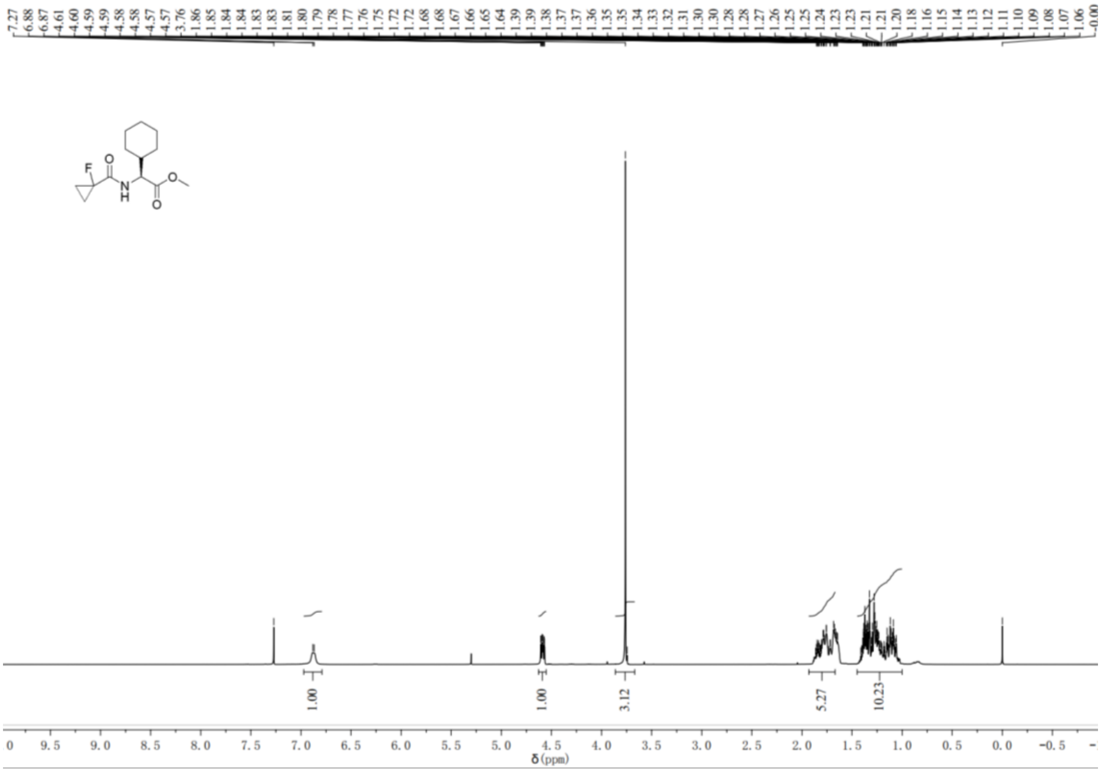

^{1}H NMR of **27**

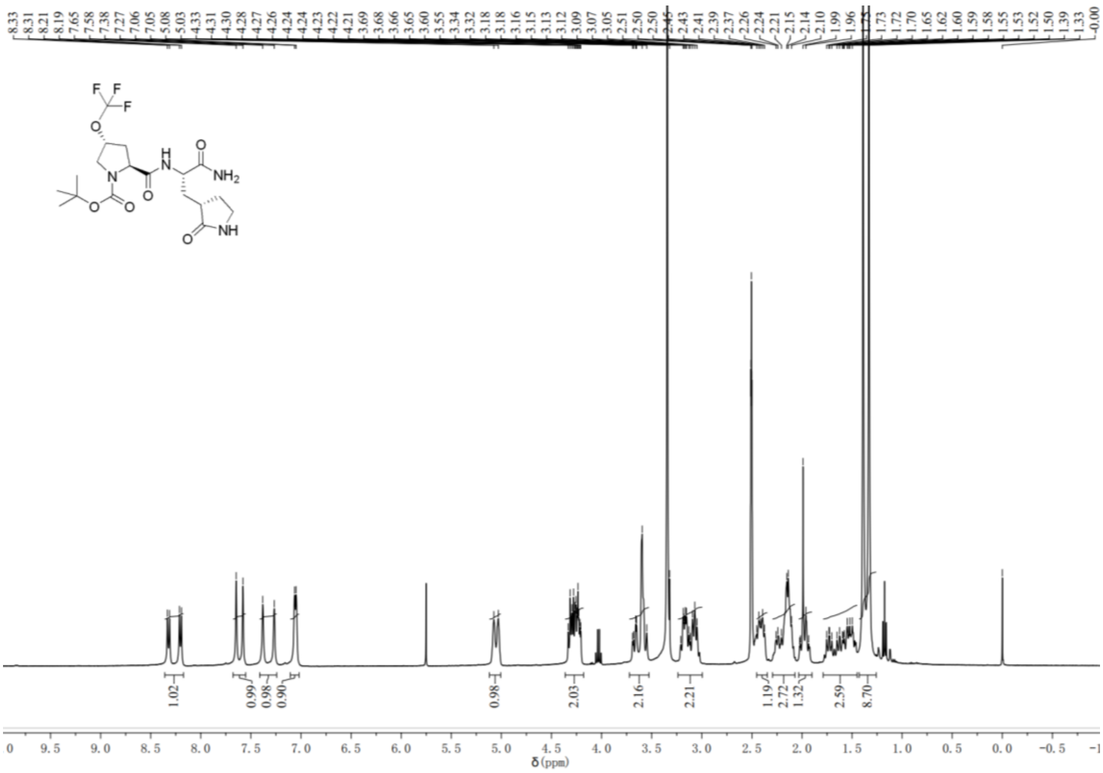

^{1}H NMR of **20d**

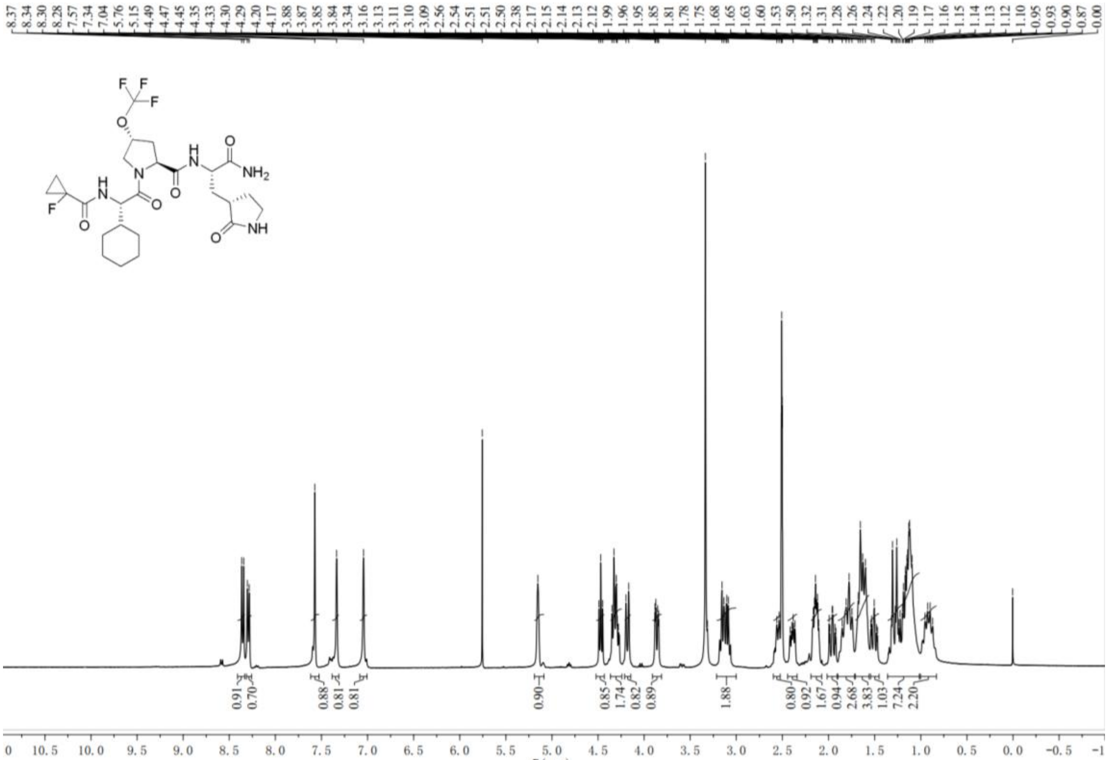

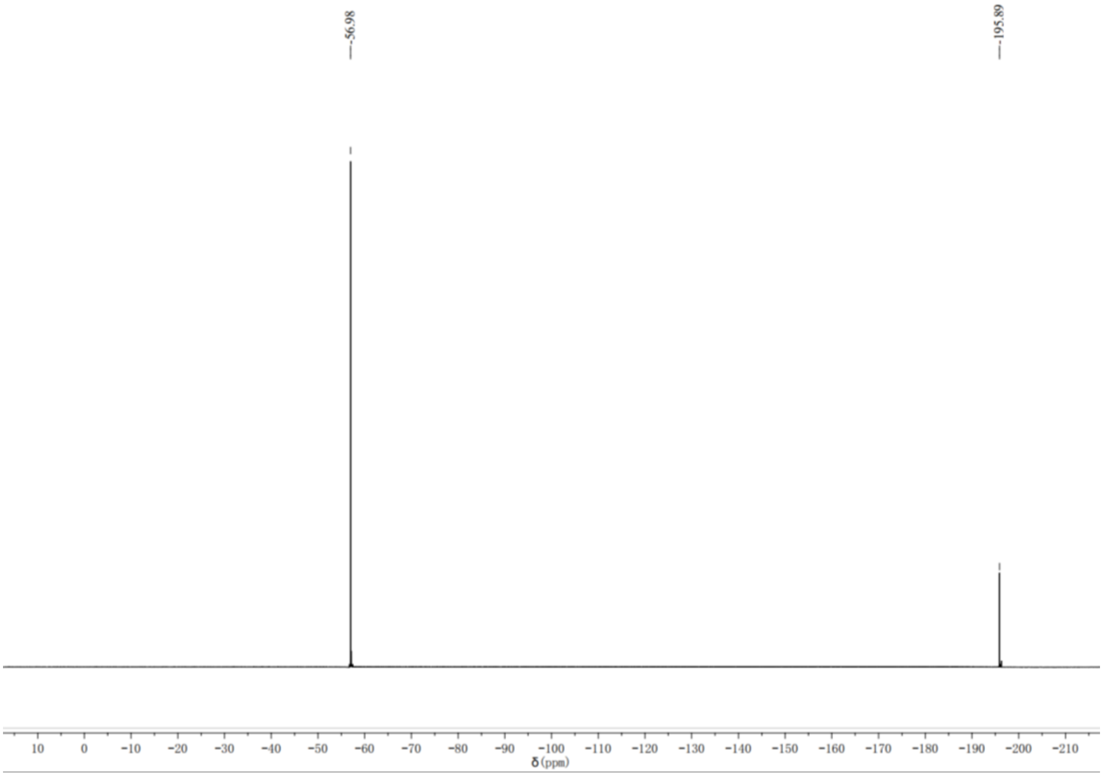

^{1}H NMR and ^{19}F NMR of **22d**

Supplementary References

1. Owen, D. R.; Allerton, C. M. N.; Anderson, A. S.; Aschenbrenner, L.; Avery, M.; Berritt, S.; Boras, B.; Cardin, R. D.; Carlo, A.; Coffman, K. J.; Dantonio, A.; Di, L.; Eng, H.; Ferre, R.; Gajiwala, K. S.; Gibson, S. A.; Greasley, S. E.; Hurst, B. L.; Kadar, E. P.; Kalgutkar, A. S.; Lee, J. C.; Lee, J.; Liu, W.; Mason, S. W.; Noell, S.; Novak, J. J.; Obach, R. S.; Ogilvie, K.; Patel, N. C.; Pettersson, M.; Rai, D. K.; Reese, M. R.; Sammons, M. F.; Sathish, J. G.; Singh, R. S. P.; Steppan, C. M.; Stewart, A. E.; Tuttle, J. B.; Updyke, L.; Verhoest, P. R.; Wei, L.; Yang, Q.; Zhu, Y., An oral SARS-CoV-2 M-pro inhibitor clinical candidate for the treatment of COVID-19. *Science* **2021,** *374* (6575), 1586-1593.

2. Chiba, J.; Takayama, G.; Takashi, T.; Yokoyama, M.; Atsushi Nakayama, A.; Baldwin, J. J.; McDonald, E.; Moriarty, K. J.; Sarko, C. R.; Saionz, K. W.; Swanson, R.; Hussain, Z.; Wong, A.; Machinaga, N., Synthesis, biological evaluation, and pharmacokinetic study of prolyl-1-piperazinylacetic acid and prolyl-4-piperidinylacetic acid derivatives as VLA-4 antagonists. *Bioorganic & Medicinal Chemistry* **2006,** *14* (8), 2725-2746.

3. Levchenko, K.; Datsenko, O. P.; Serhiichuk, O.; Tolmachev, A.; Iaroshenko, V. O.; Mykhailiuk, P. K., Copper-Catalyzed O-Difluoromethylation of Functionalized Aliphatic Alcohols: Access to Complex Organic Molecules with an OCF2H Group. *Journal of Organic Chemistry* **2016,** *81* (14), 5803-5813.

4. Liu, J.-B.; Xu, X.-H.; Qing, F.-L., Silver-Mediated Oxidative Trifluoromethylation of Alcohols to Alkyl Trifluoromethyl Ethers. *Organic Letters* **2015,** *17* (20), 5048-5051.

5. ELLSWORTH, B., A.; JURICA, E., A.; SHI, J.; EWING, W., R.; YE, X.-Y.; WU, X.; ZHU, Y.; SUN, C., Pyrrolidine GPR40 modulators. *WO2014078609* **2014**.

6. Fandrick, K. R.; Patel, N. D.; Radomkit, S.; Chatterjee, A.; Braith, S.; Fandrick, D. R.; Busacca, C. A.; Senanayake, C. H., A Noncoordinating Acid-Base Catalyst for the Mild and Nonreversible Tert-Butylation of Alcohols and Phenols. *Journal of Organic Chemistry* **2021,** *86* (6), 4877-4882.
